# Radiation-induced lymphopenia: A data compilation to unveil relevant factors and mitigation strategies

**DOI:** 10.1016/j.ctro.2025.101071

**Published:** 2025-11-10

**Authors:** Vladislav Sandul, Sarah Salih Al-Hamami, Jiří Kubeš, Marco Durante, Thomas Friedrich

**Affiliations:** a Biophysics Department, GSI Helmholtzzentrum für Schwerionenforschung GmbH, Darmstadt, Germany; b Institute for Condensed Matter Physics, Technical University Darmstadt, Darmstadt, Germany; c Department of Radiation Oncology, Proton Therapy Center, Prague, Czechia; d Department of Clinical and Experimental Oncology, 2nd Faculty of Medicine, Charles University, Prague, Czechia

**Keywords:** Radiation-induced lymphopenia, Lymphocyte count, Proton therapy, Particle therapy, Immunotherapy

## Abstract

**Purpose:**

Radiation-induced lymphopenia (RIL) is a common complication of radiation therapy (RT) that can undermine antitumor immunity and diminish the efficacy of immunotherapy. While RIL is a known predictor of poor outcomes, its underlying mechanisms and key determinants remain poorly characterized.

**Materials and Methods:**

We systematically compiled and quantitatively analyzed published data on absolute lymphocyte count (ALC) dynamics during and after RT. A total of 142 ALC curves from 52 publications were digitized and integrated into a standardized database. This database encompasses various RT modalities, including therapy with X-rays or charged particles, as well as extracorporeal irradiation of blood (ECIB).

**Results:**

Analysis revealed consistent lymphocyte depletion during treatment. By the end of RT, median ALCs declined to 24% (range: 1.5–78%) of baseline for intracorporeal exposure and 10% (range: 1–60%) for ECIB. Recovery was incomplete and cancer-type-dependent, reaching only a median of 55% (range: 33–90%) of baseline within one year. We identified baseline ALC, planning target volume (PTV), and dosimetric parameters as key predictors of severe lymphopenia. A low rate of lymphocyte depletion during treatment was frequently associated with impaired long-term recovery. Particle therapy was associated with an average 11% higher end-of-treatment ALC compared to photon therapy, underscoring the lymphocyte-sparing benefit of reduced integral dose.

**Conclusions:**

This work establishes a comprehensive, quantitative foundation for understanding RIL. The developed ALC database supports mechanistic investigation and enables model-based approaches to predict lymphocyte dynamics, guiding the design of lymphocyte-sparing treatment strategies, including particle therapy.

## Highlights

• A database of 142 ALC curves from 52 publications is presented for the study of trends of lymphopenia and model benchmarking.
• Distinct cancer-site-dependent patterns in lymphocyte depletion during RT and post-treatment recovery are observed and analyzed.
• Baseline ALC, PTV, and dose are identified as key predictors of RIL severity.
• Proton and carbon therapies are associated with less severe lymphopenia than photons.
• Findings support the development of strategies to preserve immunity during RT.

## Introduction

In the realm of cancer treatment, the intricate interplay between the immune system and tumorigenesis has become increasingly evident. The immune system's pivotal role in cancer surveillance underscores the importance of understanding its interactions with therapeutic modalities such as radiation therapy (RT) [[1], [2], [3]]. One notable consequence of RT is the induction of radiation-induced lymphopenia (RIL), characterized by a depletion of lymphocytes crucial for mounting an effective antitumor immune response [[4], [5], [6]]. Although RIL has long been observed, its clinical implications have gained prominence only in recent years [7], as studies across various solid tumors have highlighted its association with compromised treatment outcomes [5,[8], [9], [10], [11], [12], [13], [14]].

Over the past 15 years, the origins and severity of RIL have been investigated using various modeling approaches aimed at elucidating its underlying mechanisms and indentifying potential interventions. A comprehensive review by Cella et al. [15] analyzed 19 modeling studies published between 2018 and 2023, concluding that despite diverse frameworks, none have yet been adopted in clinical practice. Paganetti [16] provided a broader perspective, incorporating additional modeling approaches and emphasizing the complexity of dose-to-lymphocyte calculations due to factors such as lymphocyte recirculation between the blood and secondary lymphoid tissues.

Despite the diversity of approaches and findings regarding factors influencing lymphopenia, most studies underscore the critical role of dosimetric and volumetric parameters, including target dose, dose rate, total body dose, the volume of normal tissue receiving a specific dose (VN), field size, planning target volume (PTV), and baseline absolute lymphocyte count (ALC) before therapy [6,[16], [17], [18], [19], [20]]. Additionally, certain organs, such as the liver, heart, spleen, and bone marrow (BM), are identified as organs-at-risk associated with the development of RIL [[16], [17], [18], [19], [20], [21], [22]]. However, no consensus has yet been reached on the relevance of irradiating specific organs in the development of RIL [16,20].

The choice of RT modality, including photon and particle therapy, is believed to play a significant role in shaping the dynamics of RIL. Moreover, combining RT with immunotherapy has emerged as a promising strategy to harness the immune system's antitumor potential for enhanced therapeutic efficacy [[23], [24], [25], [26], [27], [28], [29], [30]]. While photon therapy remains the cornerstone of RT, particle therapy, using protons or carbon ions, has gained traction for its potential to spare surrounding healthy tissues [[31], [32]] and to mitigate adverse effects, including RIL [[33], [34], [35], [36], [37], [38]].

To assess existing hypotheses and critically evaluate their underlying assumptions, a thorough examination of clinical data is required. The goal of the present work is to advance current knowledge on RIL by investigating the characteristics of clinical ALC data during and after RT, with a focus on studies directly addressing ALC dynamics. The study aims to explore the complex determinants and underlying factors influencing the onset of RIL and to identify potential strategies and interventions for mitigating its occurrence and severity. To achieve this, we present a comprehensive analysis of published data on ALC dynamics in patients with RIL, supported by a database compiled from ALC curves extracted from the literature. Through qualitative analysis of these data, observed patterns are examined and open questions related to RIL are addressed, with the goal of refining, validating, and informing dedicated model approaches [15,[39], [40]] that may improve treatment outcomes—such as overall survival (OS), progression-free survival (PFS), and disease-free survival (DFS)—and post-treatment recovery in patients.

## Methods and materials

### Development of a database for ALC dynamics

A broad range of treatment modalities was reviewed and categorized into two distinct groups. The first group represents intracorporeal exposure (ICE), with a range of RT techniques. These include photon-based therapies, including conformal RT (2D-CRT, 3D-CRT), intensity-modulated RT (IMRT), volumetric modulated arc therapy (VMAT), helical tomotherapy (HTT), stereotactic body RT (SBRT), and total body irradiation (TBI), as well as proton beam therapy (PBT) and carbon ion RT (CIRT). The second group covers extracorporeal irradiation of blood (ECIB) with photon radiation, a method used during the 1960 s and 1970 s, primarily for the treatment of leukemia and in the context of renal transplantation.

The reviewed publications were primarily identified through PubMed and Google Scholar. The first group was collected using the search terms “radiotherapy” and “lymphopenia,” and the second group used the term “extracorporeal blood irradiation.” Only English-language publications were considered, except for Meuret et al. [41], which is in German. The reference lists of the selected articles were also examined to identify additional relevant references. Articles were included if they contained data on the dynamics of absolute or relative lymphocyte counts (RLC) during and, if available, after ICE or ECIB. RLC refers to the ALC normalized to its pretreatment (baseline) value and expressed as a percentage. The data were provided as plots showing the relationship between ALC or RLC and time or received dose.

Following these criteria, a total of 67 studies were compiled [7,[10], [11], [12],^19^,^21^,[33], [34],^36^,^38^,[41], [42], [43], [44], [45], [46], [47], [48], [49], [50], [51], [52], [53], [54], [55], [56], [57], [58], [59], [60], [61], [62], [63], [64], [65], [66], [67], [68], [69], [70], [71], [72], [73], [74], [75], [76], [77], [78], [79], [80], [81], [82], [83], [84], [85], [86], [87], [88], [89], [90], [91], [92], [93], [94], [95], [96], [97]], including 58 on ICE [7,[10], [11], [12],^19^,^21^,[33], [34],^36^,^38^,[42], [43], [44], [45], [46], [47], [48], [49], [50], [51], [52], [53], [54], [55], [56], [57], [58], [59], [60], [61], [62], [63], [64], [65], [66], [67], [68], [69], [70], [71], [72], [73], [74], [75], [76], [77], [78], [79], [80], [81], [82], [83], [84], [85], [86], [87], [88], [89]] and 9 on ECIB [41,[90], [91], [92], [93], [94], [95], [96], [97]], published between January 1970 and October 2024. Detailed summaries of these publications are provided in Tables A1 and A2 of the supplementary material for publications on ICE and ECIB respectively, categorized by cancer type, treatment modality (e.g., RT techniques, use of combined therapy, doses, and fractionation scheme), and the number of patients. These tables also include information on the estimated RLC at the end-of-treatment (EoT-RLC), lymphocyte count recovery in the blood after RT, and the main conclusions regarding lymphopenia reported in the studies. The estimated EoT was determined from treatment details from publications, such as prescribed dose and fractionation scheme; a more detailed description of the estimation procedure for therapy duration is available in the supplementary material (section 2, Fig. A1). The EoT-RLC was assessed as the RLC of the data point closest to the estimated EoT.

The ALC curves (i.e. values of ALC/RLC vs time or dose) extracted from the selected studies were digitized using WebPlotDigitizer [98], forming a comprehensive ALC database. This database is available on the GSI ALC database homepage http://www.gsi.de/bio-alc for download.

However, not all of the publications listed in Tables A1 and A2 were included in the database. In some cases, ALC data could not be extracted due to difficulties distinguishing overlapping ALC curves [43,86], or because of unclear axis scaling [21,[84], [85],[87], [88],[94], [95], [96], [97]]. Furthermore, datasets from some publications [7,19,38,89] were excluded because they duplicated or reproduced data already included in other sources. The final database comprises 142 entries (i.e. datasets representing ALC curves) from 52 publications. The distribution of publication years for these 52 studies is shown in Fig. A2 of the supplementary material.

### Data Processing and Analysis

To utilize the database for a qualitative analysis, datasets were filtered to exclude the following cases:

1. Datasets for individual patients were excluded because of high fluctuations in data points, which could obscure overall trends [[41], [42],[55], [56],[90], [91], [92]].
2. In cases where combined (pooled) datasets, typically representing all patients in a study, consisted of a number of uncombined datasets (subsets), only one of both options was selected for further processing in order to avoid duplicate information.
  (i) The pooled dataset was retained, and the subsets were excluded when the subsets were based on factors such as post-treatment OS [69], RIL grade [65], cancer stage [59], baseline ALC [65,76], or tumor-unrelated disease status [64].
  (ii) Conversely, pooled datasets were omitted if more specific datasets, stratified by radiation modality or cancer type, were available as subsets [34,57,61,80].
3. Datasets where ALC levels were given as a function of accumulated dose instead of time were excluded [93].

The retained datasets formed the core dataset pool that served as the basis for all subsequent analyses.

### Data processing for lymphocyte dynamics analysis

To analyze lymphocyte dynamics during RT, datasets containing at least three data points within the treatment period were selected from the core dataset. To assess lymphocyte recovery after treatment, only datasets with at least one post-therapy data point were included.

To explore the relationship between RLC depletion rate and post-treatment recovery, we selected datasets containing at least three data points during therapy and at least one after treatment. Additionally, the datasets from Mohan et al. [36] were excluded because of their atypical initial RLC increase. An exponential fit was applied to the treatment-period data of each selected dataset using the following equation:

$$RLC\left(t\right)=\left(1-c\right)\cdot exp\left(-bt\right)+c$$

where *t* represents time, and *b* and *c* are free parameters. The term (1-c) ensures that $RLC\left(0\right)=1$. As described by Ellsworth et al. [99], the parameter *b* represents the lymphocyte depletion rate, quantifying the rate of lymphocyte decline over time. The parameter *c* corresponds to the nadir plateau level of RLC, indicating the minimum reached before potential recovery.

As the majority of studies reported ALC dynamics versus time rather than delivered dose, *t* was used as the independent variable to maintain consistency with the published data and avoid introducing uncertainty from dose–time conversions. This formulation reflects mean ALC trends in cohorts treated on standard weekday fractionation schedules and should be interpreted as describing average depletion over consecutive treatment weeks rather than detailed day-to-day kinetics.

### Data processing for baseline–EoT ALC analysis

Starting from the core dataset pool, datasets missing values for either baseline ALC [12,36,42,[79], [80],^91^,^93^] or EoT-ALC [81] were excluded to enable analysis of the relationship between baseline and end-of-treatment ALC.

The retained data points were grouped by cancer site and clustered within 95 % confidence ellipses if the group contains three or more data points. Each ellipse was centered at the sample mean of the group, with principal axes determined from the eigenvectors of the covariance matrix. The semi-axes were scaled by $\sqrt{5.991}$, the 95th percentile of the chi-squared distribution with 2 degrees of freedom, to enclose the true mean with 95 % confidence, assuming the data arise from two independent Gaussian variables [100].

## Results

### Database properties

Table 1 provides a general overview of the database. It reflects the currently available data across the various settings as described below.

**Table 1:** Overview of the main features of the developed ALC database. It includes information on the number of datasets in the database with and without post-treatment ALC recovery data. It displays the number of uncombined and combined datasets, where the latter represents pooled data from other datasets (e.g., all patient datasets combined from patient subgroups). Likewise, the number of datasets representing ALC curves for individual patients or patient cohorts is given. The table also provides statistical data on irradiated sites, radiation type, patients numbers (excluding single-patient datasets), the average number of data points per dataset, the estimated EoT, and the RLC at the data point closest to the estimated EoT timepoints.

# of Datasets with recovery data / without uncombined / combined single patients / cohorts	142 (taken from 52 publications) 66 / 76 132 / 10 57 / 85

Irradiated site	Digestive/Gastrointestinal (75), Hematological/Blood (19), Head/Neck (16), Genitourinary and Gynecologic (9), CNS (8), Respiratory/Thoracic (8), Breast (2), Musculoskeletal (2), Total body (1), Mixed (2)
Radiation type	Photon therapy (101) (including ICE (81), ECIB (19) and TBI (1)), Proton therapy (18), Carbon therapy (2), Mixed photons / protons (21)
# Patients in cohorts [median (range)]	95 (4–920)
# Datapoints [median (range)]	7 (3–81)
Estimated therapy duration in weeks [median (range)]	5.6 (0.57–15)
Estimated RLC at EoT time in % [median (range)]	22.8 (0.9–78.3)

The studies included in the database encompass multiple cancer types and are organized by anatomical sites in Table A3 in the supplementary material. Information on EoT-RLC and post-treatment RLC recovery is provided for each site. Cervical and prostate cancer are included in the category of pelvic cancers here and throughout this work.

Statistics on the type of RT are presented in Table A4 in the supplementary material. IMRT was most frequently used, followed by proton therapy. Chemotherapy was mentioned in 42 of the 47 ICE publications.

After the multistep selection process described in the Methods section, 61 datasets from 44 publications were retained from the initial pool of 142 entries, and constituted the core dataset pool for this study.

### Radiotherapy doses and fractionation

RT target doses and fractionation schemes varied significantly among the studies. For ICE, doses ranged from 41 to 70 Gy, with one exceptional case reported by De et al. [70] where doses ranged from 30 Gy to as high as 100 Gy (for ion therapy, doses were adjusted for relative biological effectiveness (RBE)). Standard fractionation schemes involved 1.8 to 3 Gy per fraction over 5 to 7 weeks, totaling 25 to 35 fractions. However, several studies [70,78] adopted hypofractionation, using 12 to 15 fractions.

For ECIB, doses ranged from 90 [92] to 546 [93] Gy with transit doses ranging from 2.8 to 8 Gy. “Transit dose” refers to the dose that the blood receives while circulating outside the body through an irradiation device during ECIB. In ECIB, the blood itself is the target, and the reported values represent the mean dose delivered to it.

### Patterns in lymphocyte count dynamics

Of the 52 reviewed studies of the database, all report a decrease in RLC during RT, with reductions to a median (range) of 24 (1.5–78)% for ICE and 10 (1–60)% for ECIB among all datasets, with an interquartile range of (16.0 %, 33.5 %).

A total of 34 studies provided data on post-treatment ALC recovery. Post-RT trends indicated gradual lymphocyte replenishment, with ALC generally returning to 40–80 % of baseline levels within one year. Full recovery to baseline was not observed within the typical observation periods, with the highest recovery reaching up to 93–96 % of initial ALC over 5–10 years [68,81].

The analysis of lymphocyte dynamics over time revealed trends among different cancer types. Four distinct patterns were identified:

• **Brain tumors, breast cancer,** and **bone metastasis from hepatocellular carcinoma** exhibited the highest typical EoT-RLC values of all tumor entities (median 62 %, 60 %, and 53 %, respectively). These cancers also showed the highest post-treatment recovery of RLC, reaching 50–90 % of baseline levels within one year.
• **Pancreatic cancers** and **soft tissue cancers** had relatively high typical EoT-RLC values (34 % and 36 %, respectively). Pancreatic cancers showed post-treatment RLC recovery of 40–70 % within one year, while soft tissue cancers approached 80 % within that period.
• **Anal cancer, esophageal, head and neck, liver, lung, and pelvic cancers** had typical EoT-RLC values of 20–30 %. Esophageal and liver cancers demonstrated strong recovery, reaching 50–85 % of initial ALC within one year, whereas the remaining cancers in this group show a modest ALC recovery, ranging from 40 % to 70 %.
• **ECIB** had the lowest EoT-RLC values, with a median of 10 %, while variability of values was high (from 1 % to 70 %). Weeke et al. [93] reported RLC recovery from 23 % to 37 % within 14 weeks.

Despite much higher doses delivered to the blood, the RLC nadirs for ECIB did not differ significantly from those observed in ICE, generally ranging from 10 % to 30 %. TBI for BM transplantation led to a sharp drop of RLC to values of almost 0 % after 8 fractions (4 days of 2 daily fractions) [79], as expected.

RLC over time typically exhibited a rapid, exponential-like drop to the nadir, which was reached within a few weeks, usually close to the EoT. Subsequently, RLC levels remained relatively constant, suggesting a balance between lymphocyte depletion and replenishment. The typical weekly drop rate ranged from 20 % to 50 %. An exception was reported by Mohan et al. [36], who observed an initial increase in RLC following the start of treatment for both photon and proton therapy.

### Clinical outcomes and predictive factors for RIL

Of the 47 studies on ICE, 30 conclude that severe lymphopenia is linked to poorer outcomes, including OS, PFS, DFS, and related measures across various cancer types. However, Ng et al. [66], Campian et al. [64], and Schad et al. [81] reported no significant association with survival outcomes. Additionally, Cho et al. [72] found that lymphocyte recovery following chemoradiotherapy was associated with improved outcomes in patients with non-small cell lung cancer (NSCLC).

The primary predictors of lymphopenia identified were baseline ALC and PTV size. Female sex [36,46,82], high body mass index (BMI) [63], and greater values of specific dosimetric parameters (e.g., V5Gy, V10Gy, mean body dose) were also associated with increased risks and RIL severity. Proton and carbon ion therapies are associated with less severe lymphopenia compared to photon therapy [[33], [34],^36^,[54], [55],^57^,^59^,^61^,^70^,^73^].

Of the 47 reviewed ICE publications, 42 mention concurrent chemotherapy, but only 4 studies [34,45,52,74] explicitly observed a positive correlation between chemotherapy and the severity of RIL. In contrast, 5 authors [[10], [11],^71^,^77^,^79^] reported no significant impact of chemotherapy on ALC levels. Additionally, Chadha et al. [87] noted that radiation had a greater effect on ALC than chemotherapy, while Ellsworth et al. [79] observed that the greatest ALC loss occurred in groups without concurrent chemotherapy.

Among the reviewed publications, only Kim et al. [46] investigated the influence of hypofractionation on RIL severity. The study reported that hypofractionated irradiations provided comparable oncologic outcomes to conventional fractionation but resulted in smaller changes in ALC per fraction.

### RLC dynamics during treatment and recovery

**Fig. 1:**
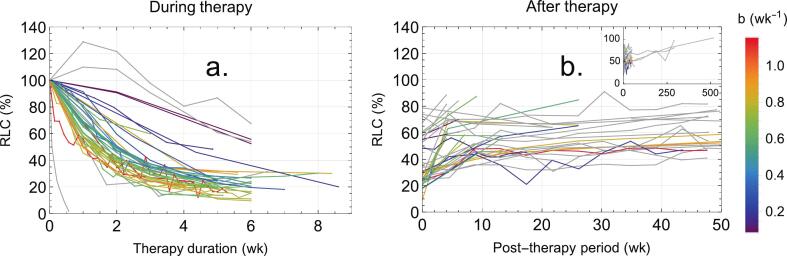
Dynamics of RLC (a) during RT and (b) after RT. The inset in panel (b) presents the same data with the time axis extended to 550 weeks to illustrate long-term trends. The color bar represents the lymphocyte depletion rate *b*, with individual curves color-coded accordingly.

Fig. 1, Fig. 1 depict the temporal dynamics of relative lymphocyte count (RLC) during and following RT, respectively. From the 61 datasets comprising the core dataset pool (see Materials and Methods), 44 datasets with intratherapy measurements were used to generate Fig. 1a, while 42 datasets containing post-therapy data were used in Fig. 1b.

In Fig. 1a, most datasets exhibited an approximately exponential decline in RLC during treatment, except for two datasets from Mohan et al. [36], which displayed an atypical initial increase in RLC. Fig. 1b illustrated the typical post-treatment recovery pattern: an initial rapid increase in RLC, followed by a plateau or a very gradual recovery phase, with full recovery typically taking 5–10 years.

**Fig. 2:**
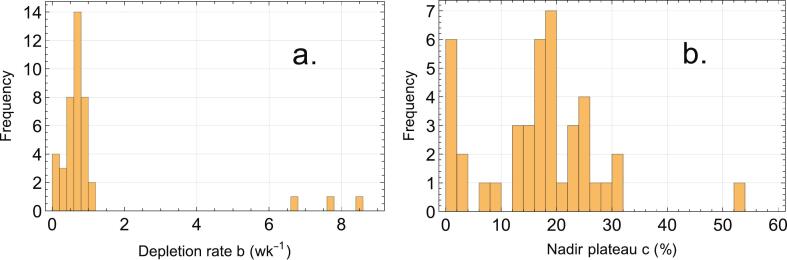
Distribution of lymphocyte depletion rates and nadir plateau levels during RT. Histograms of lymphocyte depletion rates during RT (a) and nadir plateau levels (b), derived from exponential fits to RLC data during treatment.

The distributions of the parameters *b* and *c* of the exponential fits of the datasets are presented in Fig. 2, with a median depletion rate *b* of 0.67 wk^-1^ and a median plateau level *c* of 17 %. In addition, plots of individual dataset fits are provided in Fig. A3 of the supplementary material.

The curves in Fig. 1, Fig. 1 were color-coded according to the corresponding *b* value from the exponential fit, reflecting the steepness of RLC decline. Datasets that were not fitted or whose *b* values were identified as outliers [[51], [52],^68^] are shown in gray. No significant association was observed between the depletion rate during treatment and the recovery rate.

### Baseline and End-of-Treatment ALC analysis

**Fig. 3:**
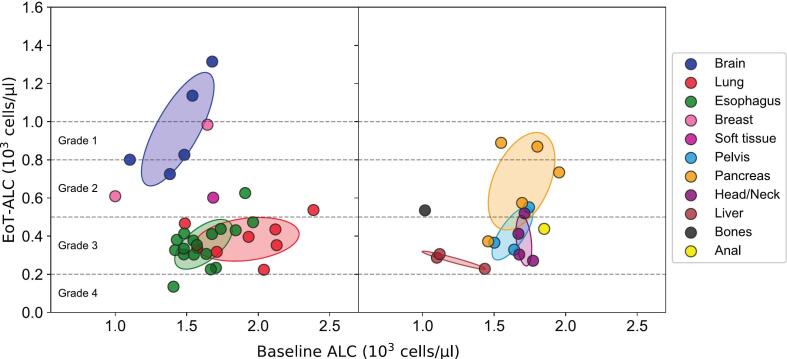
EoT-ALC versus baseline ALC for 50 selected datasets. Points represent ALC datasets, grouped by cancer site and visualized with 95% confidence ellipses for groups containing three or more datasets. Data are split into two panels for improved clarity and cluster distinction. Dashed lines indicate the ALC thresholds for specific RIL grades.

Fig. 3 shows a scatter plot of baseline versus EoT-ALC. Each of 50 data points represents the baseline and EoT-ALC for a group of patients, typically given as an average or median, since individual patient data were excluded. To enhance clarity and improve visual distinction between clusters, data were split across two panels.

The baseline ALC values for the analyzed datasets ranged from 1.0∙10^3^ to 2.5∙10^3^ cells/μl, with a median of 1.6∙10^3^ cells/μl. The EoT-ALC ranged from 0.1∙10^3^ to 1.4∙10^3^ cells/μl, with a median of 0.4∙10^3^ cells/μl. Most patient groups experienced grade 3 RIL.

Different cancer sites were associated with distinct ranges of baseline and EoT-ALC values, with varying correlation between them. Detailed information on the median values, range intervals of baseline and EoT-ALC, and correlation coefficients for each cancer site is provided in Table A5 of the supplementary material.

Patient groups with brain tumors, liver cancer, breast cancer, and bone metastases from hepatocellular carcinoma exhibited relatively low baseline ALC values, with medians ranging from 1.0∙10^3^ to 1.4∙10^3^ cells/μl. In contrast, patient groups with cancers located in the pancreatic region, esophagus, head and neck, anal, and soft tissue regions generally showed median baseline ALC values between 1.6∙10^3^ and 2.0∙10^3^ cells/μl. The group with lung cancer displayed a broader baseline ALC range, spanning from 1.5 ∙10^3^ to 2.4∙10^3^ cells/μl.

Despite having the lowest baseline ALC values, the brain and breast cancer groups were notable for their relatively high EoT-ALC levels, with a median of 0.8∙10^3^ cells/μl, corresponding to RIL grades 1 and 2. Pancreatic cancer groups showed substantial variability in EoT-ALC values, ranging from RIL grade 1 to grade 3. Groups with bone metastases from hepatocellular carcinoma and soft tissue cancers were generally associated with RIL grade 2. Other groups tended to experience more severe post-treatment RIL (grade 3 or higher), with particularly low EoT-ALC observed in the liver cancer group. The median EoT-ALC in this group was 0.3∙10^3^ cells/μl, approaching the grade 4 threshold.

While visual inspection of clusters suggested a possible positive correlation between baseline and EoT-ALC for specific groups, statistically significant correlations (p-value < 0.05) were observed only for the esophageal cancer group with a Pearson correlation of 0.6. Additional data would be needed to confirm correlations in other groups.

### Comparison of EoT-RLC values between photon and ion therapies

**Fig. 4:**
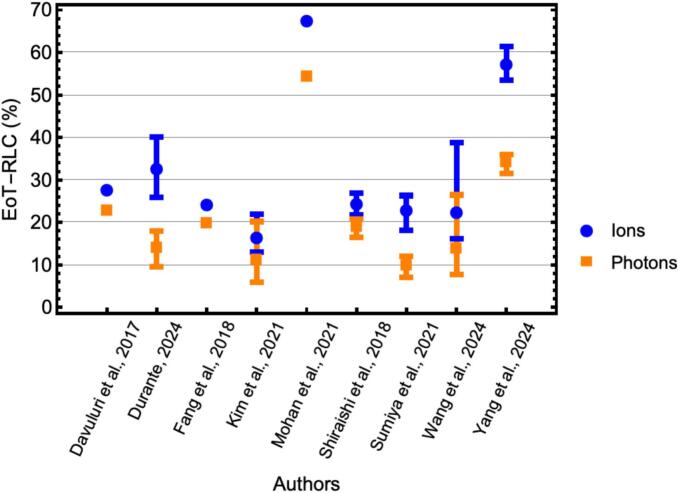
Comparison of EoT-RLC values between photon and ion therapies. EoT-RLC values from multiple studies comparing photon therapy and ion therapy are presented. Error bars are included where available in the original publications. Error bars represent standard deviations, either reported directly or calculated from reported interquartile ranges or confidence intervals, and are omitted if information on uncertainties was unavailable or unclear.

We analyzed studies in which ALC dynamics in patients treated with photons versus those treated with ion therapy (protons or carbon ions) are compared [[33], [34],^36^,^54^,^57^,^59^,^61^,^73^,^78^]. The EoT-RLC values were compared in Fig. 4. EoT-RLC values were consistently lower for photon treatments than for ion therapy. The average absolute difference in EoT-RLC between ion and photon therapies was 11 %, while the average relative increase in EoT-RLC for ion therapy compared to photon therapy was 63 %.

## Discussion

### Phenomenology of ALC dynamics

The adverse relationship between RIL severity and prognosis may be attributed to the critical role of lymphocytes in the immune response. Lymphocyte depletion increases patient susceptibility to infections and impairs the immune system's ability to mount an effective antitumor response [2,[101], [102]].

However, this correlation does not imply causation, and may instead result from independent consequences of treatment-specific factors. More aggressive and advanced tumors naturally have worse prognoses and often necessitate a larger PTV and higher radiation doses, increasing radiation damage to the immune and lymphopoietic systems. Lymphocytes, being highly radiosensitive [16,[103], [104]], are significantly affected, leading to RIL. Thus, both worse prognosis and the occurrence of RIL may result from treatment of more advanced-stage tumors.

Investigating the relationship between baseline ALC and treatment outcomes could help determine whether this association reflects correlation or causation. Several studies reported that higher pre-treatment ALC is associated with improved outcomes in specific tumor sites. In pancreatic adenocarcinoma, Heo and Noh [76] found correlations with OS, locoregional failure-free survival (LRFS), and distant metastasis-free survival (DMFS). Similar trends were observed in hepatocellular carcinoma [45] and cervical cancer [51], as well as in multiple myeloma treated with CAR-T [105] and lung cancer receiving combined immunotherapy and RT [106]. In contrast, Zhou et al. [63] found no significant link between pretreatment lymphopenia and outcomes in esophageal cancer, and Conroy et al. [107] also reported no OS or PFS differences between low- and normal-ALC groups. Thus, while baseline ALC may serve as a prognostic factor in some tumor sites, its predictive value across cancers remains inconclusive.

Cancers in different anatomical sites exhibited distinct baseline ALC medians and ranges. Certain tumors can create an immunosuppressive environment, reducing lymphocyte counts and potentially leading to lower baseline ALC [108]. Additionally, baseline ALC tends to decline with age, suggesting that cancers more common in older populations might be associated with lower baseline ALC. This is presumably related to immunosenescence in the elderly [[109], [110], [111]].

The EoT-ALC values also varied by cancer site, with most patient groups having values between 0.2∙10^3^ and 0.5∙10^3^ cells/μl, corresponding to RIL grade 3. Despite variability in baseline ALC, treatment modality, and cancer type, EoT-ALC rarely dropped below 0.2∙10^3^ cells/μl.

### Shape of ALC curves and potential mechanisms of RIL

In all reviewed studies, a steep, exponential-like decline to a nadir point in ALC/RLC was observed during RT (see Fig. 1a). Typical estimated EoT-RLC values ranged from 20 % in lung and esophageal cancers to 60 % in glioblastoma and breast cancer, with most cancer types generally showing values between 20 % and 35 %.

The exponential decrease as reflected by equation (1) was consistent with previous studies [55,74,79,95]. The distributions of the fitted parameters *b* (lymphocyte depletion rate) and *c* (nadir plateau level) are shown in Fig. 2, with median *b* and *c* values of 0.67 wk^−1^ and 17 %, respectively. The consistent lower limit in EoT-RLC, despite variations in tumor biology and treatment protocols, suggested the existence of a physiological threshold for lymphocyte depletion. This non-zero nadir may reflect inherent immune system resilience, a balance between lymphocyte loss and recovery, where radiation-induced depletion is counteracted by immune cell replenishment.

The post-treatment RLC recovery typically followed a pattern: levels rose to 40–70 % within 1–6 months post-treatment and then showed a very slow recovery thereafter (see Fig. 1b). Two possible hypotheses could explain this dynamic pattern: First, lymphocytes can be roughly classified as fast- or slow-recovering. The first group could be associated with NK and B-cells, whose short lifespan suggests a need for rapid regeneration within weeks, while the second group could consist of long-lived T-cells, which may take years to fully recover [68,[112], [113], [114], [115]]. Alternatively, this classification could be based on regeneration mechanisms: fast-recovering lymphocytes might rely on homeostatic proliferation, whereas slow-recovering ones may primarily depend on the maturation of BM precursor cells in the thymus [[116], [117], [118]]. In addition, RT not only depletes lymphocytes but may also damage the lymphopoietic system, including the BM, thymus, spleen, and lymph nodes (LN), depending on the target area irradiated. This damage can impair lymphopoiesis, resulting in a prolonged inability to return to pre-treatment ALC levels [89,114]. It is possible that both hypotheses contribute to the observed chronic RIL post-treatment, with each playing a specific role in the recovery dynamics.

Although impacting factors could be elucidated, the mechanisms underlying RIL remain largely unclear. Some authors, such as Jin et al. [119] and Yovino et al. [103], attributed RIL primarily to the direct irradiation of blood, leading to the depletion of circulating lymphocytes. With their limited DNA repair capacity, lymphocytes are highly sensitive to radiation, even at low doses [120]. However, the ALC dynamics observed during and after ECIB challenge this assumption. Despite the entire blood volume receiving average doses on the order of hundreds of Gy, the EoT-RLC for ECIB often remained finite around 10–20 %, a range also commonly observed in ICE cases. Likewise, although the lymphopoietic system not being directly irradiated, the RLC still recovered to only 40–60 % of the initial level and remained constant for up to a year, such as in Weeke's study [93,[95], [96], [97]].

A plausible explanation for this paradox is that only a small percentage of lymphocytes reside in the blood; the majority are located in the lymphatic system and organs such as the LN, spleen, BM, and thymus [[121], [122]]. There is continuous exchange of lymphocytes between blood and lymphatic tissues [[123], [124], [125], [126], [127], [128], [129], [130]]. Therefore, even after significant lymphocyte depletion from high-dose irradiation, blood lymphocyte levels are quickly restored—within a few hours or days—by lymphocyte trafficking from the lymphatic system, which holds the majority of the body’s lymphocytes. This hypothesis was supported by Cottier et al., [131] demonstrating that ECIB in calves not only led to a new lymphocyte count level but impacted lymphoid tissues such as the thymic cortex and lymph follicles. Thus, the primary cause of acute RIL during RT appears to be the irradiation of both blood and lymphocyte-rich compartments.

As shown in Fig. 1, patients with a faster decline in RLC during RT tended to exhibit a faster recovery rate post-treatment. This trend could be explained by an intrinsic feedback mechanism that stimulates lymphocyte production when lymphatic system damage or lymphocyte depletion exceeds a certain threshold. However, further analysis is needed to confirm the existence of such a mechanism and to assess its characteristics.

### Biological aspects impacting RIL dynamics

The high variability in ALC nadir values across different cancer types and anatomical sites indicates the differential importance of specific organs and body compartments in the development of RIL. The highest EoT-RLC was observed in brain tumors and breast cancer patients, likely due to the relatively small PTV and therefore mean body dose, and —in the case of brain tumors— the limited presence of lymphocyte-containing tissue. Conversely, patients with tumors in the esophagus, head and neck, pelvis and lung regions exhibited the lowest value EoT-RLC values of 20–30 %, probably attributable to larger irradiated body volumes and abundance of highly perfused organs and lymphocyte-rich tissues in the irradiated regions, such as LNs, or BM, which plays a central role in lymphopoiesis as the main source of hematopoietic stem cells and is particularly vulnerable to radiation exposure [89,[132], [133]].

Esophageal and lung cancers, particularly NSCLC, were associated with significantly low EoT-RLC (∼20 %), identifying the chest region as a high-risk area for RIL. Several mechanisms may explain this phenomenon. Some studies highlighted the impact of regional LN irradiation [6,72] or thymic irradiation [[134], [135]], while others suggested the involvement of BM irradiation in the thoracic spine and ribs [136], which contain approximately 20 % and 8 % of the body’s BM, respectively [137]. Furthermore, the high blood flow through this region—encompassing the lungs, aorta, and heart—combined with radiation exposure, likely contributes to severe lymphocyte depletion.

Pancreatic cancer exhibited intermediate EoT-RLC values (∼34 %), which, in addition to the factors mentioned above, may also be influenced by incidental splenic irradiation. The spleen is a major lymphoid organ involved in lymphocyte recirculation, and previous studies have reported correlations between splenic dose and RIL severity in patients with pancreatic and other abdominal cancers [87,138].

Several studies in the present analysis included patient groups with varying treatment regimens, some of which involved LN irradiation [68,75,82,[88], [89]]. Surprisingly, these studies did not distinguish their ALC results between patients who received LN irradiation and those who did not. Tumor-draining LNs are crucial for initiating T-cell priming against tumor antigens [139]. There is substantial evidence of their pivotal role in immune reaction, particularly in the context of immunotherapy. LN deficiency, the blocking of T-cell egress, irradiation of LN and dissection of lymphatic ducts all promote tumor growth [[140], [141], [142], [143], [144], [145]].

### Impact of radiation quality on RIL dynamics

The type of radiation can impact the incidence and severity of RIL. Protons and carbon ions are expected to be safer for lymphocytes due to the reduced irradiated body volume and lower integral body dose required to deliver the same biological dose to the PTV. This dose to body tissues and lymphocyte-rich organs aligns with the observed correlation between ion therapy and reduced RIL severity [[33], [34],^36^,[54], [55],^57^,^59^,^61^,^70^,^73^]. Our analysis comparing ALC dynamics for photons versus ion therapy further supports this trend: EoT-RLC values were consistently lower in patients receiving photon therapy than in those treated with ions, with an average absolute difference of 11 % and a relative increase of 63 % in EoT-RLC for ion therapy.

For similar reasons, SBRT has also been associated with reduced lymphocyte depletion, lowering the risk of acute severe lymphopenia and correlating with better clinical outcomes [21,69,85]. However, Ellsworth et al. [79] reported a more severe fractional lymphocyte loss with SBRT compared to conventional fractionated RT (CFRT), likely due to the higher dose per fraction typically delivered in SBRT. Park et al. [45] found no significant correlation between SBRT and OS, suggesting that the impact of SBRT on lymphocyte preservation may not consistently translate into survival benefits across all studies and patient populations.

Delivering RT in combination with immunotherapy has become an established strategy for enhancing tumor control through immune system activation [[146], [147]]. However, the success of such combined approaches depends critically on maintaining immune competence during treatment. Understanding RIL is therefore essential for fully exploiting the therapeutic potential of immunotherapy. Minimizing RIL not only supports effective local tumor control but also sustains long-term immune surveillance and facilitates systemic immune responses, including potential abscopal effects, where immune activation in irradiated sites contributes to the regression of distant lesions [148]. By reducing unnecessary irradiation of lymphocyte-rich tissues, proton and carbon ion therapies may further preserve immune function and help realize the full benefits of radioimmunotherapy. Also, an enhanced relative biological effectiveness is expected to lead to more cell inactivation per Gy, initiating a stronger immune response via damage-associated molecular patterns (DAMP) signaling [149].

### Limitations of the analysis

This analysis was limited to ALC data from patient cohorts, as individual ALC curves often showed large fluctuations, were based on small sample sizes (at most 15 patients per paper), and lacked sufficient clinical or dosimetric detail. As a result, ALC values represent mean or median levels of patient groups, and intragroup analyses of specific factors such as PTV size, dosimetric quantities, fractionation, and others were not feasible. Comparisons between cohorts were also challenging, as they differed in multiple parameters—cancer site, target dose, fractionation, radiation modality, PTV, and others—while many cancer sites were represented by only a few datasets. Heterogeneity in study design and reporting further limited the ability to draw definitive conclusions about functional dependencies. Therefore, conclusions on the influence of factors such as PTV, target dose, chemotherapy, fractionation scheme, irradiation technique, age, and sex on ALC dynamics were derived primarily from the texts of the reviewed publications rather than from direct analysis of ALC data. Nevertheless, the overall review revealed consistent trends and highlights key factors likely to influence RIL dynamics and severity. These factors are in particular the type of cancer and the radiation quality, which is why they have been discussed in more detail across all studies in the present analysis. Despite substantial heterogeneity among studies, clear trends could be identified and, in some cases, quantified.

## Conclusion

This study introduces a comprehensive database for ALC dynamics, providing a foundation for analyzing the mechanisms and clinical implications of RIL. Using this database, we identified key patterns, including the potential influence of anatomical site, treatment modality, and pretreatment conditions on lymphocyte depletion and recovery. These findings highlight the complex interplay between treatment modalities, dosimetric factors, and patient-specific characteristics in the development and severity of RIL. Advanced RT techniques may potentially mitigate lymphopenia, warranting further investigation.

This database paves the way for qualitative and quantitative model-aided studies. Such approaches will be crucial for improving lymphopenia prediction, enabling more effective strategies to better preserve immune function and improve therapeutic outcomes by considering biological, dosimetric, and patient-specific factors in treatment planning.

## CRediT authorship contribution statement

**Vladislav Sandul:** Formal analysis, Investigation, Resources, Methodology, Data curation, Writing – original draft. **Sarah Salih Al-Hamami:** Investigation, Resources, Writing – original draft, Writing – review & editing. **Jiří Kubeš:** Supervision, Writing – review & editing. **Marco Durante:** Supervision, Writing – review & editing. **Thomas Friedrich:** Conceptualization, Supervision, Investigation, Writing – review & editing.
