# Supplementary material for: Radiation-induced lymphopenia: A data compilation to unveil relevant factors and mitigation strategies
Source: Clin Transl Radiat Oncol. 2025 Nov 10;56:101071. doi: 10.1016/j.ctro.2025.101071 (PMC12662089; doi:10.1016/j.ctro.2025.101071)
Supplement: Supplementary Data 1 [file mmc1.docx]

**Supplementary materials**

**1. Detailed summary of the reviewed studies: ICE and ECIB modalities**

**Table A1.** Summary of publications investigating radiation-induced lymphopenia (RIL). Included are both studies incorporated into the database and additional publications excluded due to challenges in digitizing ALC dynamics from available plots. For each publication, the following are reported: cancer type and irradiated site (in parentheses), radiation modality (specified where possible; denoted as "RT" if not further detailed; sub-cohorts are shown separately when specific data are available, otherwise grouped by "and" or "or"), use of chemotherapy (CT) (marked "Yes" if used for at least part of the patients, "No" if not used, and left blank if not reported), the total dose to the target (noting whether values are median, mean, range, or IQR; RBE-weighted doses for ion therapies), fractionation scheme (as number of fractions, dose per fraction or time of RT), number of patients (partitioned by modality when possible), estimated end-of-treatment relative lymphocyte count (EoT-RLC), RLC recovery (i.e., lymphocyte levels at follow-up time points post-RT), and key findings regarding lymphopenia or lymphocyte-related outcomes. A list of corresponding abbreviations is provided below the table A2.

| **Publication** | **Cancer type (irradiated organ / site)** | **Radiation modality** | **CT usage** | **Total dose to target (Gy)** | **Fractionation** | **N patients** | **Estimated EoT-RLC, %** | **RLC recovery, %** | **Conclusions** |
| --- | --- | --- | --- | --- | --- | --- | --- | --- | --- |
| **Publications included into the database** | | | | | | | | | |
| Lee et al., 2020 ^44^ | Anal SCC (Anal) | Photons | Yes | Median [IQR]: 54.0 [50.4–54.0]; Elective nodes 45.0 [42.0–45.0] | Median [IQR]: 30 [28–30] Fx | 127 | 24 | 43 after 1 year | - G4 TRL at 2 months linked to higher death risk - No significant OS association for baseline ALC, WBC, platelets count, or 2-month ALC, WBC, platelets count - G4 TRL at 2 months shows a 3.7-fold increased risk of death |
|  |  | Protons |  |  |  | 13 |  |  |  |
| Park et al., 2019 ^45^ | Bone metastasis from HCC (Bones in various sites) | SBRT | Yes | BED10 Median [range]: 50.7 [18.8 - 150] | 5-20 Fx | 26 | 53 | 76 after 1 year | - TRL linked to worse OS - Baseline ALC and TRL correlated with OS, with TRL as an independent predictor - Key TRL factors: post-RT therapy, concurrent CT, hypersplenism, lesion count, RT field size, critical structures, and active BM%. - Only active BM% was an independent TRL predictor. |
|  |  | Non-SBRT |  |  |  | 276 |  |  |  |
| Grossman et al., 2011 ^12^ | Glioblastoma (Brain) | RT | Yes | - | - | 96 | 50 | 50 after 1 year | - Key prognostic factors: histology, extent of surgery, age, baseline and post-RT CD4 counts - Hazard ratio increased with CD4 count toxicity severity (1.7 per unit increase) |
| Kim et al., 2023 ^46^ | Glioblastoma (Brain) | 3D-CRT | Yes | 60 | 30 Fx | 61 | 73.8 | - | - Hypofractionated RT had similar oncologic outcomes with reduced ALC drop - RIL G2+ predictors: baseline ALC, PTV, sex - Severe ALC drop at 6 months is linked to worse outcomes; Hypofractionated RT mitigated this decline |
|  |  | IMRT |  | 60 | 30 Fx | 84 |  |  |  |
|  |  | IMRT |  | 58.5 | 25 Fx | 78 | 78.3 |  |  |
| Mendez et al., 2016 ^47^ | Glioblastoma (Brain) | RT | Yes | 70% of pat. got more than 45 | - | 72 | 73 | 82 after 1 year | - Severe lymphopenia linked to shorter median OS & higher mortality - Better prognosis with MGMT methylation, gross total resection, and RT >45 Gy |
| Mohan et al., 2021 ^36^ | Glioblastoma (Brain) | Protons (PSPT and IMPT) | Yes | 50-60 | 30 Fx | 28 | 54 | 89 after 4 weeks | - Sex, baseline ALC, whole-brain V20: strong predictors of G3+ lymphopenia in GBM with CRT - Proton therapy reduced low/intermediate brain dose & G3+ lymphopenia incidence |
|  |  | Photons (VMAT and IMRT) |  |  |  | 56 | 68 | 80 after 4 weeks |  |
| Rudra et al., 2018 ^48^ | Glioblastoma (Brain) | 3DCRT | Yes | 60 | 30 Fx | 28 | 52 for SFRT 56 for LFRT | 58 for SFRT and 67 for LFRT after 6 weeks | - LFRT linked to larger tumor volume but lower brain dose metrics & reduced lymphopenia - Brain V25 Gy was a key ASL predictor - G3+ lymphopenia correlated with worse survival |
|  |  | IMRT |  |  |  | 182 |  |  |  |
| Chen et al., 2021 ^49^ | Breast cancer (Breast) | RapidArc | Yes | Median [range]: 40.5 [40.5-60] | Median [range]: 15 [15-25] Fx | 123 | 60 | 90 after 1-3 months | - RT technique (RapidArc), mean lung dose, and CT: key lymphopenia risk factors - RT technique was the only significant predictor of nadir-PLC/pre-PLC <0.8 - RapidArc led to greater PLC reduction & higher lung V5, despite matched mean lung dose & volume |
|  |  | 2D-fields |  |  |  | 277 |  |  |  |
|  |  | 3DCRT |  |  |  | 335 |  |  |  |
| Standish et al., 2008 ^50^ | Breast cancer (Breast) | RT | Yes | 60-65 | 30-36 days | 14 | 61 | 84 after 6 weeks | - Chest RT caused lymphopenia, low NK cell activity, reduced monocyte phagocytosis, and decreased TNF-α - Lymphopenia and NK cell suppression were linked to RT area, not dose |
| Wu et al., 2016 ^51^ | Cervical cancer (Cervix) | RT | Yes | - | - | 71 | 20 | 33 after 1 year | - OS was significantly lower for patients with pre-treatment and post-treatment lymphopenia |
| Yang et al., 2022 ^52^ | Cervical cancer (Cervix) | 3D-CRT and BRT or RapidArc and BRT | Yes | 80-85 | 1.8-2.3 Gy/Fx | 104 | 31 | - | - MBD, concurrent cisplatin, and pre-RT ALC were linked to EOSL - EOSL patients had worse OS and PFS |
| Davuluri et al., 2017 ^34^ | Esophageal Cancer (Esophagus) | IMRT | Yes | - | - | 317 | 22 | - | - G4 nadir was linked to worse OS, PFS, LRFS, DMFS, and DSS - Predictors: distal tumor, definitive CRT, taxane/5-FU CT, photon RT - Photon RT correlated with higher MBD, a strong G4 nadir predictor (OR 1.22/Gy) - MVA identified G4 nadir, poor differentiation, and stage III as OS risk factors; surgery improved OS - PBT significantly reduced G4 nadir vs. IMRT, especially in weeks 4-5 of CRT, due to lower MBD - PBT’s OS benefit was not significant after G4 nadir adjustment, suggesting its main advantage is lymphocyte sparing |
|  |  | Protons |  |  |  | 187 | 28 |  |  |
| Deng et al., 2019 ^53^ | Esophageal Cancer (Esophagus) | IMRT | Yes | Median [range]: 50.4 [41.4–66.0] | 5 weeks | 511 | 20 | 59 after 7 weeks | - G4 lymphopenia during CRT predicted worse OS vs. G0-3 - Lymphocyte recovery post-CRT did not improve OS; recovered G4 patients had lower 5-year OS than non-recovered G0-3 - In G4 patients, recovery was linked only to baseline ALC, unaffected by treatment grade - OS, PFS, DSS, LRFS, and DMFS were worse for G4 vs. G0-3 - Post-CRT/pre-CRT ALC ratios were similar for G0-3 and G4, showing recovery was independent of lymphopenia grade - PBT maintained higher ALC during CRT and improved OS vs. IMRT, despite similar pre- and post-CRT ALC |
|  |  | Proton |  |  |  | 244 |  |  |  |
| Durante, 2024 ^54^ | Esophageal Cancer (Esophagus) | IMRT | No | 60-70 | 1.6-2.0 Gy/f x | 15 | 14 | - | - Particle therapy significantly reduces RT-induced lymphopenia across tumor sites - It may be the optimal choice for combined RT and immunotherapy |
|  |  | CIRT |  |  | 2.7-3.6 Gy/Fx | 4 | 33 |  |  |
| Ebrahimi et al., 2021 ^55^ | Esophageal Cancer (Esophagus) | IMRT | Yes | 50.4 | 28 Fx | 15 | 7-40 | - | - Proton plans reduced post-RT lymphopenia risk vs. photons - IMPT provided better lymphocyte preservation than PSPT |
|  |  | PSPT |  |  |  | 15 |  |  |  |
|  |  | IMPT |  |  |  | 15 |  |  |  |
| Ebrahimi et al., 2022 ^56^ | Esophageal adenocarcinoma and SCC  (Esophagus) | Protons | Yes | 50.4 | 5 weeks | 860 | 5-22 | - |  |
|  |  | Photons |  |  |  |  |  |  |  |
| Fang et al., 2018 ^57^ | Esophageal Cancer (Esophagus) | IMRT | Yes | Mean: 50 | 5 weeks | 209 | 20 | - | - High ALC predictors: PBT vs. IMRT, smoking, early-stage (I–II), SCC histology - MBD inversely correlated with high ALC nadir - Higher ALC during neoadjuvant CRT linked to increased pathologic complete response rate |
|  |  | Protons |  |  |  | 104 | 24 |  |  |
| Ni et al., 2022 ^58^ | Esophageal cancer (Esophagus) | IMRT | Yes | 54 | 27 Fx | 74 | 24 | 52 after 9 weeks | - G4 lymphopenia had lower 3-year disease-free survival but similar 3-year OS vs. G1-3 - Age, sex, and RT technique were not G4 lymphopenia risk factors - Higher tumor mass, volume of bone, heart, PTV doses, sternum mean dose, V10, V20 correlated with G4 lymphopenia risk |
|  |  | VMAT |  |  |  | 42 |  |  |  |
| Shiraishi et al., 2018 ^33^ | Esophageal Cancer (Esophagus) | IMRT | Yes | Median: 50.4 | 28 Fx | 334 | 19 | - | - PBT reduced G4 lymphopenia risk vs. IMRT (OR 0.29) - G4 lymphopenia predictors: older age, larger PTV, IMRT over PBT - G4 lymphopenia during CRT correlated with worse OS, PFS, and DMFS |
|  |  | Protons |  |  |  | 146 | 25 |  |  |
| Sumiya et al., 2021 ^59^ | Esophageal Cancer (Esophagus) | Protons | Yes | 60-70 | 2 Gy / Fx | 54 | 23 | 85 after 26 weeks | - Dose-volume analysis linked thoracic bone irradiation (5–50 Gy) to lower ALC and higher NLR - PBT maintained significantly higher ALC at 1, 5, and 6 weeks vs. XRT - Patients with ALC ≥200 had better 2-year OS and PFS |
|  |  | Photons |  | 60 |  | 15 | 10 | 66 after 26 weeks |  |
| Wang et al., 2020 ^60^ | Esophageal SCC (Esophagus) | 3D-CRT | Yes | 50-68 | 5 weeks | 66 | 25 | 56 after 4 weeks | - Low ALC nadir during RT was linked to worse OS and PFS - Many patients with low ALC nadir had locoregional or distant progression - High lung/heart radiation (V5, V10) and Log10(PTV) correlated with lower ALC nadir |
|  |  | IMRT |  |  |  | 123 |  |  |  |
| Wang et. al, 2024 ^61^ | Esophageal ​Adenocarcinoma and SCC (Esophagus) | IMRT | Yes | 50.4 | 28 Fx | 61 | 14 | - | - G4 lymphopenia was strongly associated with induction CT, baseline ALC, RT modality, and PTV - PBT reduced G4 lymphopenia risk in patients with intermediate ALC and large PTV vs. IMRT - Baseline ALC <1020 cell/μl led to 100% G4 lymphopenia, while ALC ≥1020 cell/μl with PTV <358 ml had 0% G4 lymphopenia - Lung V5Gy, V20Gy, V30Gy, and mean lung dose ≥10 Gy were key G4 lymphopenia predictors - PBT minimized heart/lung doses vs. IMRT but had higher V30Gy |
|  |  | Protons |  |  |  | 44 | 23 |  |  |
| Xu et. al., 2021 ^62^ | Esophageal Squamous Cell Carcinoma (Esophagus) | 3DCRT | Yes | 50-60 | 25-30 Fx | 115 | 23 | 72 after month | - G4 lymphopenia was linked to larger PTVs, higher lung V10, and heart V10, indicating worse outcomes in definitive CRT - It correlated with worse OS, PFS, and DMFS but not with LRFFS |
|  |  | IMRT |  |  |  | 321 |  |  |  |
| Zhou et al., 2019 ^63^ | Esophageal SCC(Esophagus) | 3D-CRT | Yes | 50–60 | 1.8–2.0 Gy /Fx | 67 | 27 | - | - Pretreatment lymphopenia was associated with larger tumors, worse T status, body mass index ≤18.5 kg/m², and ≥3 kg weight loss in 3 months - TRL predictors: older age, lower tumor location, larger tumor, larger PTV - CR rate was lower in patients with pretreatment (9.1%) and TRL (11.2%) - Tumor progression and cancer-related death were more frequent in the TRL group, while pretreatment lymphopenia was not significantly linked to these outcomes |
|  |  | IMRT |  |  |  | 219 |  |  |  |
| Campian et al., 2014 ^64^ | Head and neck SCC (Head/Neck) | RT | Yes | - | - | 56 | 25 | 54 after 1 year | - HPV-negative patients with lymphocytes < 500 cells/mm³ at 2 months had faster disease progression (HR 5.75) - Multivariate analysis confirmed higher progression risk - No significant univariate associations with PFS |
| Liu et al., 2018 ^65^ | Nasopharyngeal carcinoma (head/neck) | IMRT | Yes | 68-70 | 30-Fx | 413 | 15 | 53 after 13 weeks | - Lymphopenia elevated the risk of death, disease progression, and distant metastasis - ALC was an independent prognostic factor for OS and PFS |
| Ng et al., 2020 ^66^ | Oropharyngeal cancer (head/neck) | RT | Yes | Median [range]:70 [50-73] | Median [range]: 33 [28-42] Fx | 851 | 18 | 44 after 9 weeks | - No significant OS or disease control differences between patients with and without G3/G4 lymphopenia - Lowest ALC during treatment did not impact survival - No correlation between induction CT and G3/G4 lymphopenia - Concurrent CT inversely associated with G3/G4 lymphopenia - OS factors: age at diagnosis, smoking status |
| Xie et al., 2020 ^67^ | Nasopharyngeal carcinoma (head/neck) | 2D-CRT | Yes | Median [range]: 72.7 [68-82] | 40 Fx | 261 | 30 | - | - Lymphopenia during RT was influenced by baseline ALC and RT modality - G3–4 ALC nadir correlated with longer PFS and LRFS - G4 ALC nadir was linked to shorter DMFS |
|  |  | IMRT |  |  |  | 113 |  |  |  |
| Heier et al., 1975 ^68^ | Hodgkin’s disease and seminoma testis (Pelvis) | Photons | No | 40 | 20 Fx | 47 | 24 | 46 after 6 months;  ~ 96 after 10 years | - B-cell depletion was more pronounced than T-cell, indicating higher radiosensitivity - T-cell recovery was slower, with full restoration taking 10 years |
| Byun et al., 2019 ^69^ | HCC (Liver) | 3D-CRT | Yes | 45-60 | 20-25 Fx | 515 | 27 | 73 after 1 year | - ASL independently predicted worse OS - Key risk factors: larger PTV, lower baseline ALC, hepatic arterial infusion CT - Hypo-fractionated SBRT significantly reduced ASL risk - Other OS predictors: tumor count, Child–Pugh class, treatment history, BED, combined hepatic arterial infusion CT, PTV, baseline NLR |
|  |  | IMRT |  | 45-60 | 20-25 Fx | 90 |  |  |  |
|  |  |  |  | 52 or 60 | 4 Fx | 315 |  |  |  |
| De et al., 2021 ^70^ | Unresectable HCC (Liver) | Photons (IMRT and VMAT) | Yes | Median [range]: 60 [35–100] | Median [range]: 15 [3–34] | 73 | 26 | 54 after 7 weeks | - G3 and G4 lymphopenia may worsen outcomes in HCC patients receiving RT - PBT may mitigate lymphopenia vs. photons by reducing lymphopoiesis site exposure |
|  |  | Protons |  | Median [range]: 68 [30–100] |  | 35 |  |  |  |
| Kim et al., 2024 ^71^ | HCC (Liver) | Tomotherapy or VMAT | Yes | Integral body dose, Median [range]: 104 [14-407] | Median [range]: 2.2 [1.5-6] Gy/Fx | 75 | 16 | - | - Mean blood dose strongly correlated with ALC depletion (r = 0.664) - Pre-RT ALC and fraction number were lower in the severe RIL group - Severe RIL group received higher integral body and mean blood doses - No significant differences in sex, age, dose per fraction, beam time, GTV, PTV, tumor stage, CT, or RT modality between severe RIL and non-severe RIL groups |
| Campian et al., 2013 ^11^ | NSCLC (Lung) | RT | Yes | Median [range]: 60 [43.2–70.2] | - | 47 | ~ 25 | 40-50 after 6 months | - MVA linked severe ALC to survival (HR 1.70) - ALC <500 cells/mm³ at 2 months post-CRT increased death risk by 70%, adjusting for RT dose, age, and neoadjuvant CT - No significant univariate associations with OS; CT regimens had no impact on ALC or survival |
| Cho et al., 2022 ^72^ | NSCLC (Lung) | RT | Yes | 60 | 30 Fx | 66 | 20 | 58 after 13 weeks | - Lymphopenia recovery strongly correlated with improved survival in CCRT + adjuvant immunotherapy - Pre- and post-CCRT ALC were linked to recovery - Voxel-based analysis showed a significant association between large vessel dose and lymphopenia at CCRT end - Focus should be on both preventing lymphocyte depletion and promoting recovery post-CCRT |
| Kim et al., 2021 ^73^ | NSCLC (Lung) | IMRT | Yes | 66 | 30 Fx | 194 | 11 | - | - PBSPT reduced irradiated lung volumes, lowering severe SRL.  Lung V5Gy contributed to SRL - Optimized RT to minimize SRL may improve outcomes |
|  |  | PBSPT |  |  |  | 29 | 17 |  |  |
| Nowicka et al., 2024 ^74^ | NSCLC (Lung) | IMRT or VMAT | Yes | Median [range]: 60 [60–66] | Median [range]: 30 [30-33] Fx | 306 | 21 with and 23 without CT | - | - ALC loss (11–78%) correlated with dose-volume metrics, especially lung V20–30 Gy in IMRT - Median ALC loss over 45 days was 52.2%, higher in CRT vs. RT alone - Severe RIL was more frequent in IMRT (51%) vs. VMAT (37%) due to higher lung/heart dose - %ALC loss correlated more with DVH metrics than lymphocyte nadir—lung V15–V35 for IMRT, heart V25–V35 for VMAT - MVA showed baseline ALC, CRT use, and PC1 predicted %ALC loss better than clinical factors and EDRIC (R² = 0.40 vs. 0.39) |
| van Rossum et al., 2023 ^75^ | NSCLC (Lung) | IMPT | Yes | Median: 70  Involved lymph nodes: 60 | 24 Fx | 100 | 18 | - | - ALC showed an overall declining trend, reaching nadir around day 30 (median treatment duration: 32 days) - G3+ lymphopenia occurred in 78% of patients; G4 in 17% - Lower baseline ALC and higher PTV were significant predictors for both G3+ and G4 lymphopenia - Right-sided tumors and higher mean lung dose correlated with G3+ lymphopenia |
| Balmanoukian et al., 2012 ^10^ | Pancreatic adenocarcinoma (Pancreas) | RT | Yes | Median [range]  50.4 [27–57.6] | 1.8 Gy / Fx | 53 | 38 | ~40 after 14 months | - Adjuvant CRT frequently caused severe lymphopenia - Lymphopenia was an independent survival predictor - CT regimens had no significant impact on ALC or survival - Survival was significantly associated with ALC at 2 months, adjusting for baseline nodal status and lymph count normality |
| Heo et al., 2022 ^76^ | Pancreatic adenocarcinoma (Pancreas) | RT | Yes | 45-54 | 25-27 Fx | 68 | 48 | 46 after 14 weeks | - Initial ALC significantly impacted LRFS, DMFS, and OS - Patients with ALC ≥1540 × 10⁶/l had better 3-year OS and LRFS, but DMFS showed no significant difference |
| Jin et al., 2020 ^42^ | Pancreatic adenocarcinoma / cholangiocarcinoma / ampullary cancer (Pancreas) | RT | Yes | Median [range]: 54 [45–57.5] | 5 weeks | 51 | 7-68 | - | - Median radiosensitivity: 0.40 Gy⁻¹, reproductivity: 0.3% day⁻¹ |
| Wild et al., 2015 ^77^ | LAPC (Pancreas) | IMRT | Yes | Median [IQR]  50.4 [50–50.4] | Median [IQR]:  1.8 [1.8–2] Gy / Fx | 44 | 26 | ~ 40 after 1 year | - Key survival predictors: baseline albumin, ALC at 2 months, blood urea nitrogen, platelet count, and larger PTV - CT type had no significant impact on ALC or survival |
|  |  | 3D-CRT |  |  |  | 57 |  |  |  |
| Yang et al., 2024 ^78^ | LAPC (Pancreas) | Photons | Yes | 45-60 | 12 Fx | 337 | 34 | 60 after 47 weeks | - Severe RIL predictors: higher baseline ALC, CIRT modality - Recovery from severe RIL linked to CT regimen and PTV - Median OS was longer with CIRT vs. photon RT. - Independent OS/PFS prognostic factors: male sex, tumor size, RT modality, post-RT surgery - RT modality and RIL were significantly associated with OS |
|  |  | CIRT |  | 48-55.2 | 25 Fx | 497 | 57 | 70 after 47 weeks |  |
| Ellsworth et al., 2019 ^79^ | Several cancer types (Abdomen, pelvis, TBI) | TBI | Yes | 9.2 | 8 Fx | 30 | 2 | - | - FLL correlations varied by site and included field size, dose/Fx, mean spleen dose, CT backbone, and age - Total ALC loss during RT was strongly linked to FLL (p < 0.001) - Lymphocyte depletion during fractionated RT followed exponential decay - Mean FLL: TBI 35.5%, SBRT 24.3%, CFRT 10.77% - Highest FLL occurred in patients without concurrent CT, suggesting ALC loss was RT-driven |
|  |  | SBRT |  |  | 3-5 Fx | 73 |  |  |  |
|  |  | RT |  | 45 | 25 | 316 | 15 |  |  |
| Cozzarini et al., 2016 ^80^ | Prostate Cancer (Pelvis) | IMRT | No | 70.4  Lymph-nodal dose: 50.2 | Median: 2.35 Gy / Fx | 19 | 30 | 54 after 1 year | - Baseline ALC predicted acute G3+ lymphopenia - Hypofractionation, tumor stage, and EQD2 to lymph nodal volume were key predictors of acute neutropenia and thrombocytopenia - IMRT technique had no impact on lymphopenia risk - Smoking ≥1 year significantly increased the risk of G2+ lymphopenia |
|  |  | HTT |  |  |  | 46 |  |  |  |
|  |  | VMAT |  |  |  | 60 |  |  |  |
| Schad et al., 2019 ^81^ | Prostate Cancer (Pelvis) | 3DCRT and BRT or IMRT and BRT | No | Median [range]: 46 [44-56] for treated PN;  65 [25-79.5] for untreated PN | 22-25 Fx | 886 | 46 for treated LN, 73 for untreated LN | 93 after 5-6 years | - Key RIL predictors: initial PSA, baseline lymphopenia, RT modality, pelvic nodal irradiation status, larger PTV, androgen deprivation therapy - Pelvic nodal irradiation (HR 3.42), baseline lymphopenia (HR 8.32), and PSA (HR 1.05) were strongest RIL predictors - RIL showed no association with biochemical PFS, DMFS, or OS due to limited events |
| Xiang et al., 2022 ^82^ | Pelvic malignancies (Pelvis) | VMAT | No | Prostate cancer: 67.5 | 25 Fx | 188 | 24 | 55 after 6 months | - Multiple regression identified basal PLC, sex, and total pelvic bone V5 as predictors of final ALC, with basal PLC having the strongest impact (29.7% variability) - Reducing pelvic bone radiation may help prevent severe lymphocytopenia |
|  |  |  |  | Gynecologic malignancies: 45–50.4 | 25-28 Fx |  |  |  |  |
| Ku et al., 2024 ^83^ | Soft tissue sarcoma (Soft tissue) | 3D-CRT | Yes | Median [range]: 52 [39-78] | 1.8-2 Gy / Fx | 22 | 36 | 78 after 48 weeks | - Higher body V10Gy and bone V10Gy correlated with lower post-RT ALC nadir, indicating lymphocyte radiosensitivity - Minimizing treatment-related hematopoietic toxicity may improve outcomes - RT impact on BM lymphocyte progenitors exceeded its effect on circulating lymphocytes in cases with limited heart/lung exposure. - Severe lymphopenia was linked to worse OS |
|  |  | IMRT |  |  |  | 47 |  |  |  |
| **Publications not included into the database** | | | | | | | | | |
| Dixon-Douglas et al., 2024 ^84^ | Breast cancer (Breast) | RT | Yes | - | - | 200 | 56 | 87 after 1 year | - Younger and middle-aged patients had prolonged low ALC; older patients showed no clear trend - Dose-dense CT and specific regimens caused deeper, longer lymphopenia vs. standard schedules - Adjuvant RT further reduced ALC, while non-RT patients showed slight recovery - Flow cytometry showed a shift from central to effector memory CD4+ cells, mainly in premenopausal patients - Recent thymic emigrants increased post-CT, suggesting thymic recovery in this group |
| Dai et al., 2022 ^19^ | Esophageal Cancer (Esophagus) | IMRT, 3D-CRT or  Protons | Yes | 50.4 | 1.8–2 Gy /Fx | - | Mean: 20 | ~60 after 6 weeks | - Severe RIL was associated with lower pathologic complete response rates, worse OS, and poorer PFS in esophageal cancer - Key dosimetric predictors: larger PTV, higher heart/body doses, increased EDIC - Clinical risk factors: lower baseline ALC, greater tumor length, advanced stage, distal tumor location |
| Durante et al., 2020 ^38^ | Esophageal Cancer (Esophagus) | IMRT |  | 45 | 25 Fx | - | 20 | - | - PBT may reduce severe lymphopenia risk, especially in esophageal cancer - Both PBT and CIRT showed less lymphocyte decline vs. X-rays |
|  |  | 3DCRT |  | 60 | 30-38 Fx |  | 10 |  |  |
|  |  | Protons |  | 45 | 25 Fx |  | 26 |  |  |
|  |  | CIRT |  | 65 | 18-24 Fx |  | 53 |  |  |
| Sung et al., 2020 ^43^ | HCC (Liver) | PSPT | No | Median [range]: 58 [45-67.5] | 15 Fx | 17 | 20-85 | ~ 80 in 250 days |  |
| Zhang et al., 2019 ^85^ | HCC (Liver) | RT | Yes | 50.0–84.0 | 1.8–2.5 Gy  / Fx | 68 | 32 | 57 after 12-16 weeks | - In HCC patients, lower lymphocyte nadirs during RT were linked to worse OS - Smaller GTV (≤55 cc) and fewer RT fractions (≤16) correlated with higher ALC and better survival - Lymphocyte nadir and BCLC stage independently predicted OS, with higher nadirs linked to better survival - SBRT improved survival across BCLC stages - Negative correlations were found between GTV and lymphocyte nadir, with no significant link to pre-RT ALC |
|  |  |  |  |  |  |  |  |  |  |
|  |  |  |  |  |  | 58 |  |  |  |
|  |  |  |  | 58.5–91.1 | 2.5–6 Gy / Fx |  |  |  |  |
|  |  |  |  |  |  |  |  |  |  |
|  |  | SBRT |  |  |  | 58 |  |  |  |
|  |  |  |  | 79.0–119.0 | 6.0–10.0 Gy / Fx |  |  |  |  |
|  |  |  |  |  |  |  |  |  |  |
|  |  |  |  |  |  |  |  |  |  |
| Cho et al., 2016 ^86^ | Small cell lung cancer (Lung) | RT | Yes | 54–64 | 30–32 Fx  (1 Fx/day) | 17 | ~10-50 | - | - Both minimum and post-RT ALC levels predicted OS (HR 2.67 for min ALC ≤297 cells/μl, OS: 12.2 vs. 35.3 months; HR 2.62 for post ALC ≤698 cells/μl, OS: 19.3 vs. 46.9 months) - Lymphopenia group (min ALC ≤ 297 or post ALC ≤ 698 cells/μl) had median OS of 19.0 vs. 131.7 months and PFS of 8.1 vs. 16.6 months - Min ALC and post ALC also predicted PFS; min ALC ≤297 cells/μL had PFS of 6.7 vs. 12.6 months, and post ALC ≤698 cells/μl had PFS of 9.3 vs. 13.3 months - Age and NLR impacted OS and PFS in univariate analysis; pre-RT ALC and CT cycle were relevant only for OS - Lactate dehydrogenase >160 U/I and ECOG 2 trends toward poorer OS; sex, sequential CRT, QD/BID RT were not significant - PCI was linked to lower lymphopenia risk; age >70 and fewer than 4 CT cycles were non-significant risks for lymphopenia - GTV correlated with min ALC |
|  |  |  |  | 45–51 | 30–36 Fx  (2 Fx/day) | 56 |  |  |  |
| Upadhyay et al., 2021 ^21^ | Thoracic malignancies (Lung) | Various modalities | Yes | 45-60 | various | - | Mean [range]: 42 [26-50] | - | - Severe lymphopenia increased death risk (HR 1.59) and progression risk (HR 2.1). Key factors: advanced age, large tumor size, high cancer stage, low baseline ALC, and certain dosimetric parameters (e.g., GTV, lung V5, heart V5) - Hypofractionated RT (<4 weeks) reduced severe lymphopenia risk by 71%, while hyperfractionated RT increased risk in limited-stage SCLC - SBRT showed less ALC decline than standard RT, linked to better outcomes - Higher EDIC and EDRIC values correlated with lower ALC, more G3 lymphopenia, and shorter OS/PFS |
| Chadha et al., 2017 ^87^ | LAPC (Pancreas) | IMRT or  3D-CRT | Yes | Median [range]: 50.4 [25.2-70.4] | 28 Fx | 177 | 43 | - | - Post-CRT ALC <500 cell/µl was linked to worse OS in both univariate and multivariate analysis - DVH thresholds at V5, V10, V15, and V20, along with MSD, were significant for severe lymphopenia, confirming dose-dependent lymphocyte decline post-CRT |
| Xu et al., 2022 ^88^ | Cervical carcinoma (Pelvis) | 3D-CRT | Yes | 45-57.5 | 25 Fx | 27 | 12 | 72 after 4 weeks | - ECOG performance status, pre-RT ALC, and pre-RT hemoglobin levels were protective against G4 ALC nadir - Concurrent CT promoted G4 ALC nadir - Body volume protected against G4 ALC nadir among DVH parameters - GTV_N volume and PTV_5500 Dmax increased G4 ALC nadir risk among DVH parameters - G4 ALC nadir was linked to poor OS |
|  |  | RapidArc |  | 55-61 | 30-33 Fx | 103 |  |  |  |
| Sini et al., 2016 ^89^ | Prostate cancer (Pelvis) | IMRT | No | 70.4 | Median: 2.35 Gy/Fx | 19 | 30 | 54 after 45 weeks | - Lower baseline WBC, neutrophils, and ALC predicted acute and late lymphopenia - Higher BM V40 increased the risk of acute G3 and late G2 lymphopenia - Specific pelvic BM regions were identified as risk factors for acute/late lymphopenia, raising susceptibility to viral infections |
|  |  |  |  |  |  |  |  |  |  |
|  |  | HTT |  |  |  | 46 |  |  |  |
|  |  |  |  |  |  |  |  |  |  |
|  |  | VMAT |  |  |  | 60 |  |  |  |
|  |  |  |  |  |  |  |  |  |  |
| Grossman et al., 2015 ^7^ | Malignant glioma, NSCLC, pancreatic cancer, (Brain / Pancreas / Lung) | Photons with  BRT | Yes | 43-70 | - | 297 | >=30 | - | - 40% of patients developed severe treatment-related lymphopenia two months after starting CRT, regardless of histology or CT regimen - Severe lymphopenia was independently linked to shorter survival from tumor progression - No complete recovery was observed within one year |

**Table A2.** Summary of publications on RIL caused by extracorporeal irradiation of blood (ECIB). This table includes both studies integrated into the database and those excluded due to limitations in digitizing ALC dynamics. Reported information includes: publication reference, indication for ECIB, mean total dose to blood, transit dose (if specified), fractionation scheme, time of one fraction (if provided), number of patients, estimated EoT-RLC, RLC recovery, and conclusions summarizing key observations on RIL during and after ECIB.

| **Publication** | **Reason of treatment** | **Mean total dose, Gy** | **Transit Dose, Gy** | **Fractionation scheme** | **Time of one fraction** | **N patients** | **Estimated EoT-RLC, %** | **RLC recovery, %** | **Conclusions** |
| --- | --- | --- | --- | --- | --- | --- | --- | --- | --- |
| **Publications included into the database** | | | | | | | | | |
| Chanana 1976 ^90^ | Leukemia |  | 3-8 |  |  | 18 | 5-65 |  | - Lymphocytes exist in multiple body pools with different exchange rates - A rapidly exchanging pool constantly communicates with circulating blood - The rapidly exchanging pool is larger than the circulating compartment - Suggests dynamic lymphocyte trafficking between blood and tissues |
| Cronkite et al., 1971 ^91^ | Chronic  Lymphocytic Leukemia |  |  |  |  |  | 3-63 |  | - ECIB causes lymphopenia - It depletes thymic-dependent lymphoid tissues - Lymph nodes and spleen are less affected - Spleen size and lymph node response vary between patients - Lymphocyte doubling times range from days to months - Some patients maintain low counts before rapid recovery |
| Meuret et al., 1971 ^41^ | Chronic  Lymphocytic Leukemia |  |  |  |  | 6 | 1-19 |  |  |
| Rosengren 1968 ^92^ | Renal transplantation | 90-370 | 5-6 | 8 - 31 days |  | 11 | 33 |  | - Lymphocyte count decreased in 3 of 11 patients - Total WBC count remained stable - The effect of extracorporeal irradiation on transplant rejection remains unclear |
| Weeke et al., 1970 ^93^ | Renal transplantation | 546 | 2.8-6.1 | 3-5 days per week/  treatment period of 10-20 days | 8-10h | 18 | 20 | 37 after 14 weeks | - Lymphocyte levels do not drop to zero despite a significant dose - Within 14 weeks, 10% of lymphocytes recover |
|  |  |  |  |  |  |  |  |  |  |
|  |  |  |  |  |  |  |  |  |  |
| **Publications not included into the database** | | | | | | | | | |
| Andersen et al., 1970 ^94^ | Active Cirrhosis | 272-632 | 3.8-4.95 |  |  | 4 | 20-25 |  | - Post-treatment, lymphocyte concentration dropped to 25% of baseline - Three months later, levels remained below 50% of pre-treatment values - Results resembled those in uremic patients, despite a more intensive treatment schedule - Most recirculating lymphocytes pass through the blood within 3-4 days |
| Weeke et al., 1972 ^95^ | Renal transplantation | 500 | Mean: 3 |  |  | 80 | 25 | 55 after 14 weeks | - Despite receiving a substantial dose, lymphocyte levels do not drop to zero - Lymphocyte recovery from 20% to 50% occurs within 14 months |
| Weeke et al., 1973 ^96^ | Renal transplantation |  |  |  |  | 8 | 30 | ~ 60 after 30 weeks | - Despite a significant dose, lymphocyte levels do not reach zero - Lymphocyte recovery from 20% to 50% occurs within 14 months |
| Weeke et al., 1974 ^97^ | Renal transplantation | 207.5 [99-369] | Median [range]: 1 [0.85-1.1] |  |  | 24 | 2-30 | ~40-60 after 60 weeks | - Despite receiving a significant dose, lymphocyte levels do not reach zero - Recovery from 20% to 50% occurs within 14 months - Lymphocyte levels increase more steeply in the first few months after ECIB - Once a pre-ECIB level of 35-40% is reached, no further increase is observed in the following 5-10 months, regardless of total dose group |
|  |  | 573 [183-851.5] | Median [range]: 37.5 [2.7-5.65] |  |  | 47 |  |  |  |

**ALC** = Absolute lymphocyte count, **ASL** = Acute severe lymphopenia, **BCLC** = Barcelona Clinic Liver Cancer (staging system), **BED** = Biologically effective dose, **BM** = Bone marrow, **BRT** = Brachytherapy, **CCRT** = Concurrent chemoradiotherapy, **CFRT** = Conventional fractionated radiation therapy, **CIRT** = Carbon ion radiation therapy, **CRT** = Chemoradiotherapy, **CT** = Chemotherapy, **DMFS** = Distant-metastasis free survival, **DSS** = Disease-specific survival, **DVH** = Dose-volume histogram, **EDIC** = Effective dose to immune cells, **EDRIC** = Estimated dose of radiation to immune system, **EOSL** = Early onset severe lymphopenia, **ECOG** = Eastern Cooperative Oncology Group, **EQD2** = Equivalent dose in 2 Gy fractions, **Fx** = Fractions, **GTV** = Gross tumor volume, **Gx** = Grade x (severity of lymphopenia, x = 1,2,3 or 4; “+” stand for “or higher”), **HR** = Hazard ratio, **HTT** = Helical Tomotherapy, **IMPT** = Intensity-modulated proton therapy, **IMRT** = Intensity-modulated radiation therapy, **LAPC** = Locally Advanced Pancreatic Cancer, **LFRT** = Limited-field radiation therapy, **LRFS** = Local-regional recurrence-free survival, **MBD** = Mean body dose, **MVA** = Multivariate analysis, **NK cells** = Natural killer cells, **NLR** = Neutrophil-to-lymphocyte ratio, **NSCLC** = Non-small cell lung cancer, **OS** = Overall survival, **PBSPT** = Pencil Beam Scanning Proton Therapy, **PBT** = Proton beam therapy, **PLC** = Peripheral lymphocyte counts, **PSPT** = Passive Scattering Proton Therapy, **PTV** = Planning treatment volume, **PTV_5500 Dmax** = Maximum dose of planning target volume receiving at least 55 Gy, **RBE** = Relative biological effectiveness, **RLC** = Recovery of lymphocytes, **RT** = Radiation therapy, **SBRT** = Stereotactic body radiation therapy, **SCC** = Squamous cell carcinoma, **SFRT** = Standard-field radiation therapy, **TBI** = Total body irradiation, **TNF-α** = Tumor necrosis factor alpha, **TRL** = Treatment-related lymphopenia, **Vx** = Volume of organ receiving at least x Gy (e.g., 5 Gy, 10 Gy, 20 Gy), **VMAT** = Volumetric-modulated arc therapy, **WBC** = White blood cell count.

**2. Procedure for determining therapy duration in radiotherapy**

1. Adoption of explicitly stated therapy duration

If a therapy duration is explicitly stated in the data or publication text (e.g., “therapy lasted 6 weeks”, “25 fx with 5 fx weekly”, etc.), it is used as the duration and subsequent steps are skipped.
*Corresponding publications:* Chanana, 1976 ^90^, Ellsworth et al., 2019 ^79^, Kim et al., 2021 ^73^, Kim et al., 2023 ^46^, Mohan et al., 2021 ^36^, Nowicka et al., 2024 ^74^, Rosengren, 1968 ^92^, Schad et al., 2019 ^81^, Standish et al., 2008 ^50^, van Rossum et al., 2023 ^75^, Wang et al., 2024 ^61^.

2. Calculation based on dose and fractionation

The number of fractions can be calculated from given total dose and fractionation scheme as (total dose)/(dose/fx). From the fraction number, the therapy duration can be estimated, assuming 5 fractions per week.
*Corresponding publications:* Balmanoukian et al., 2012 ^10^, Ebrahimi et al., 2021 ^55^, Ebrahimi et al., 2022 ^56^, Heier et al., 1975 ^68^, Ni et al., 2022 ^58^, Shiraishi et al., 2018 ^33^, Sumiya et al., 2021 ^59^, Wild et al., 2015 ^77^, Yang et al., 2024 ^78^.

When datasets are based on groups of patients with varying fractionation schemes, the median number of fractions was used.
*Corresponding publications:* Chen et al., 2021 ^49^, Cho et al., 2022 ^72^, Cozzarini et al., 2016 ^80^, Heo et al., 2022 ^76^, Ku et al., 2024 ^83^, Lee et al., 2020 ^44^, Liu et al., 2018 ^65^, Ng et al., 2020 ^66^, Xiang et al., 2022 ^82^.

If some patients in the group had shorter therapy durations but there are data points on the plot with longer therapy duration, longer therapy duration was assumed for the entire cohort to avoid omitting patients with longer durations.
*Corresponding publications:* Byun et al., 2019 ^69^, De et al., 2021 ^70^, Park et al., 2019 ^45^, Weeke et al., 1970 ^93^, Xie et al., 2020 ^67^, Xu et al., 2022 ^88^, Zhou et al., 2019 ^63^.

3. Accounting for institutional standards

If no dose or fractionation data are available, standard protocols based on prior knowledge are assumed, inferred from other publications by the same authors with similar study characteristics (e.g., cancer type, publication year, irradiation type).

*Corresponding publications:* Campian et al., 2013 ^11^, Campian et al., 2014 ^64^, Davuluri et al., 2017 ^34^, Deng et al., 2019 ^53^, Fang et al., 2018 ^57^, Grossman et al., 2011 ^12^, Jin et al., 2020 ^42^, Mendez et al., 2016 ^47^, Rudra et al., 2018 ^48^, Wang et al., 2020 ^60^, Yang et al., 2022 ^52^.

4. Exploiting plot data or nadir points

If previous steps are not applicable, either the last available time point was used, if it apparently marks end of therapy, or the time of the measured nadir was used if also post-treatment data are available.

*Corresponding publications:* Cronkite et al., 1971 ^91^, Durante, 2024 ^54^, Kim et al., 2024 ^71^, Wu et al., 2016 ^51^.

For datasets of Meuret et al., 1971 ^41^ with irregular fractionation (e.g., frequent fractions over a short period followed by pauses), the duration of the frequent fractionation period as the therapy duration was used (estimated from plots visually).

If ambiguous values existed for estimated therapy duration, the highest one was chosen to avoid loss of data points by underestimation.

*
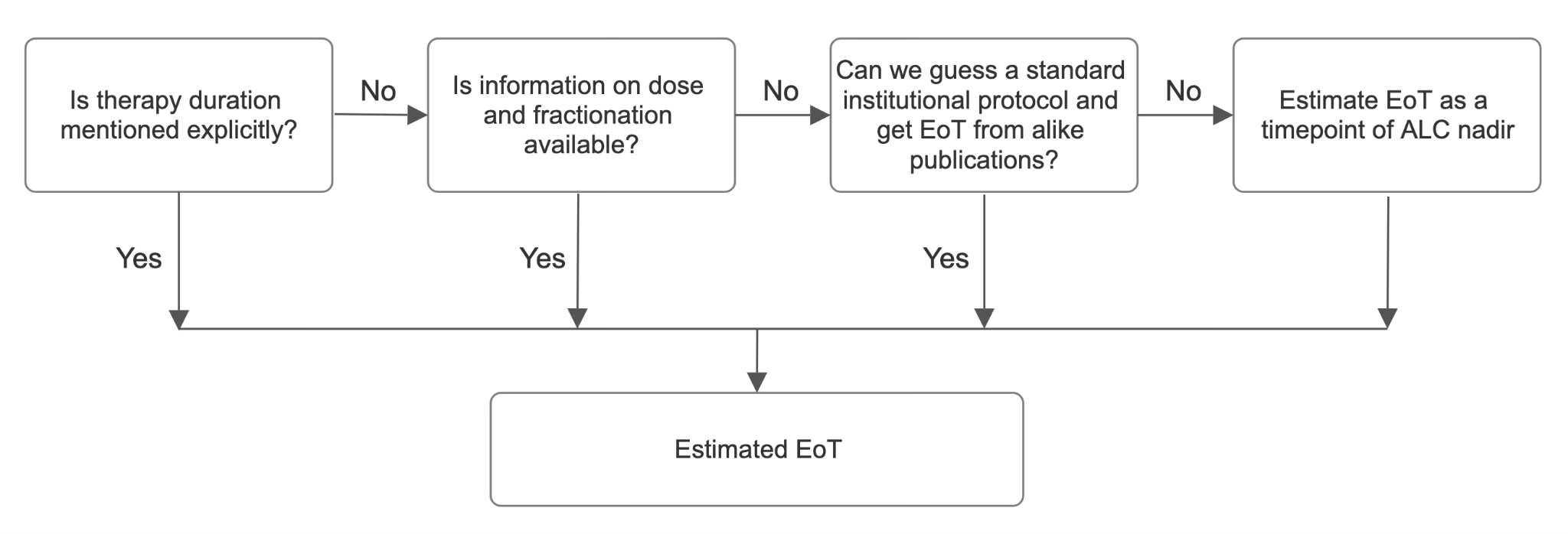
*

**Figure A1.** Schematic of the algorithm for determining the end of therapy in datasets.

**3. The distribution of publication years**

A significant increase in research interest surrounding RIL over the past decade was observed among the 52 studies of the database. The decline in the application of ECIB in clinical practice during the mid-1970s was attributed to limitations in efficiency and practicality of the treatment method.


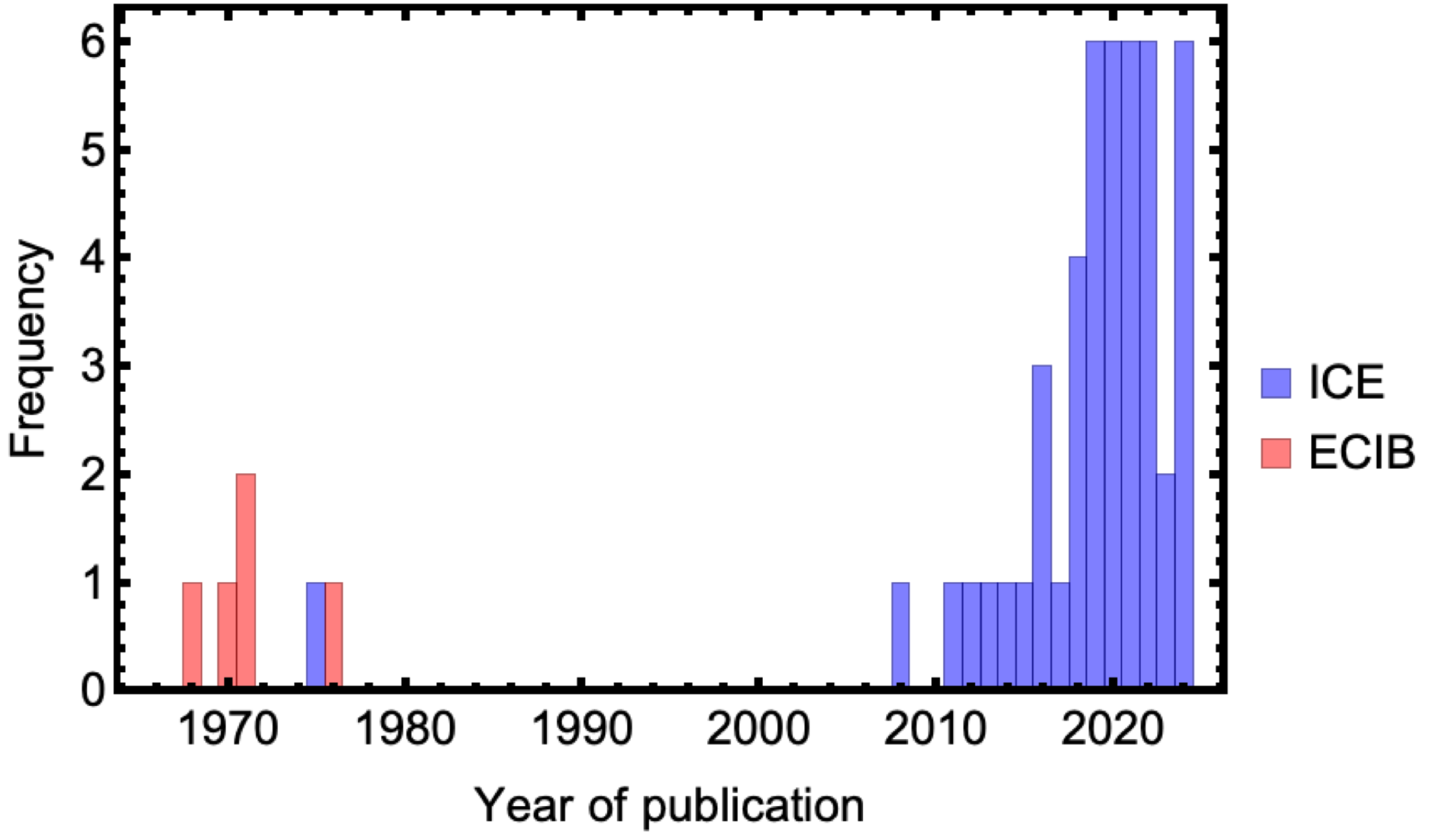


**Figure A2.** Histogram showing the publication years of the studies in the database.

**4. Radiotherapy sites and immune response metrics**

Table A3 contains 53 publication entries, one more than the database, because the study of Ellsworth et al. ^79^ provides datasets for both the Mixed and TBI category. Cervical and prostate cancers are included in the category of pelvic cancers. Esophageal cancer had the highest frequency within the reviewed publications. Other frequently mentioned cancers include pancreatic cancer, glioblastoma, prostate, and non-small cell lung cancer (NSCLC).

**Table A3.** Irradiated sites / organs of reviewed datasets. Numbers of publications (N_p_), datasets (N_d_), uncombined datasets (N_uc_) therein and datasets on single patient (N_s_) therein, indications for radiotherapy, estimated EoT-RLCs, and post-treatment RLC level dynamics for irradiated sites and organs are given. Medians and ranges were calculated based on all datasets for each corresponding site/organ. The number of combined datasets can be obtained by subtracting the number of uncombined datasets from the total dataset count. Similarly, the number of cohort datasets can be retrieved by subtracting the number of datasets for single patients from the total dataset count. In comparison to Table 1, Digestive/Gastrointestinal tumors were subdivided here into Anal, Esophageal, Liver, and Pancreatic cancers. Likewise, Musculoskeletal tumors were divided into Soft tissue cancers and Bone cancers, while CNS cancers were presented as Brain cancers. The term “after” denotes a specific time point, whereas “within” indicates an interval of time.

| **Irradiated site / organ** | **N_p_** | **N_d_**  **N_uc_ / N_s_** | **Indication for radiotherapy** | **EoT-RLC, %: median (range)** | **Post-treatment RLC level recovery, %** |
| --- | --- | --- | --- | --- | --- |
| Anal | 1 | 1  1 / 0 | Anal Squamous Cell carcinoma ^44^ | 24 (24–24) | 43 after 1 year |
| Bones | 1 | 1  1 / 0 | Bone metastasis from hepatocellular carcinoma ^45^ | 53 (53–53) | 76 after 1 year |
| Breast | 2 | 2  2 / 0 | Breast cancer ^49,50^ | 60 (59–61) | ~ 80-90 within 1-3 months |
| Brain | 5 | 8  8 / 0 | Glioblastoma ^12,36,46–48^ | 62 (49–79) | 50-90 within 1 year |
| Esophagus | 13 | 47  42 / 25 | Esophageal Cancer ^33,34,53–63^ | 20 (5–41) | 50-70 within 2 months; 66-85 within 6 months |
| Head and Neck | 4 | 16  14 / 0 | Head and neck squamous cell carcinoma ^64^,  nasopharyngeal carcinoma ^65,67^,  oropharyngeal cancer ^66^ | 27 (15–57) | 45-55 within 1 year |
| Liver | 3 | 5  4 / 0 | Hepatocellular carcinoma ^69–71^ | 27 (15–29) | 54 after 7 weeks; 73 after 1 year |
| Lung | 5 | 8  8 / 0 | Non-small-cell lung cancer ^11,72–75^ | 21 (11–32) | 40-60 within 6 months |
| Pancreas | 5 | 22  21/ 15 | Locally advanced pancreatic cancer ^77,78^; pancreatic adenocarcinoma ^10,76^; cholangiocarcinoma or ampullary cancer ^42^ | 34 (7–68) | 40-70 within 1 year |
| Pelvis | 5 | 9  8 / 0 | Prostate Cancer ^80,81^,  Cervical cancer ^51,52^, pelvic malignancies ^82^ | 30 (20–73) | 33-55 within 1 year; 93-96 after 5-10 years |
| Soft tissue | 1 | 1  1 / 0 | Soft tissue sarcoma ^83^ | 36 (36–36) | 78 after 48 weeks |
| Total body (TBI) | 1 | 1  1 / 0 | BM transplantation ^79^ | 1.5 (1.5–1.5) |  |
| Blood (with ECIB) | 5 | 19  19 / 17 | Renal transplantation ^92,93^, Chronic lymphocytic leukemia ^41,90,91^ | 10 (0–66) | 37 after 14 weeks |
| Mixed | 2 | 2  2 / 0 | Seminoma testis in paraaortic and iliac LN and mediastinum ^68^, Pancreas, Esophagus, Anal, Cervix, Biliary tract ^79^ | 20 (15–24) |  |

**5. Treatment modality representation in the included studies**

Table A4 summarizes the frequency of treatment modalities in the studies of the database. Notably, no datasets based solely on SBRT were identified; SBRT was mentioned only in combination with other modalities, hence categorized as “mixed”. As several publications reported data retrieved with different therapy modalities, the sum of publication entries in the table exceeds the number of publications in the database, 52.

**Table A4.** Treatment modalities in the reviewed datasets. The number of publications (N_p_), datasets (N_d_), uncombined datasets (N_uc_), and single-patient datasets (N_s_) is provided. “Mixed” denotes datasets incorporating multiple treatment modalities, while “XRT” refers to photon therapy without a specified modality in the original publication.

| **Treatment modality** | **N_p_** | **N_d_**  **N_uc_ / N_s_** |
| --- | --- | --- |
| Conformal RT (CRT) (including 2D- and 3D-CRT) ^10,11,50,76^ | 4 | 7  6 / 0 |
| IMRT ^33,34,36,54,57,59,61,65,66,71–73,75,80^ | 14 | 25  23 / 0 |
| VMAT (including RapidArc) ^80,82^ | 2 | 2  2 / 0 |
| HTT ^80^ | 1 | 1  1 / 0 |
| XRT ^12,42,47,51,64,68^ | 6 | 22  21 / 15 |
| SBRT | 0* | 0  0 / 0 |
| Mixed photon modalities ^45,46,48,49,52,58,60,62,63,67,69,74,77–81,83^ | 18 | 24  22 / 0 |
| TBI ^79^ | 1 | 1  1 / 0 |
| ECIB ^41,90–93^ | 5 | 19  19 / 17 |
| Proton therapy ^33,34,36,55,57,59,61,73^ | 8 | 18  17 / 10 |
| Mixed proton / photon ^34,44,53,56,57,61,70^ | 7 | 21  18 / 15 |
| Carbon therapy ^54,78^ | 2 | 2  2 / 0 |

*SBRT appears in the table as part of mixed datasets only, with no standalone datasets exclusively dedicated to SBRT.

**6. Exponential fits of the selected RLC datasets**


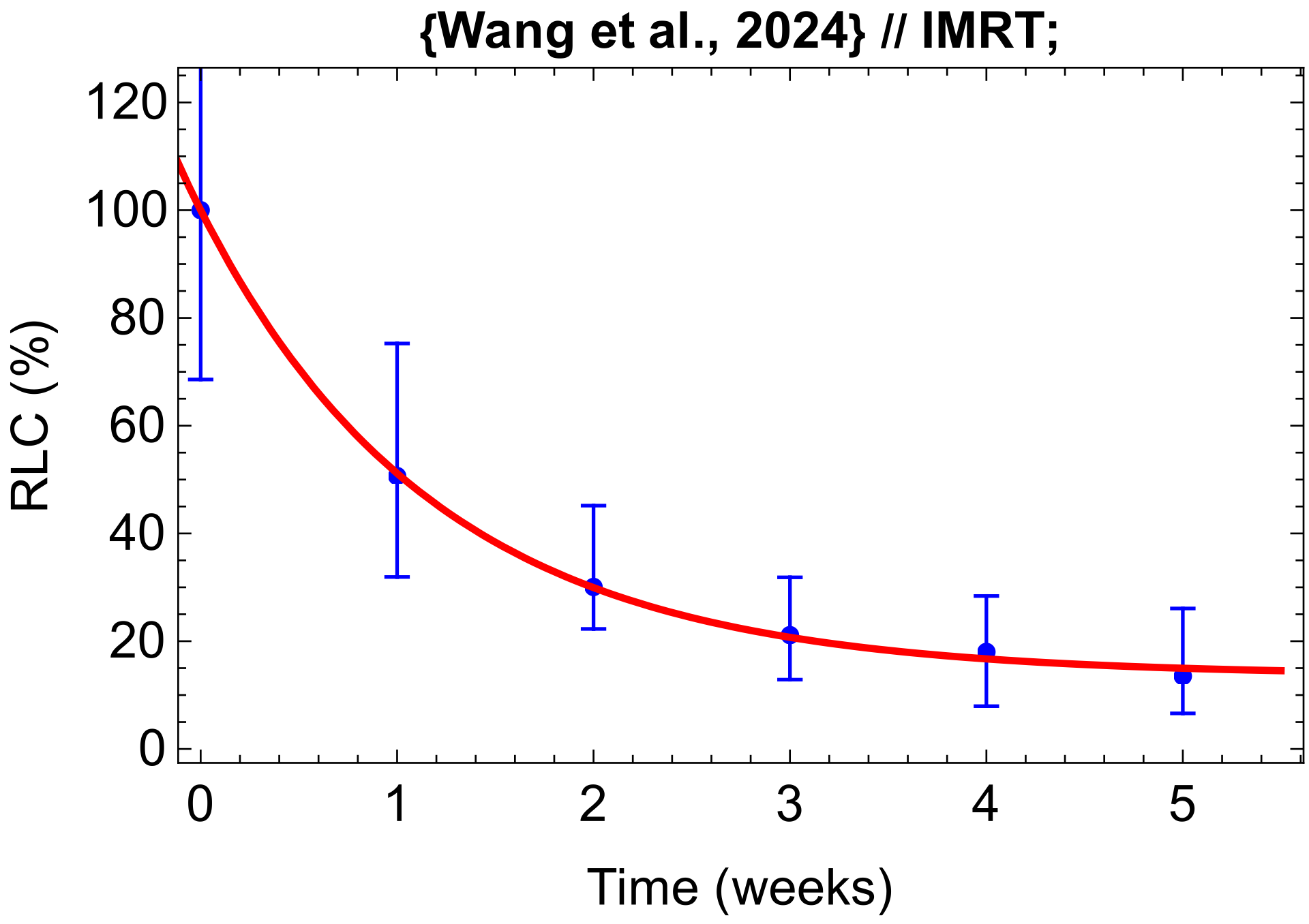

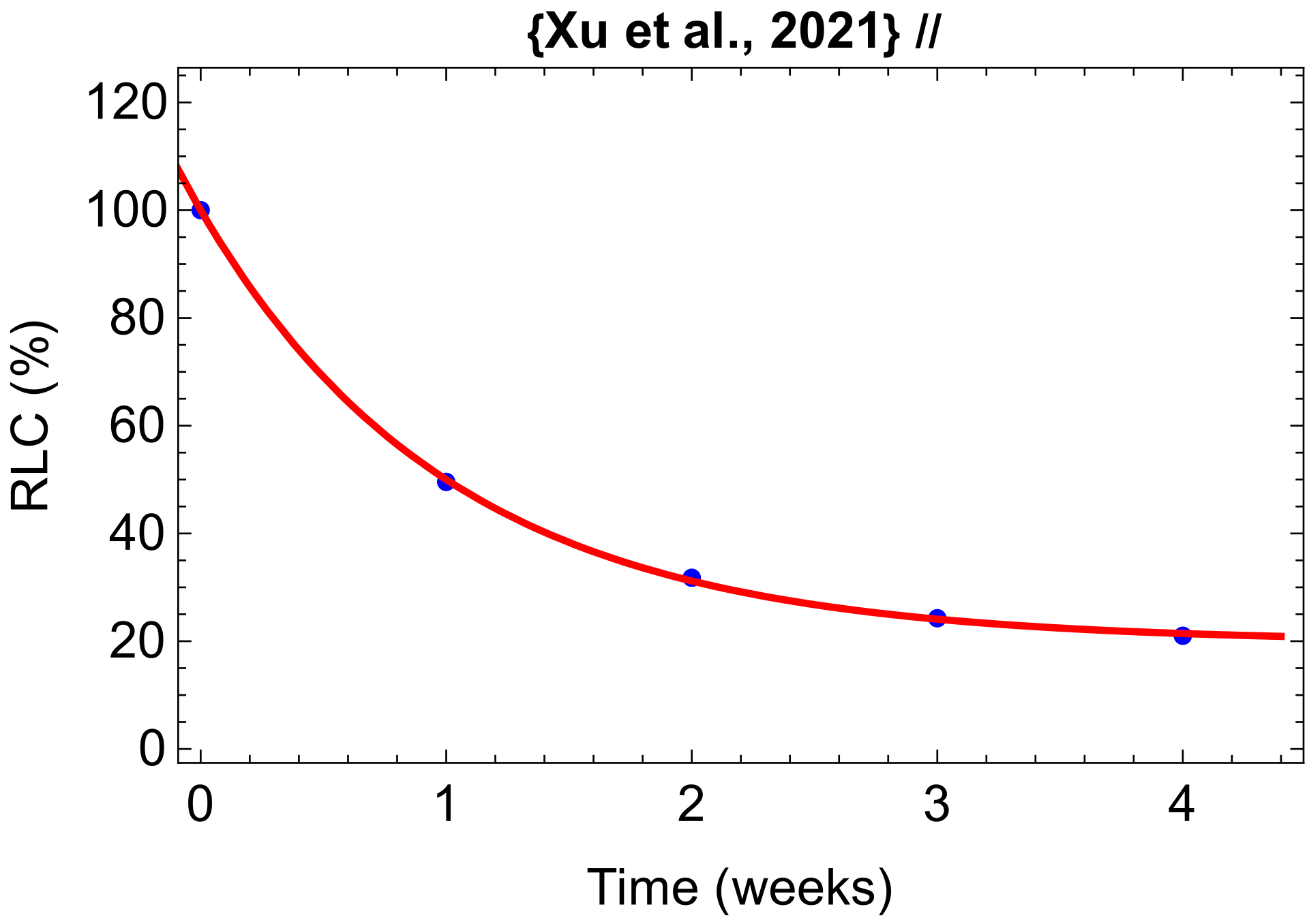

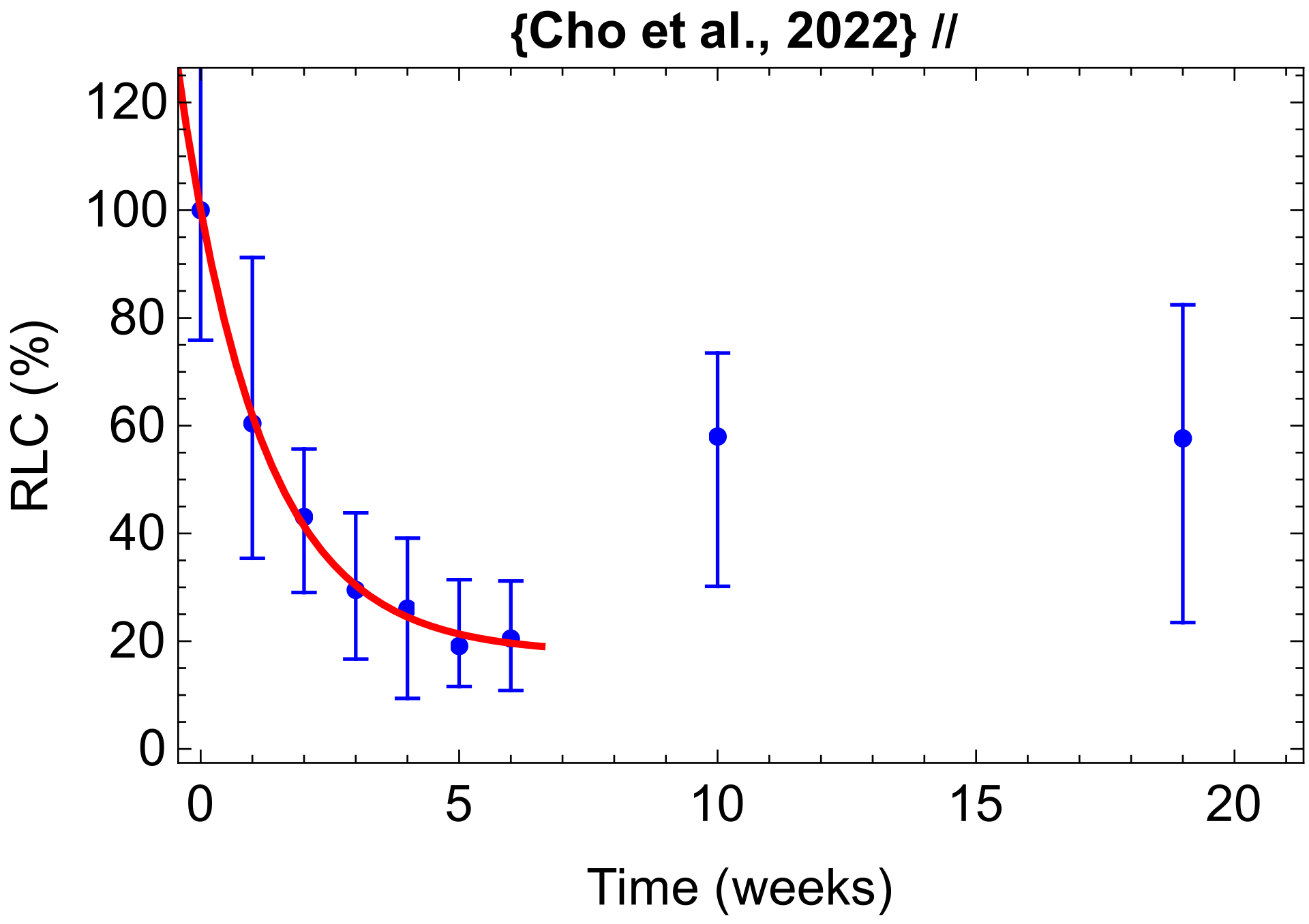

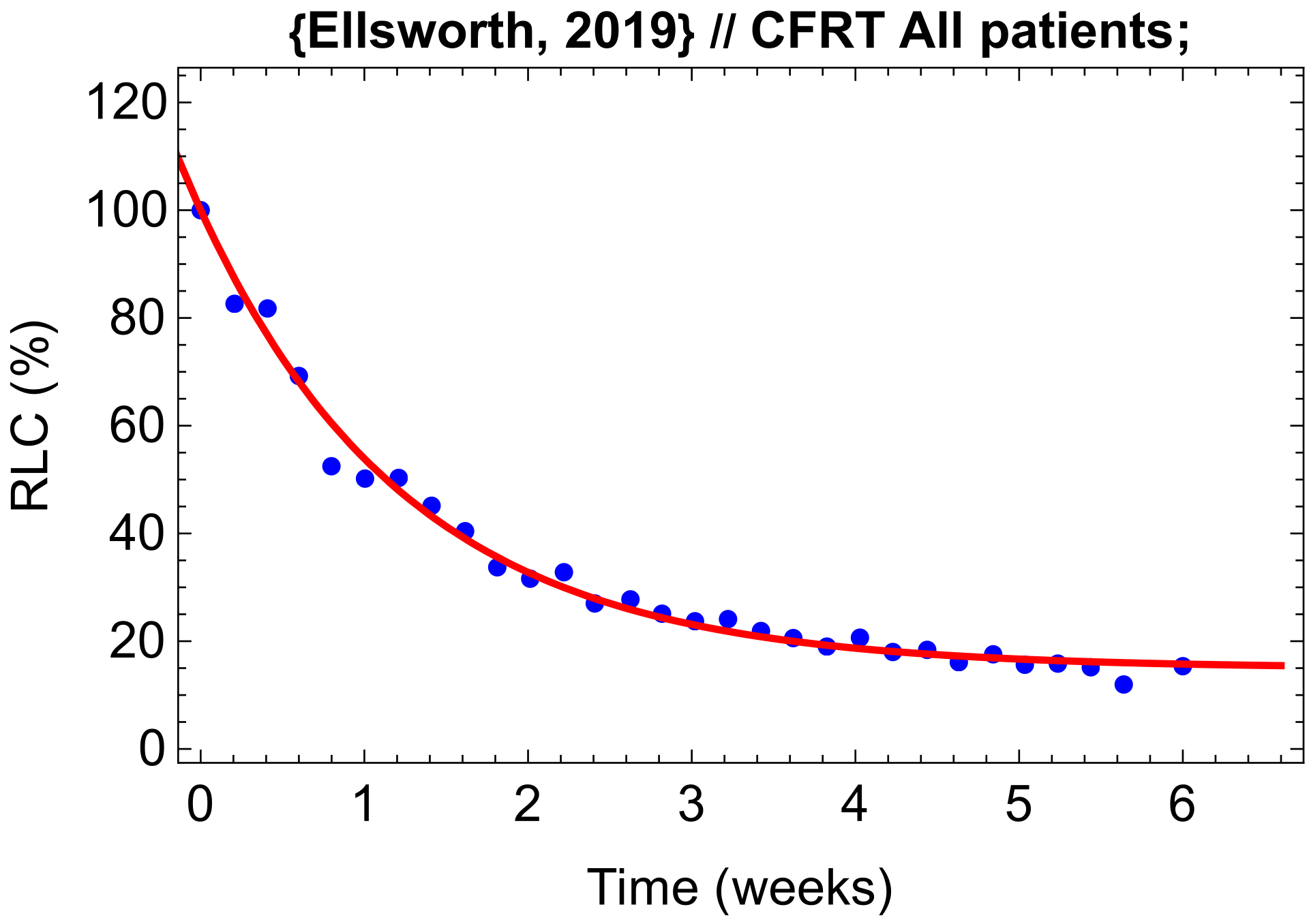

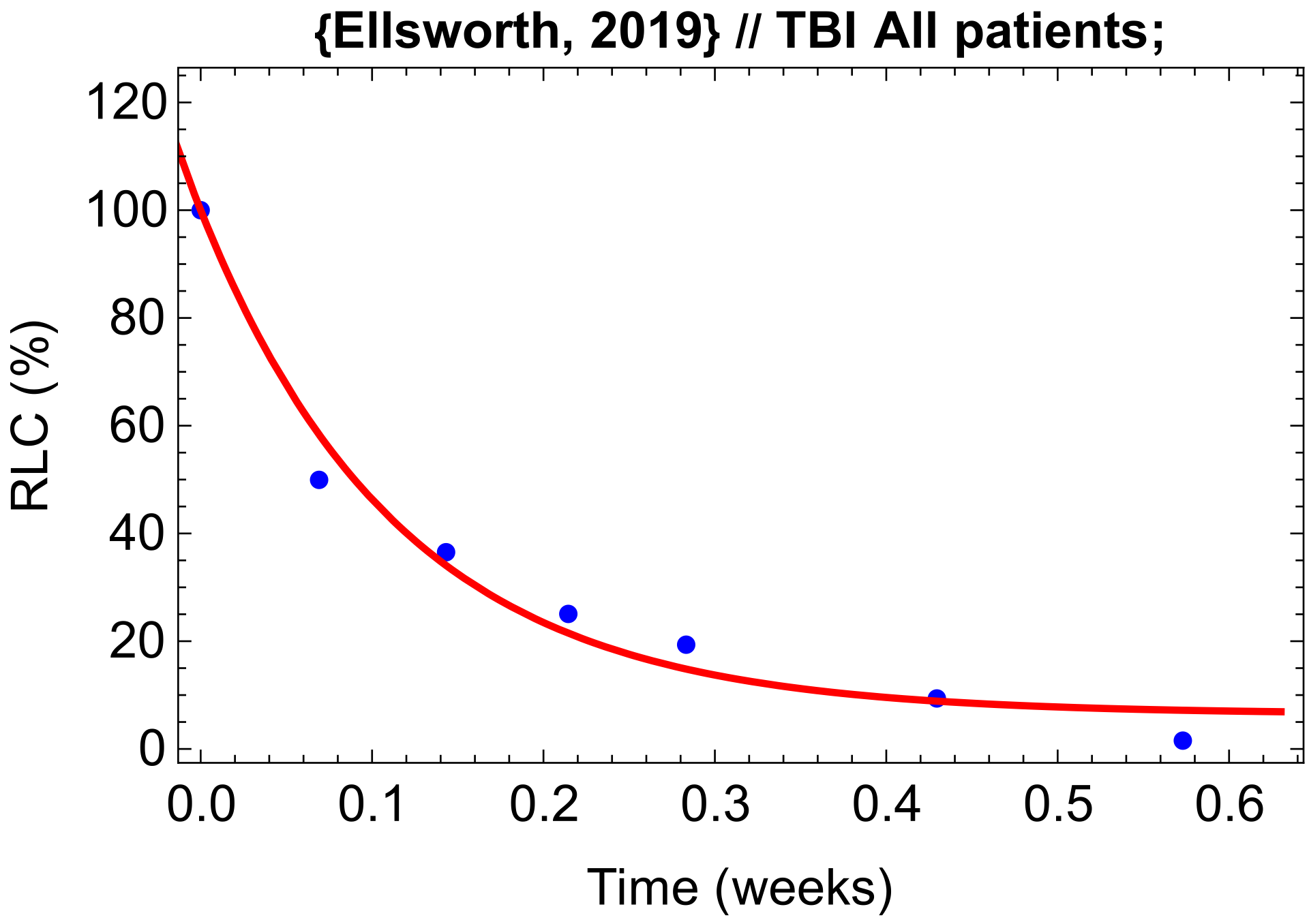

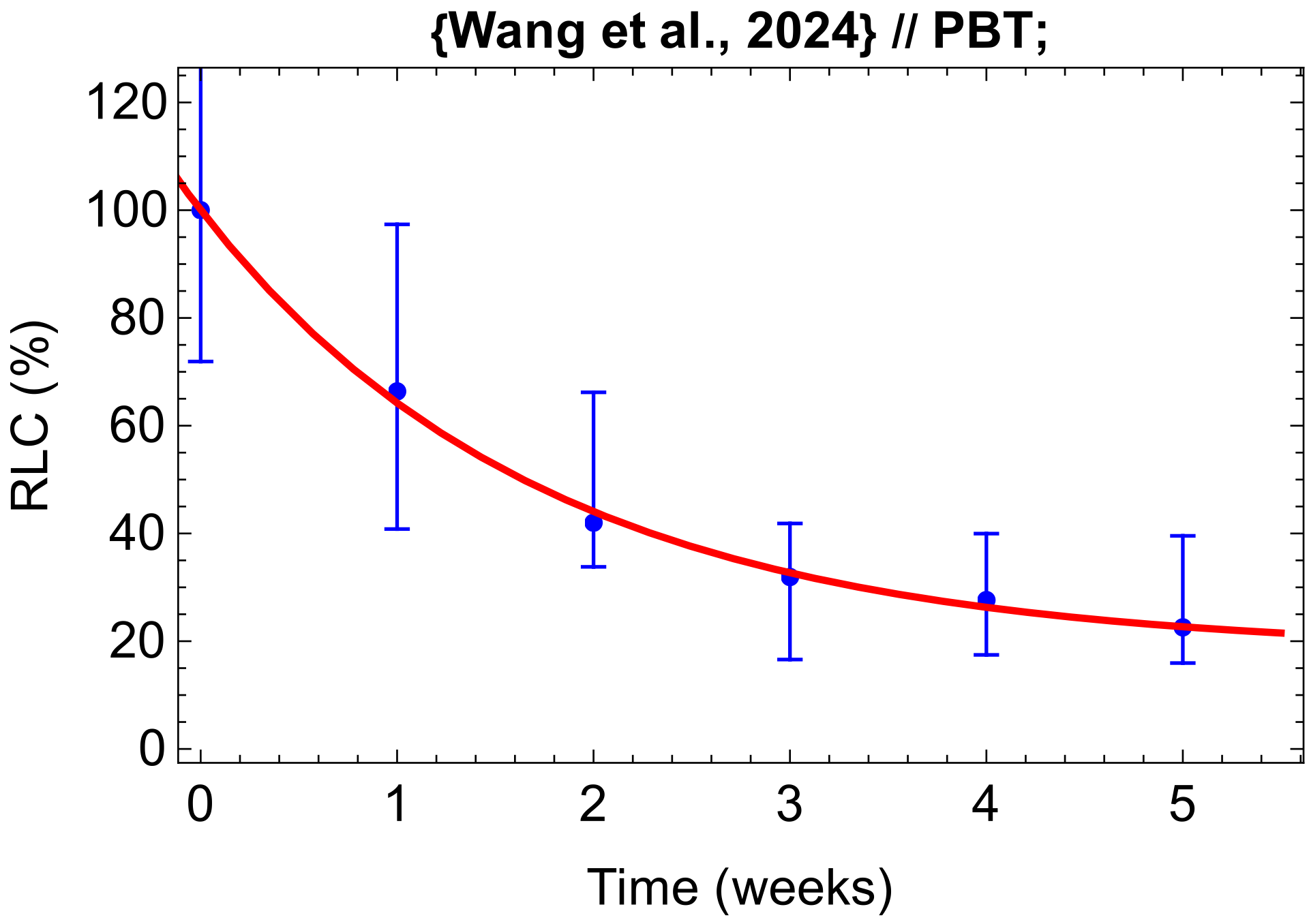

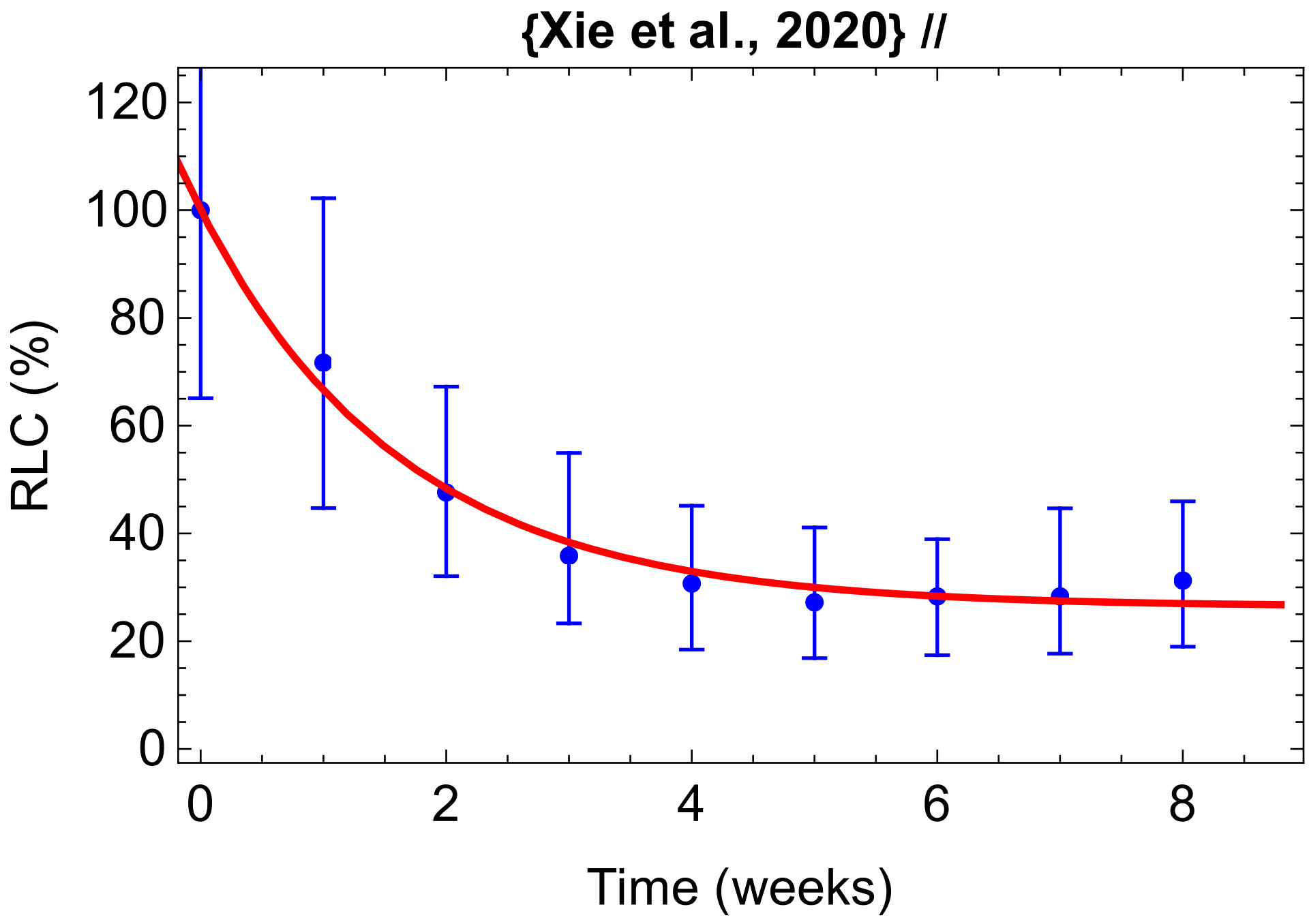

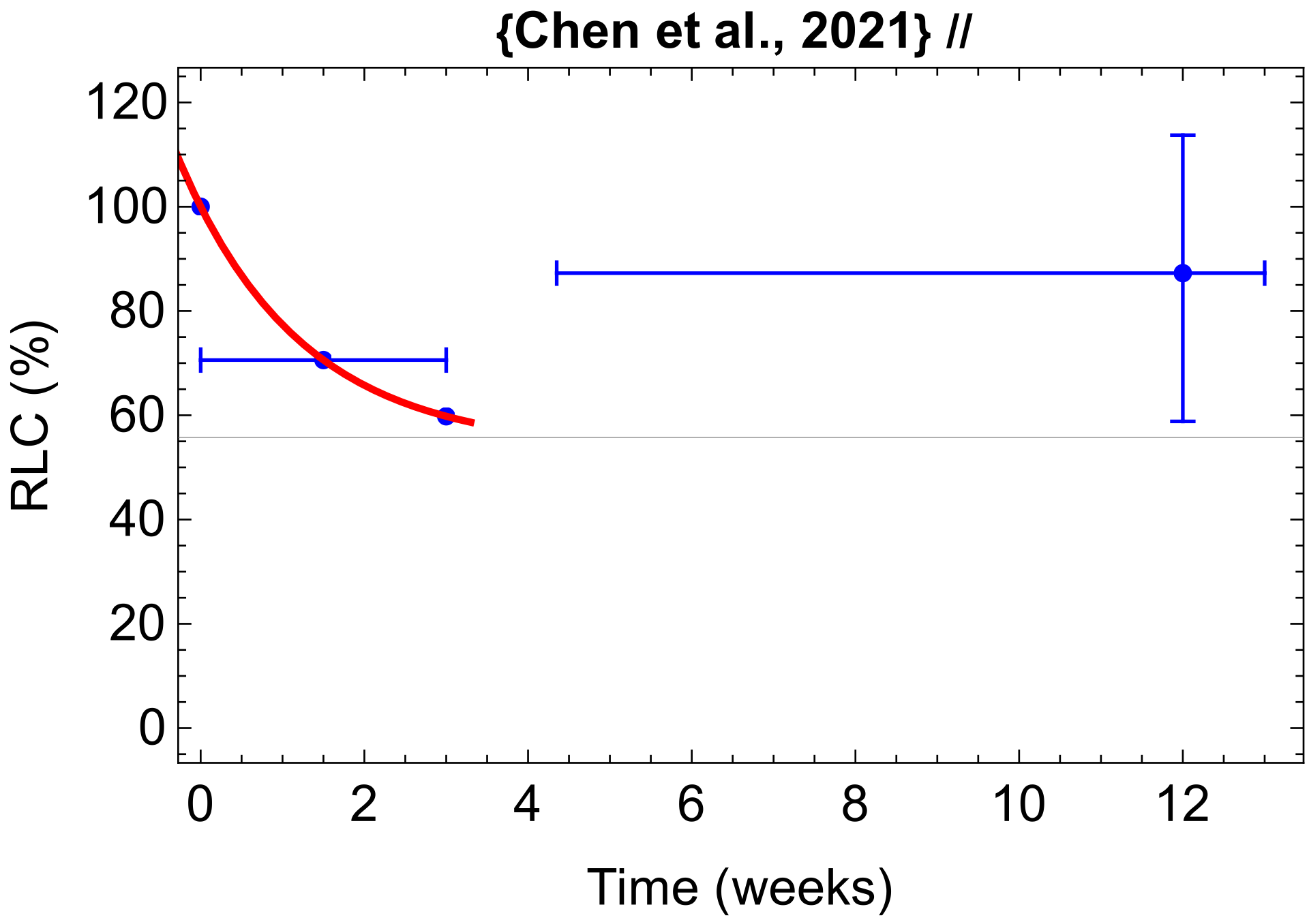

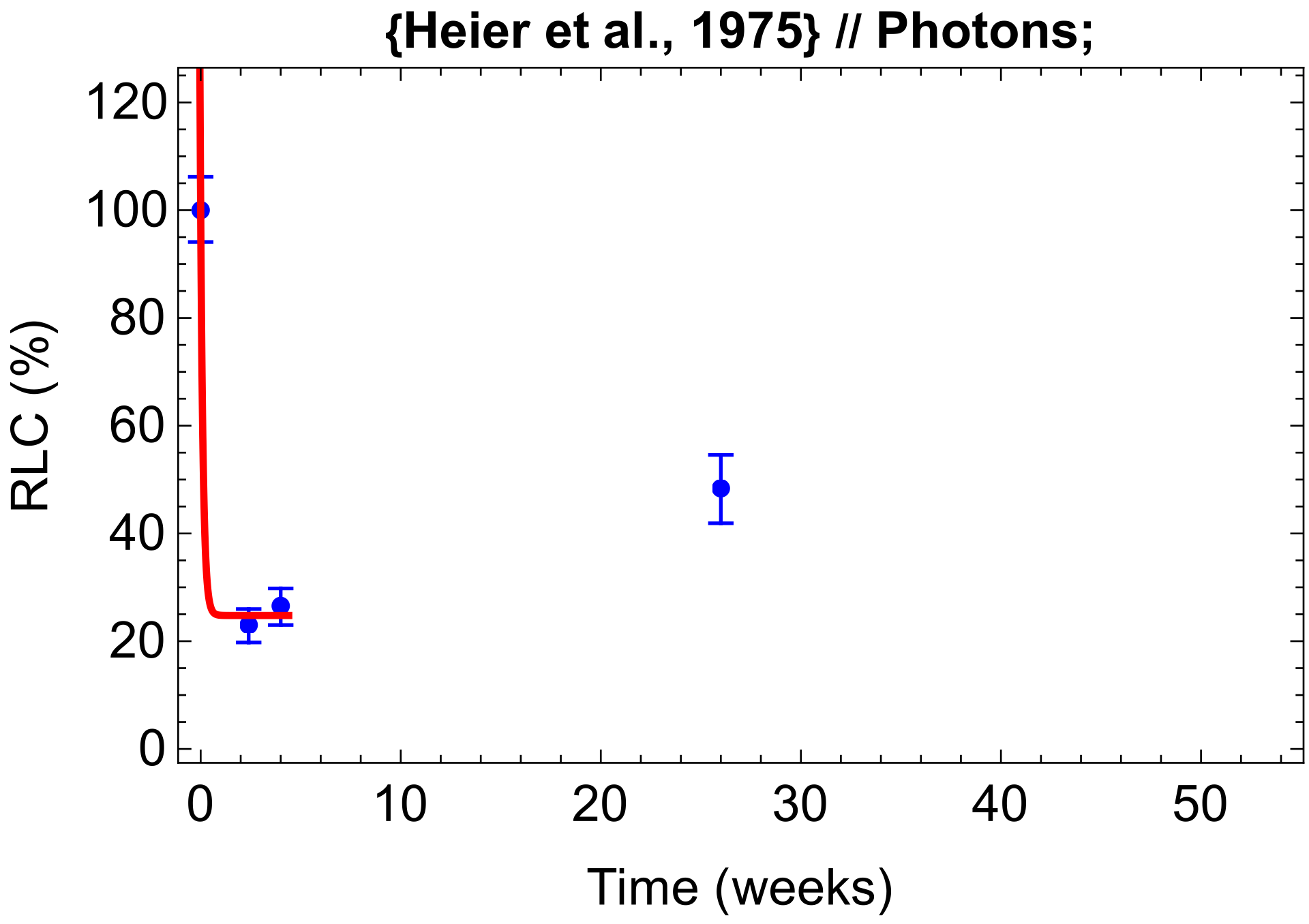

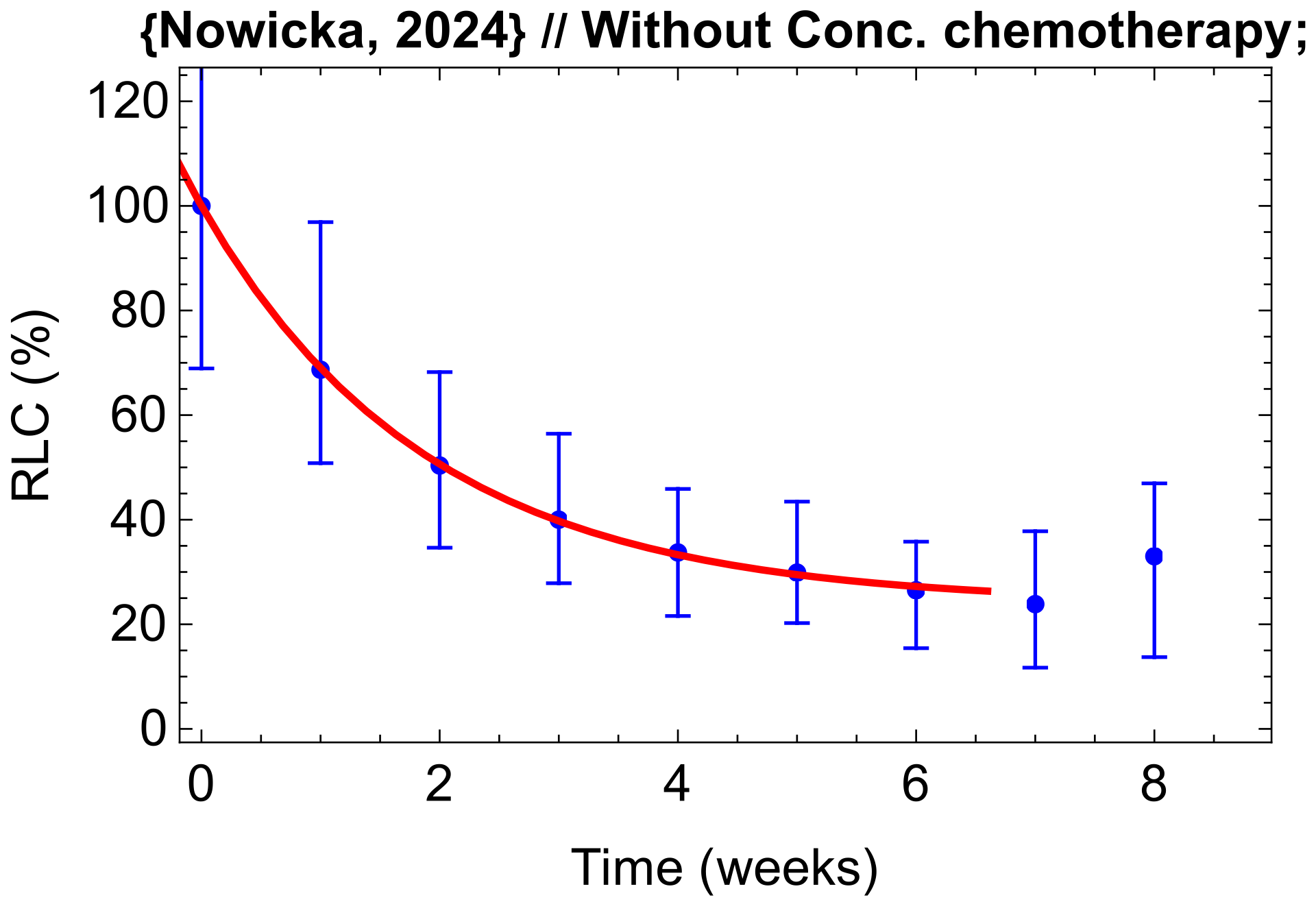

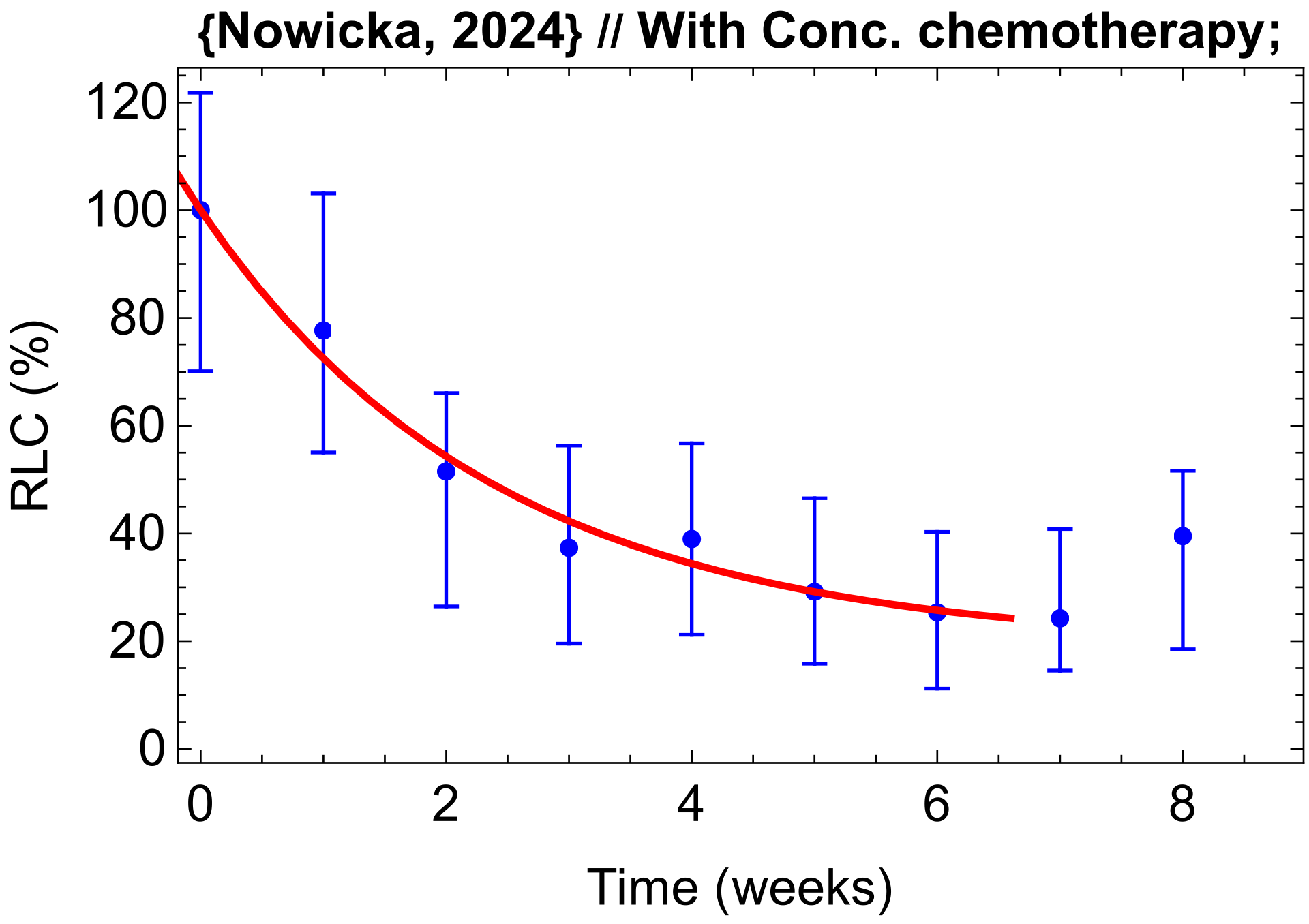

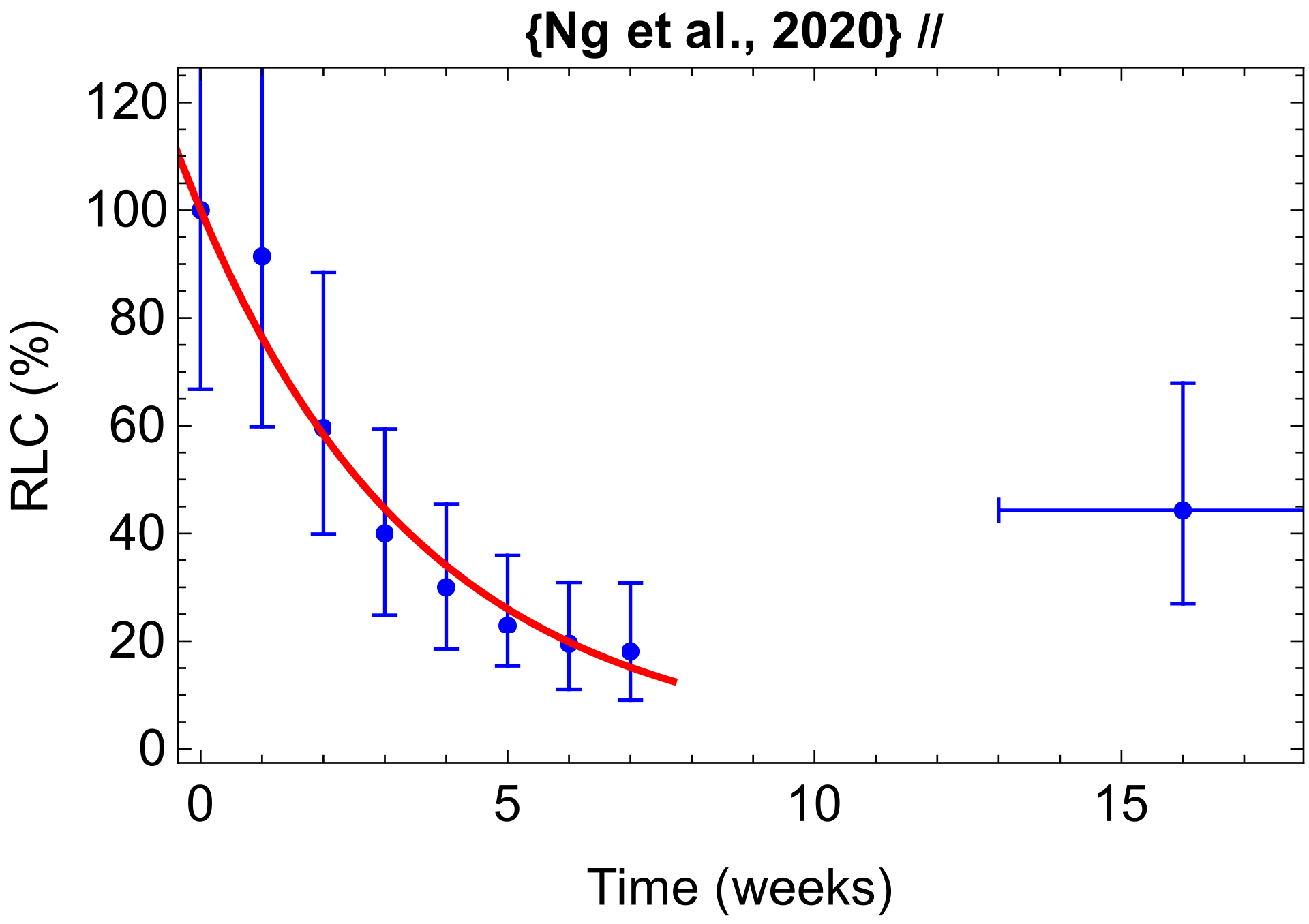

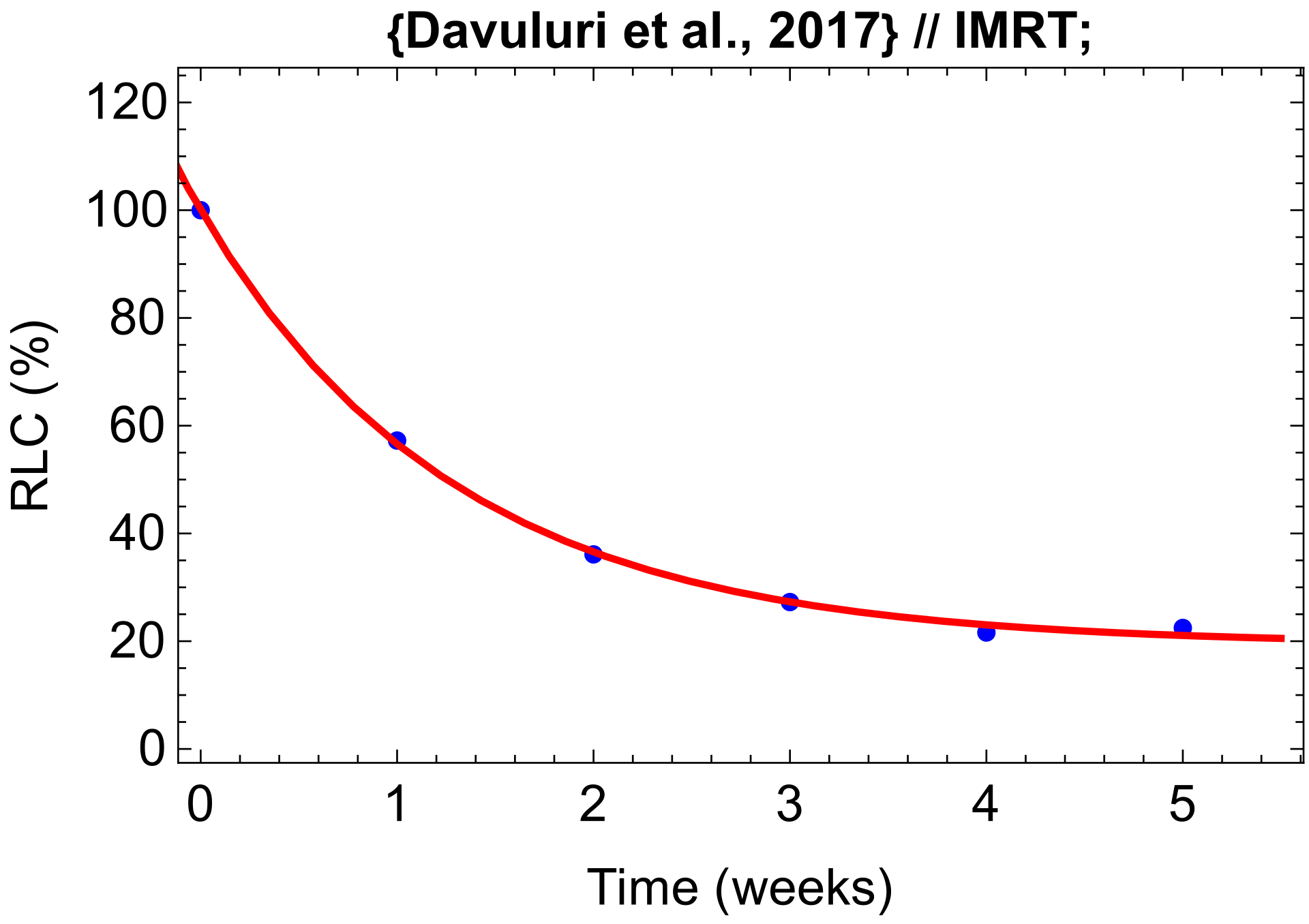

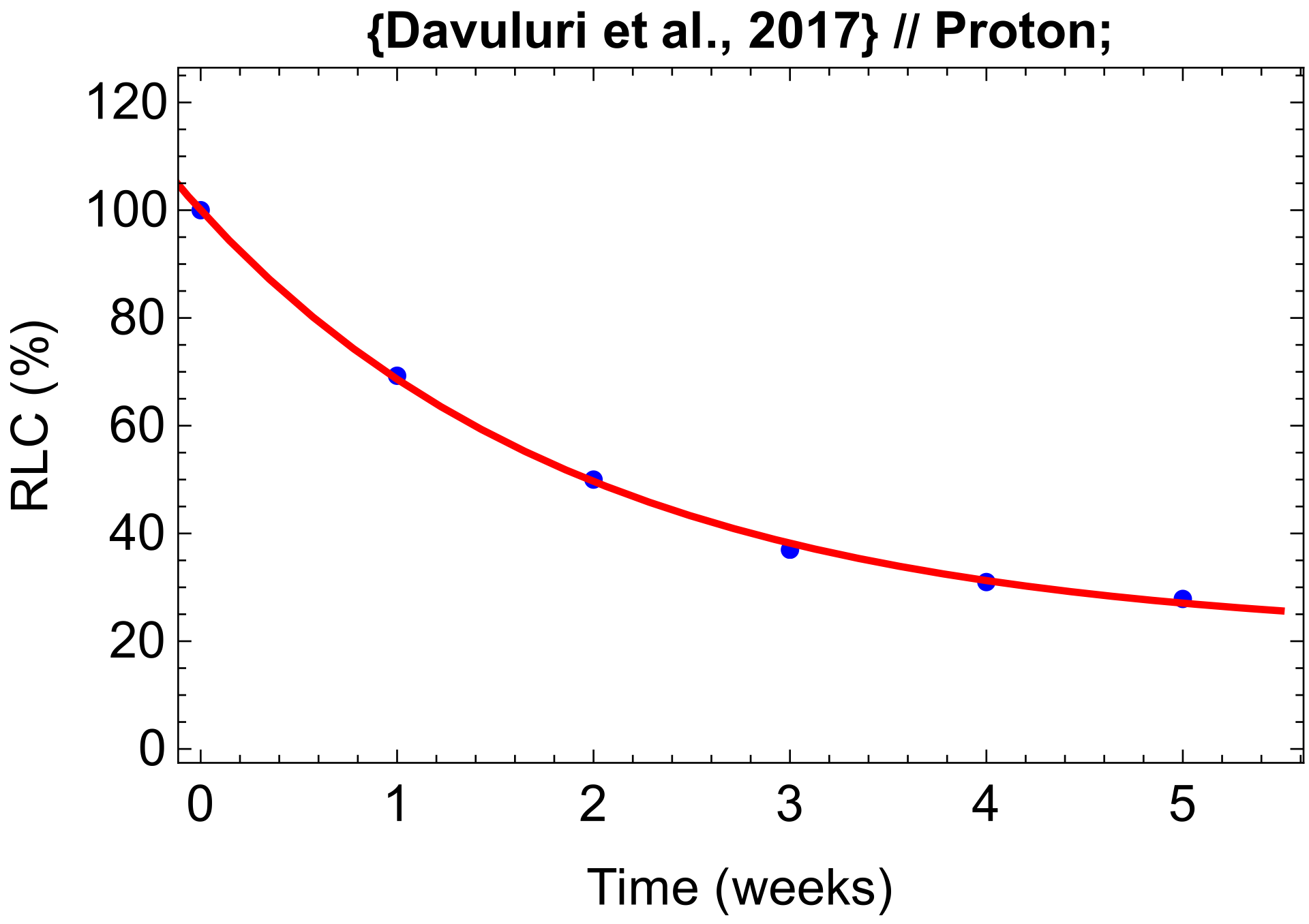

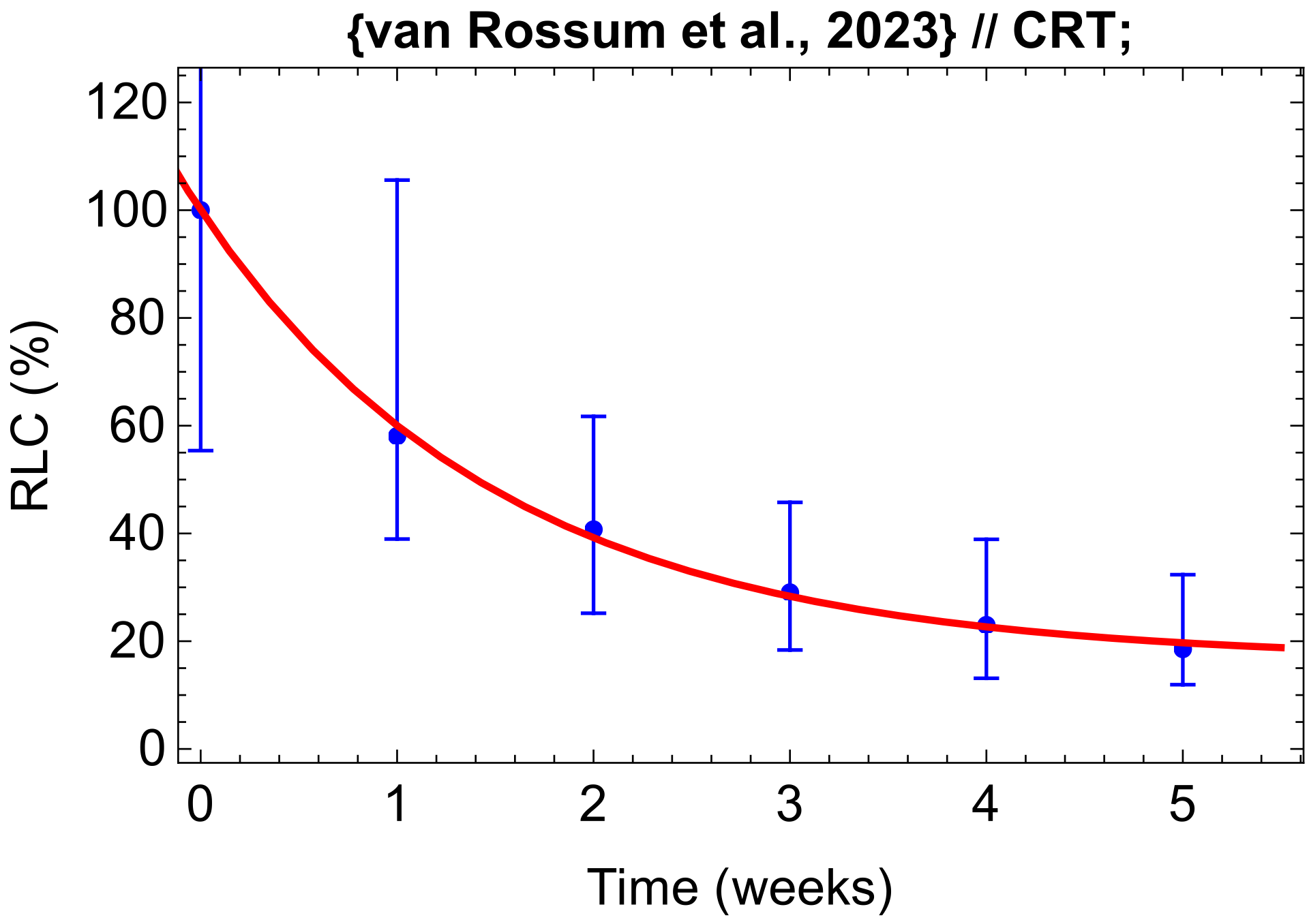

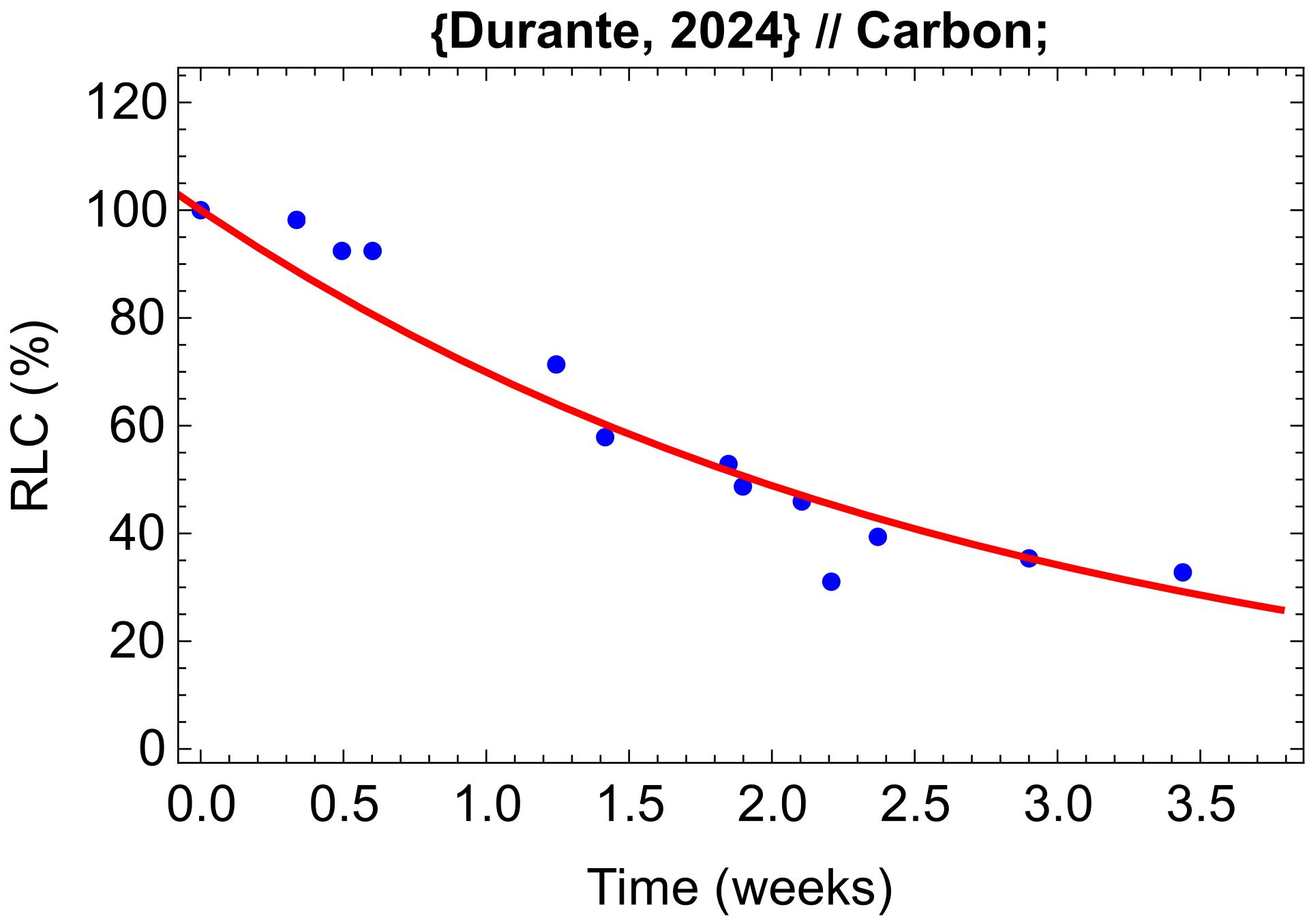

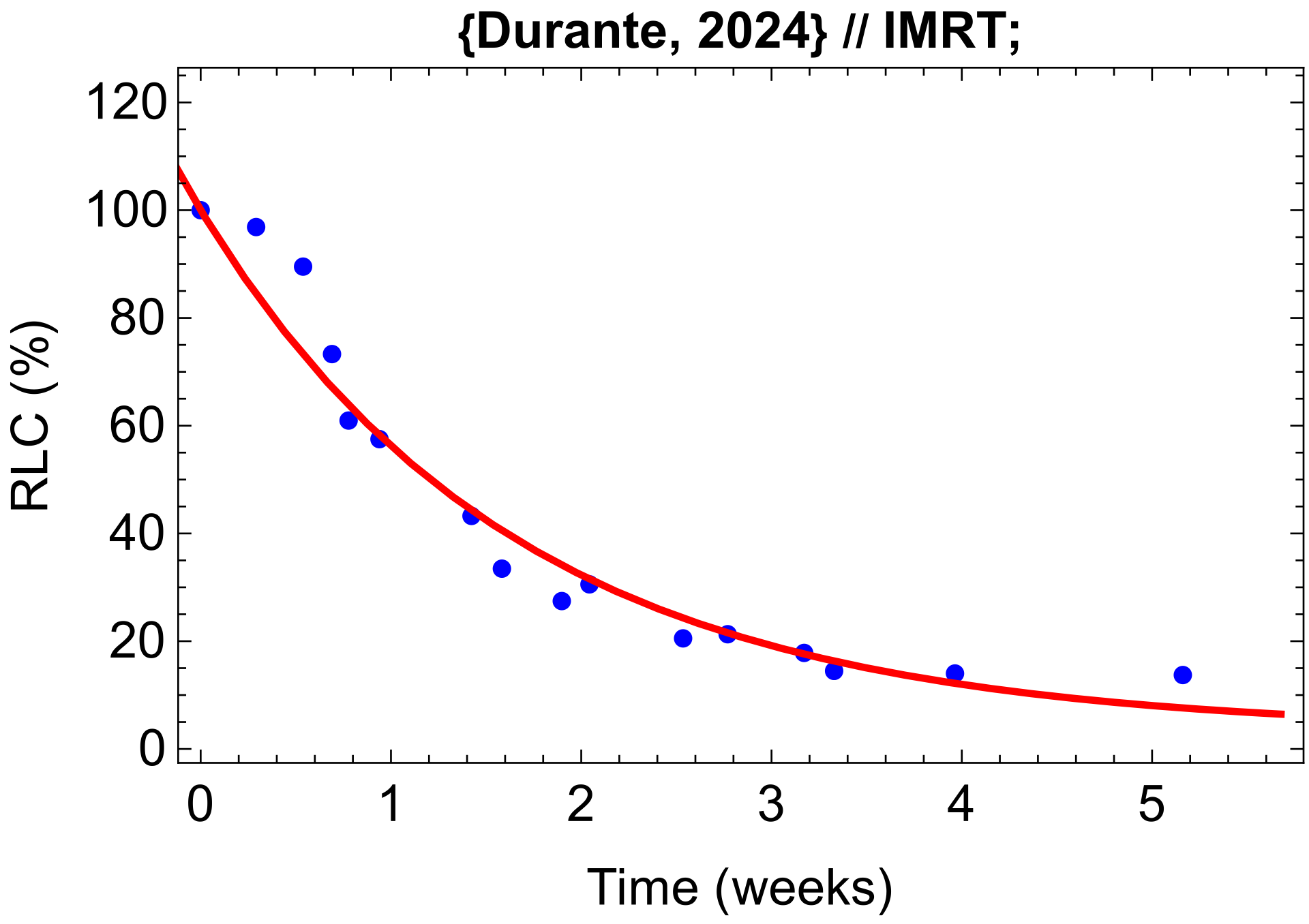

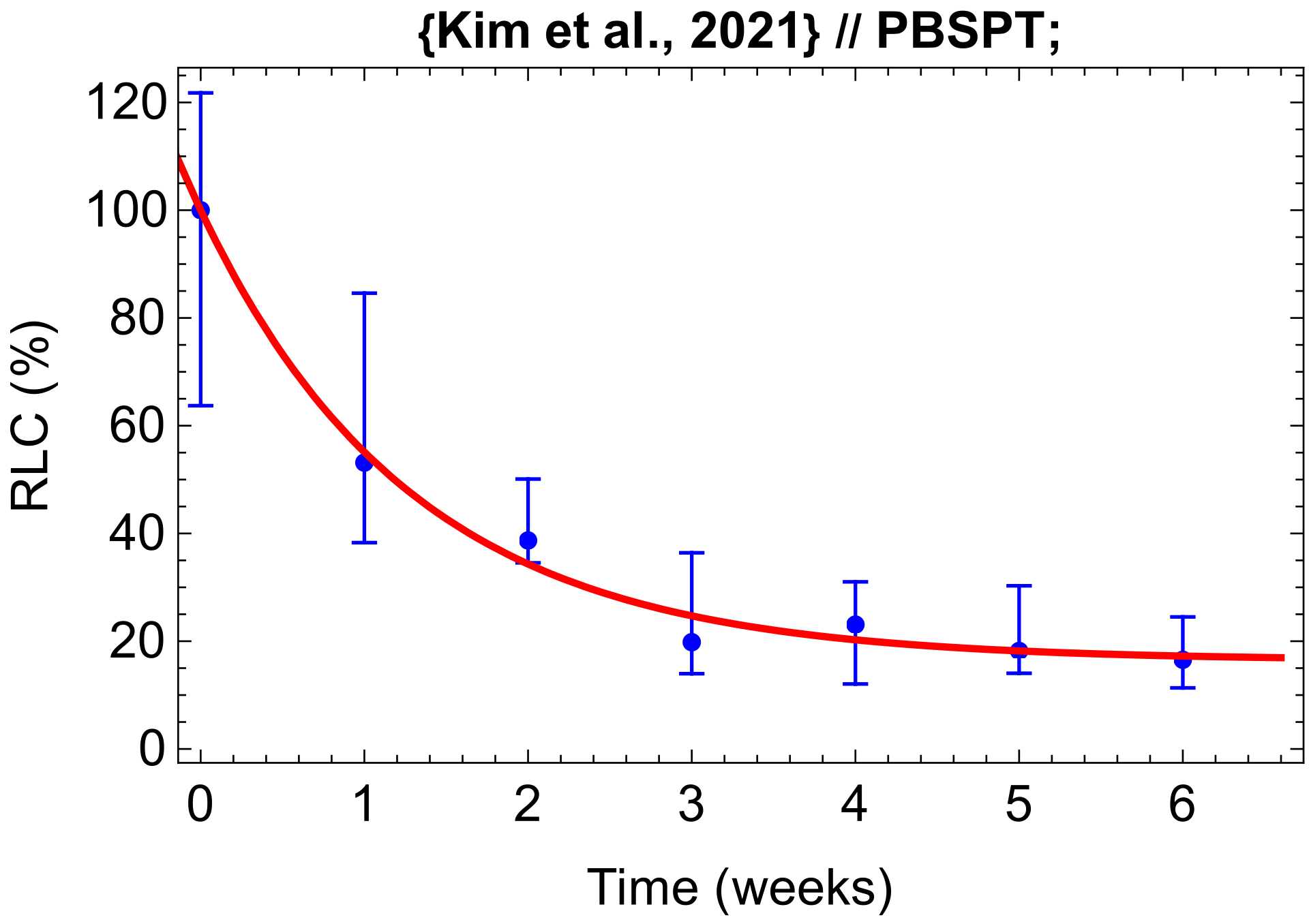

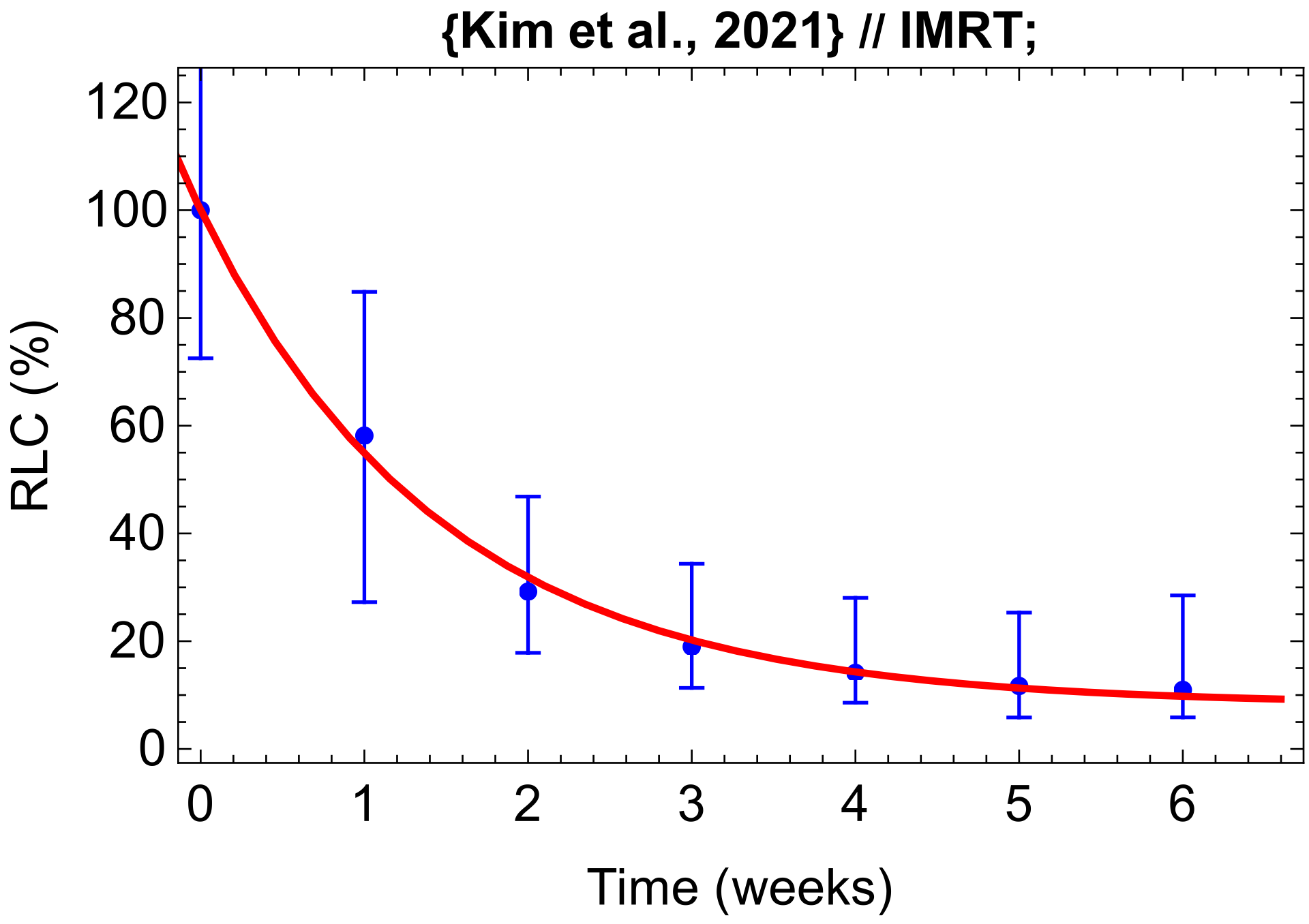

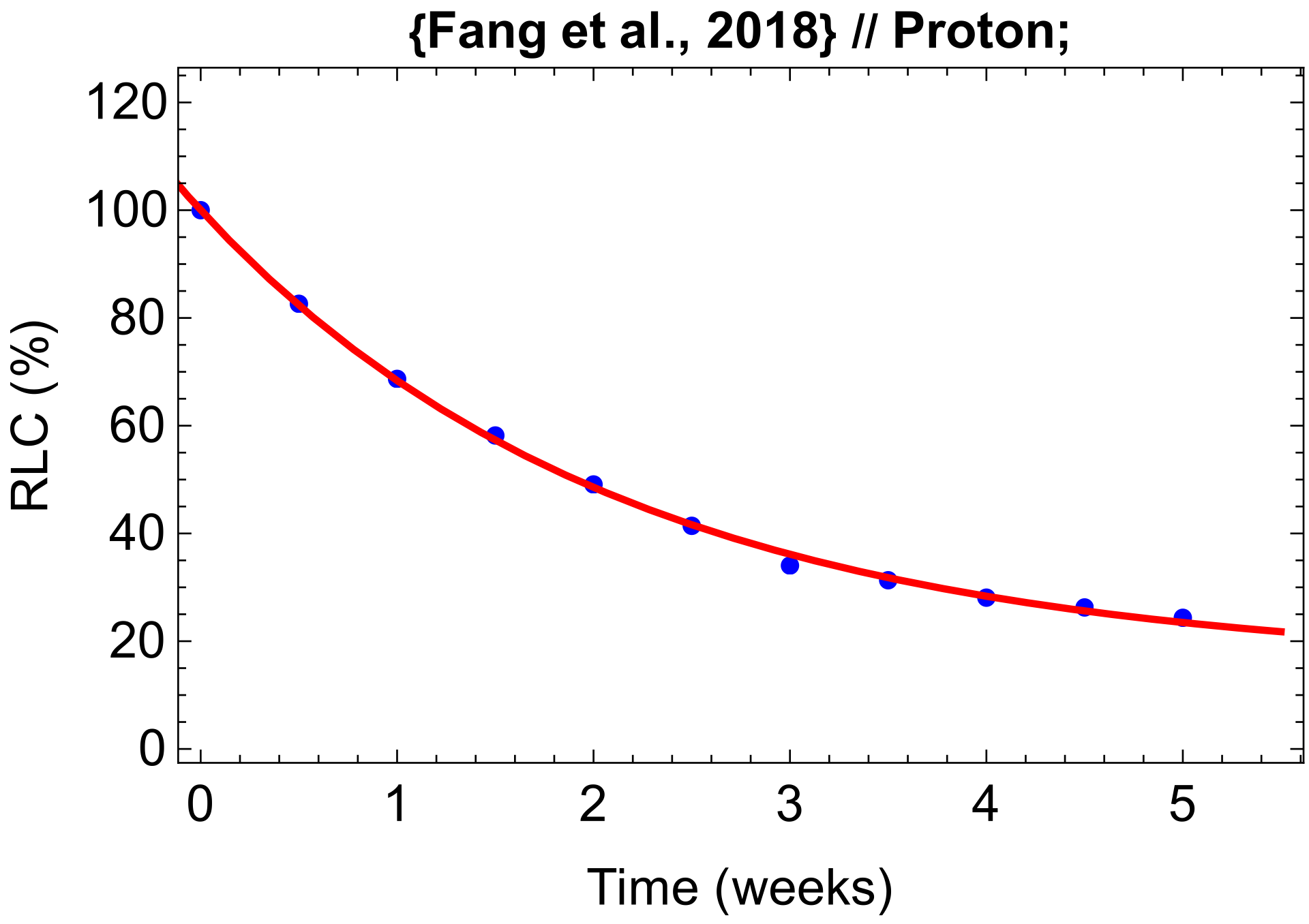

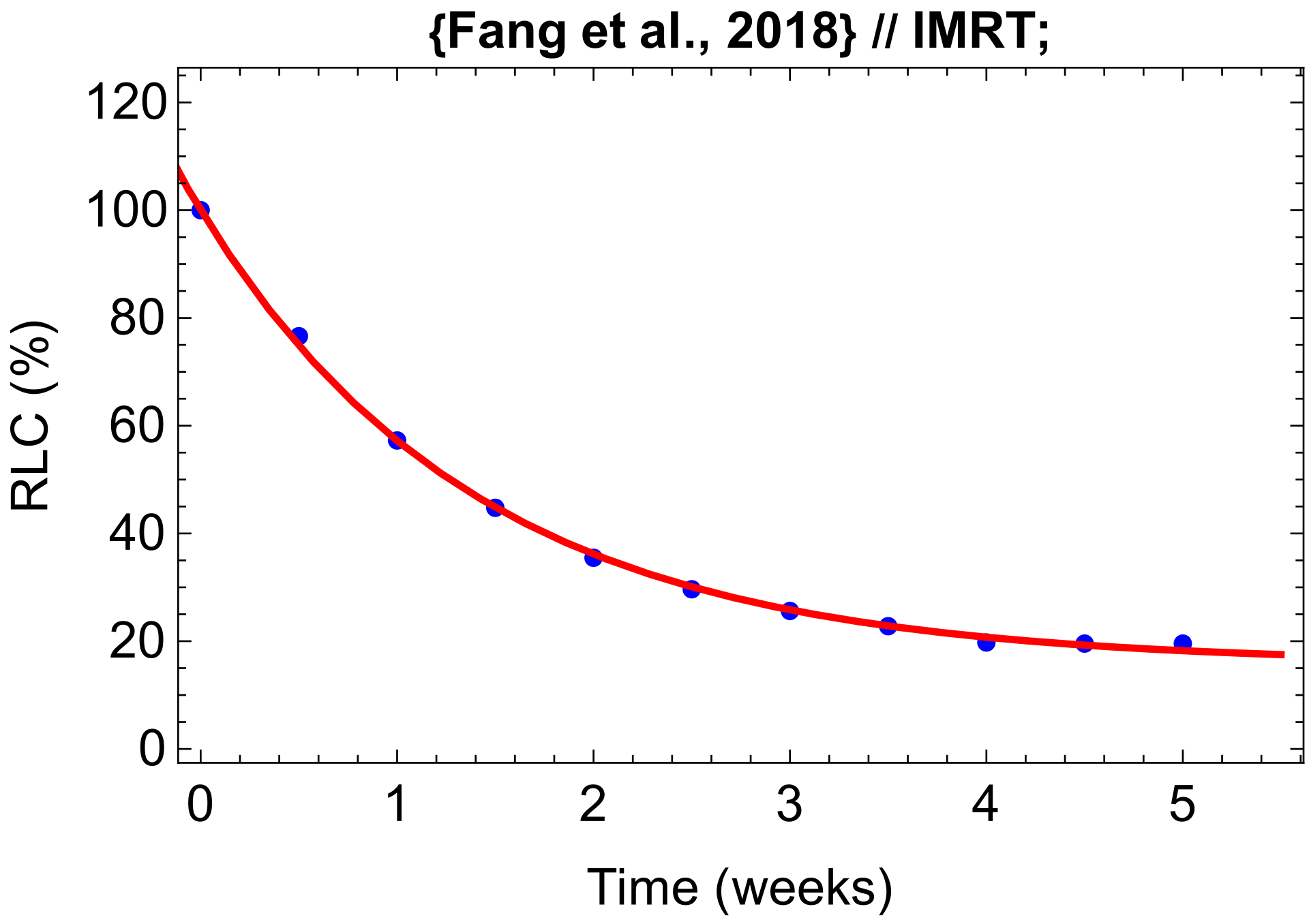

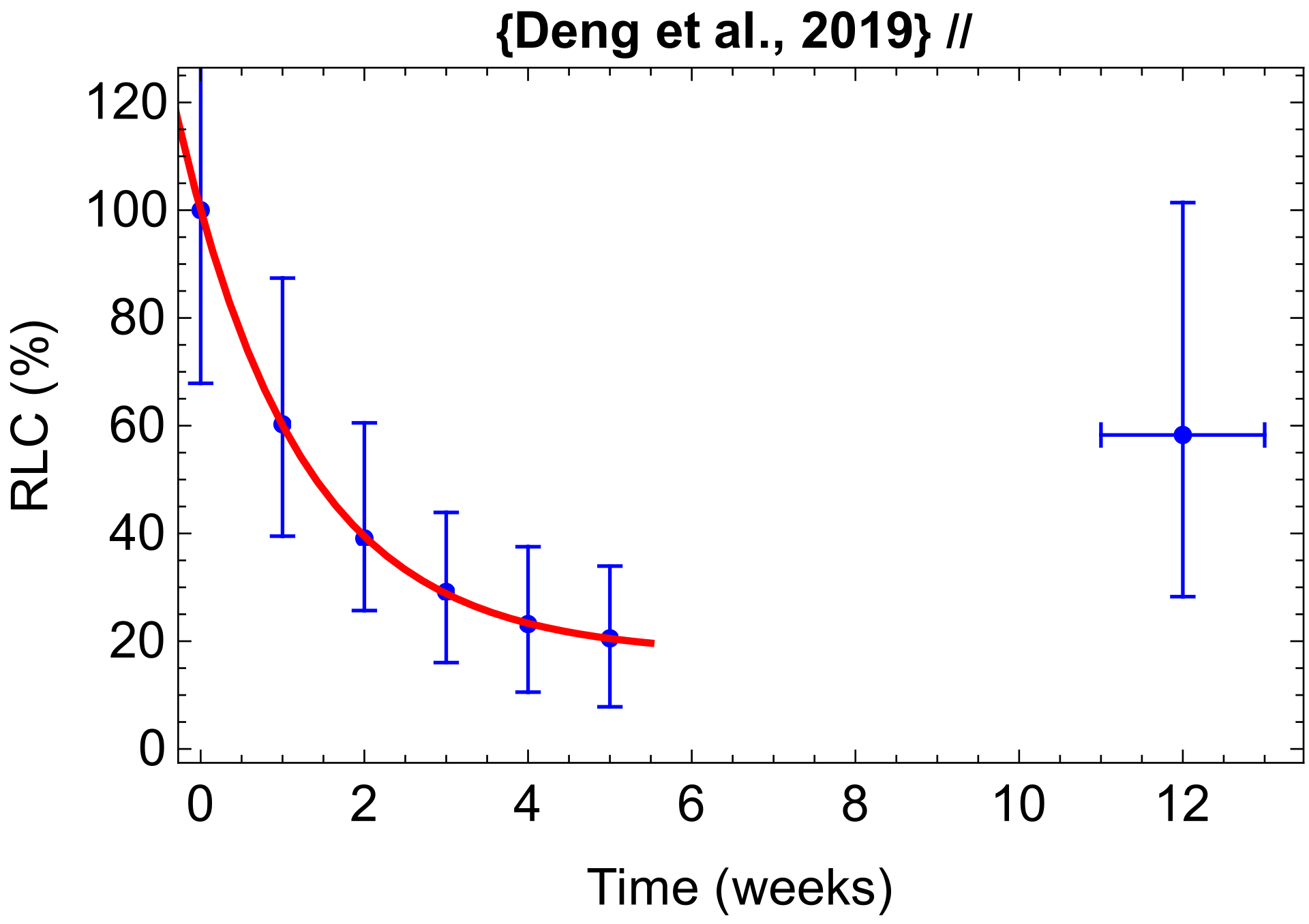

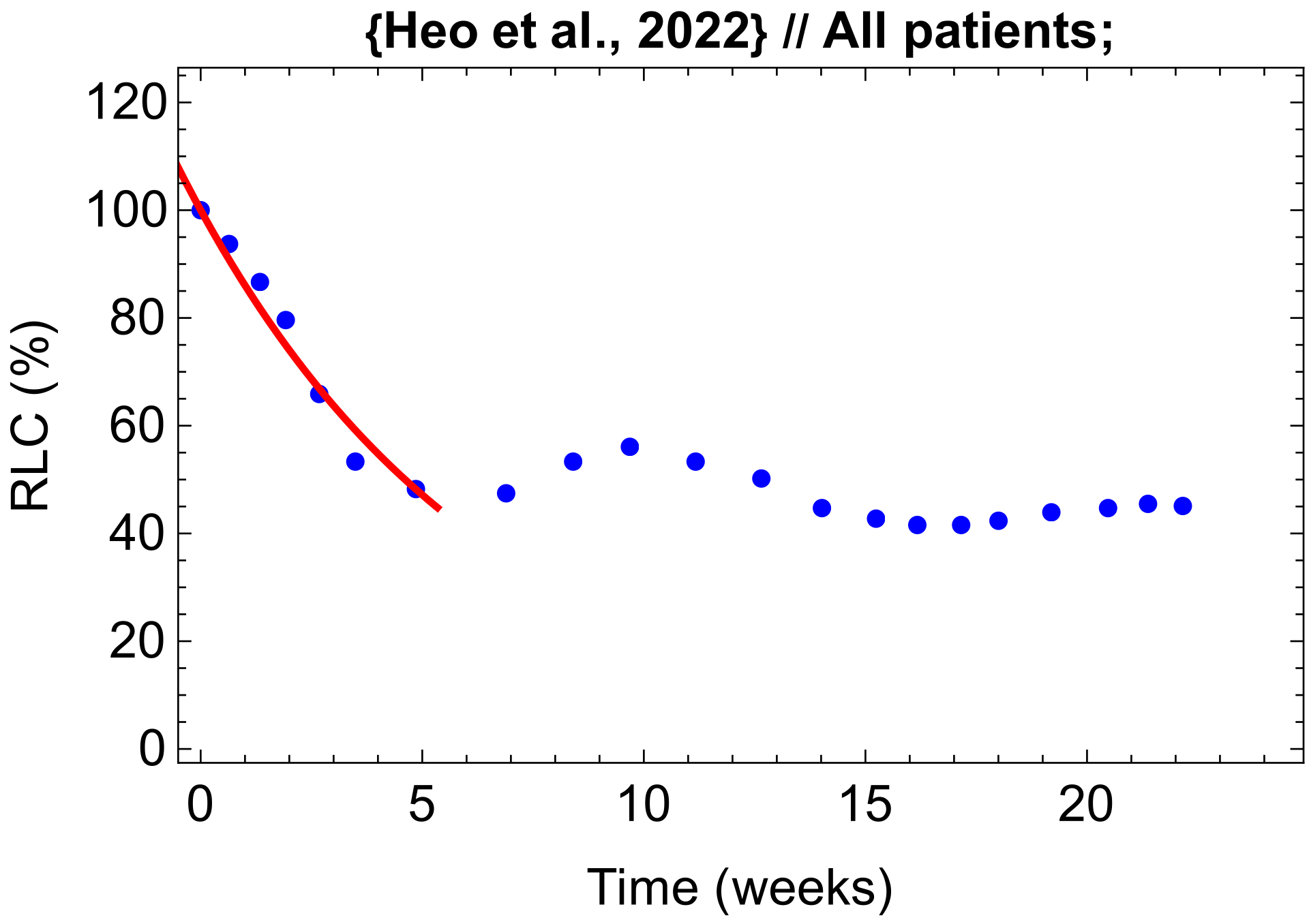

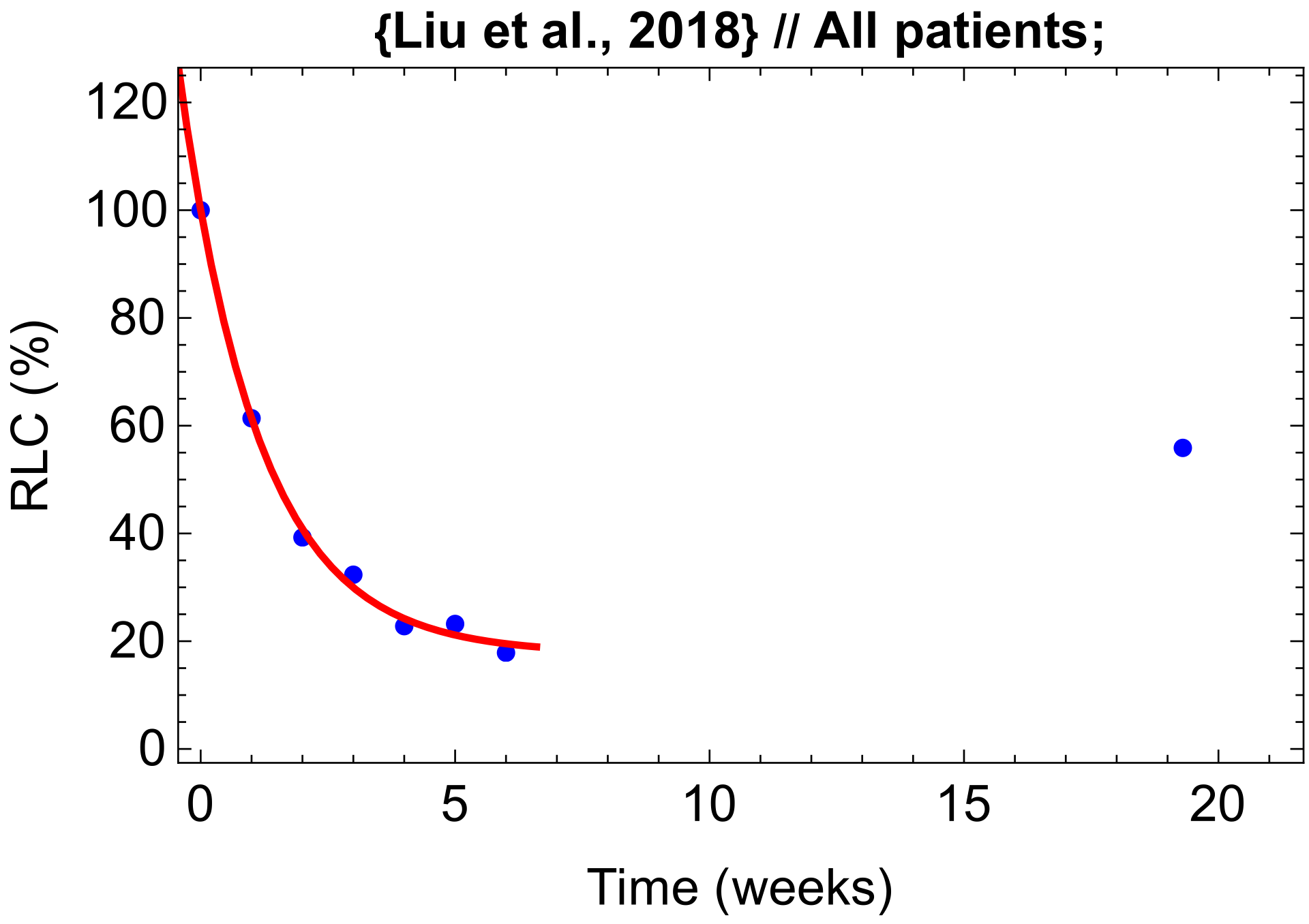

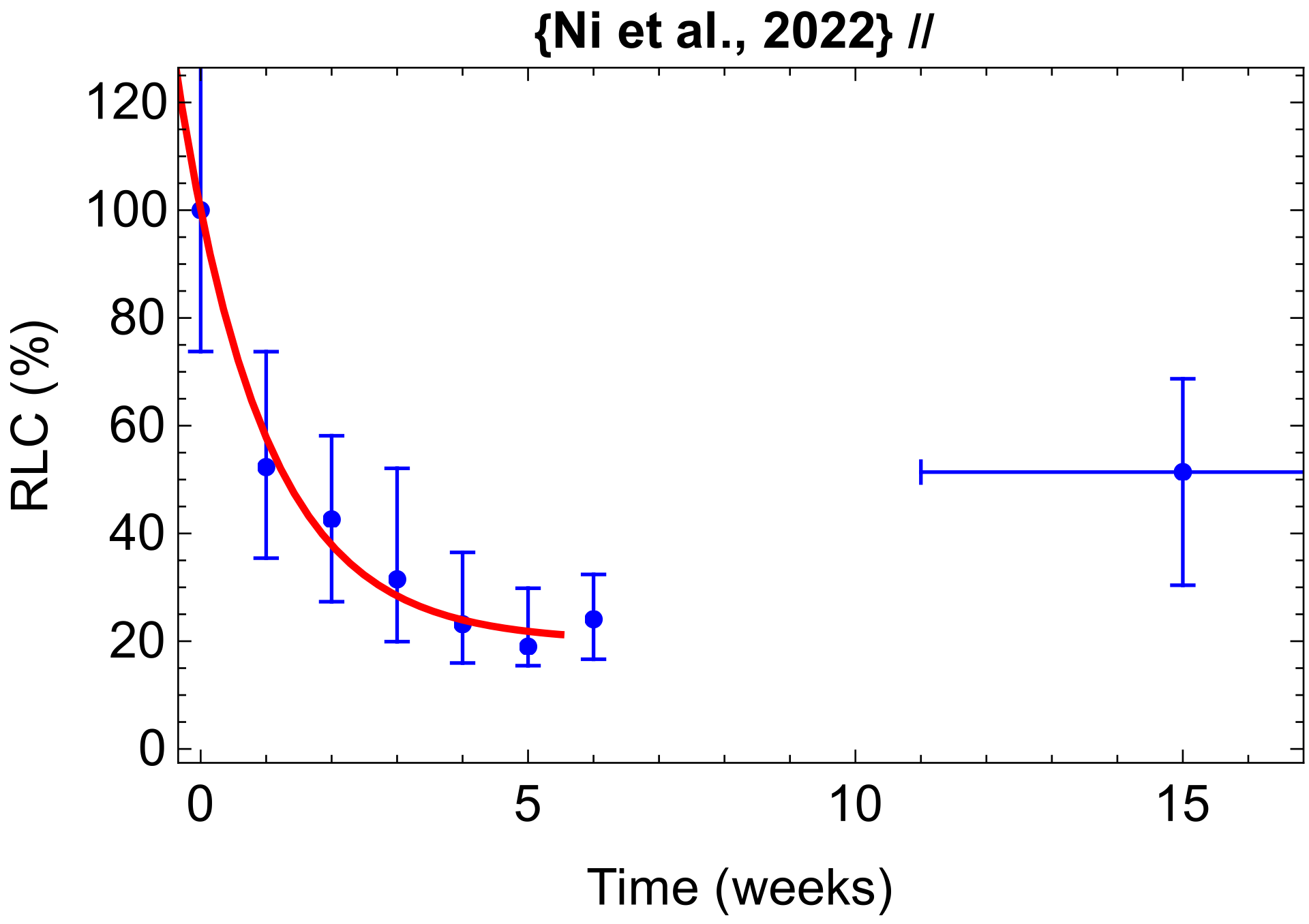

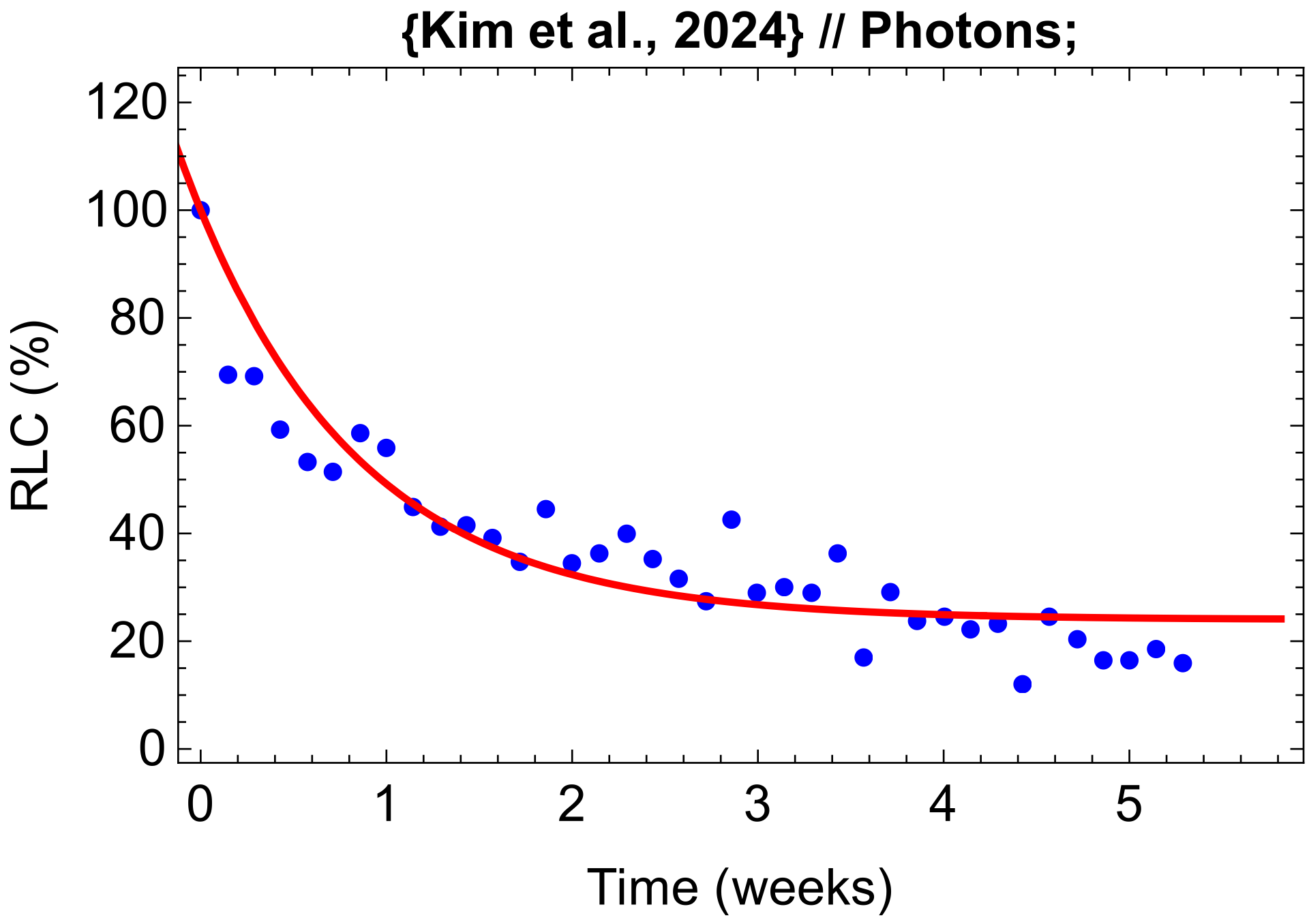

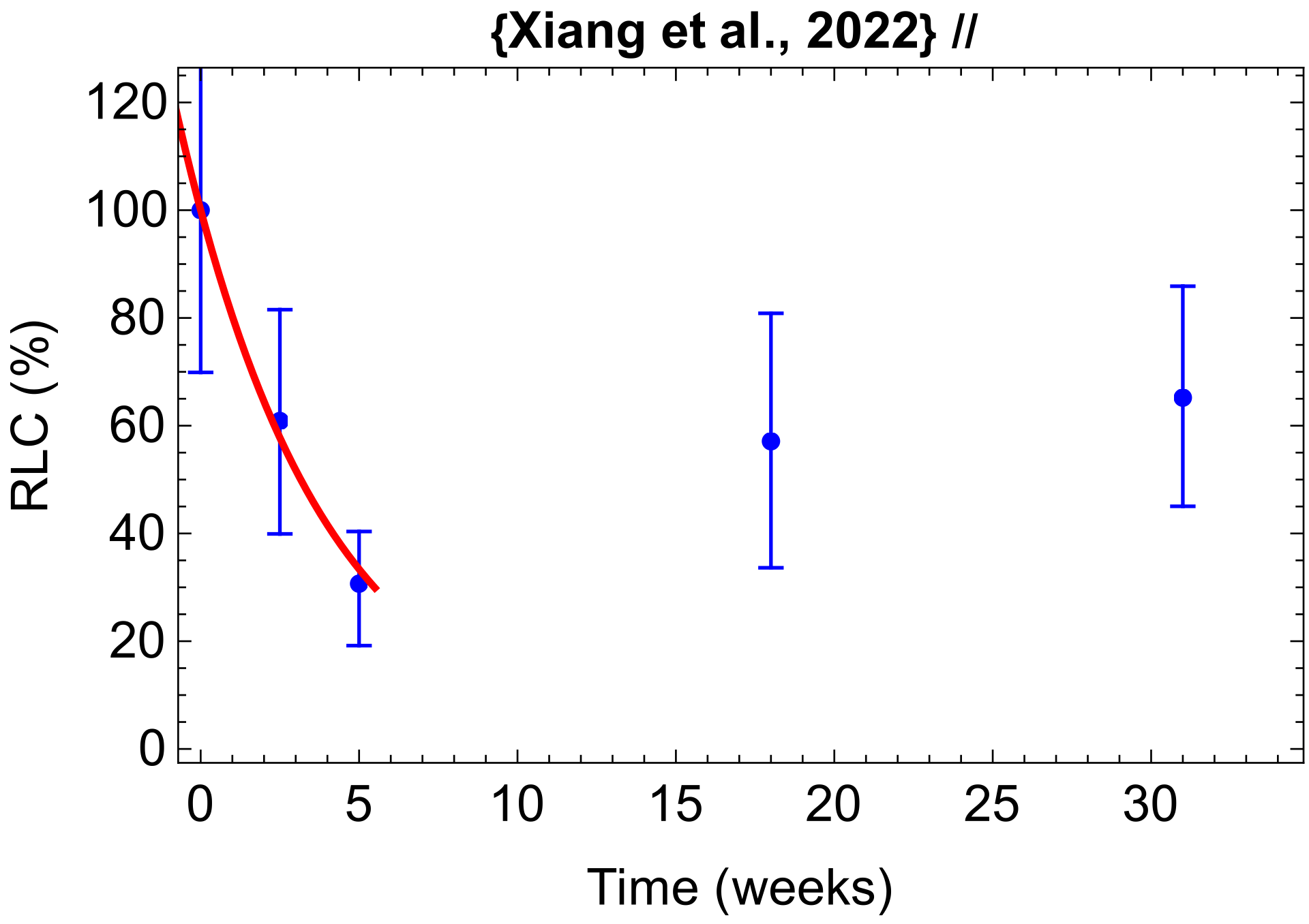

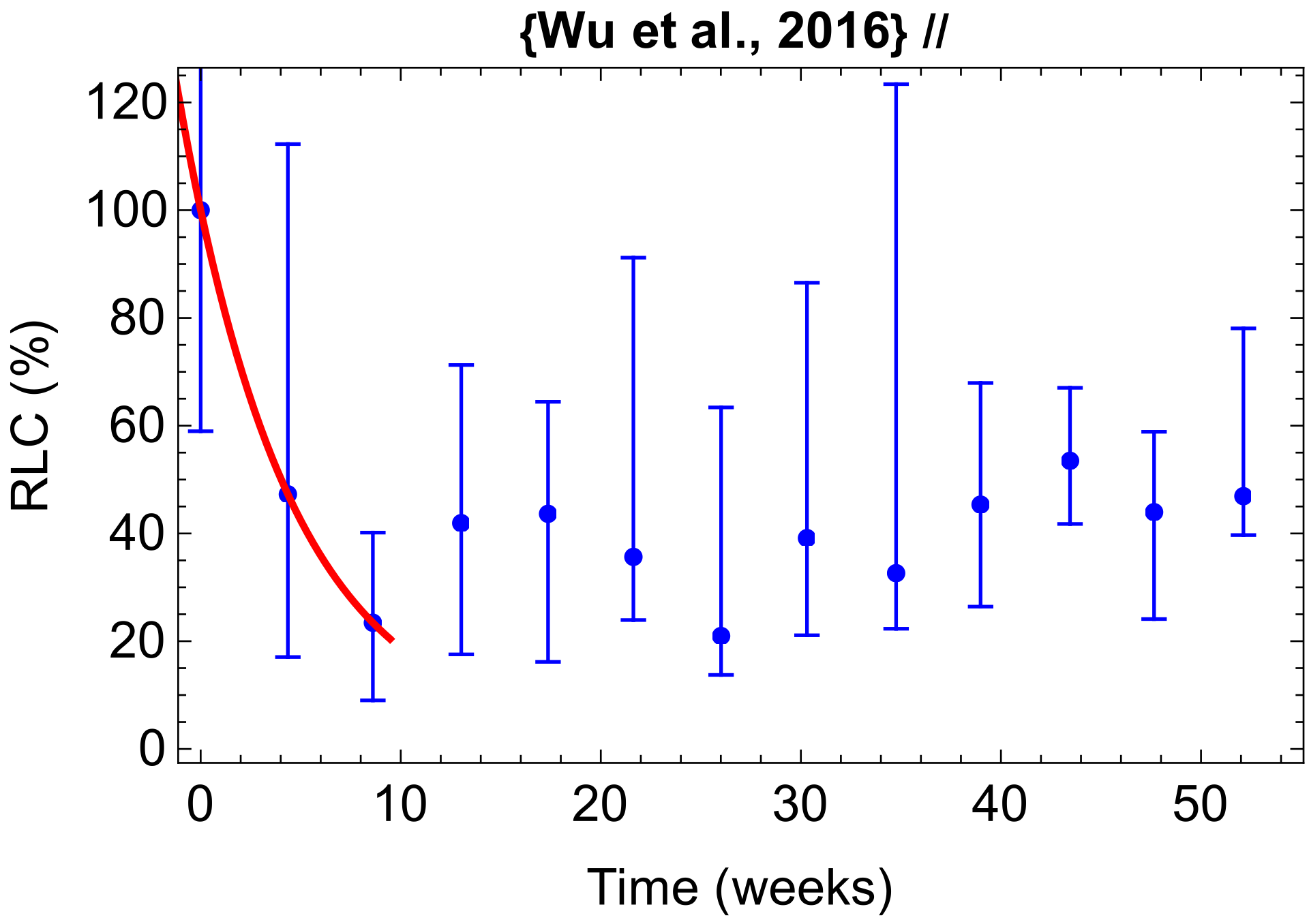

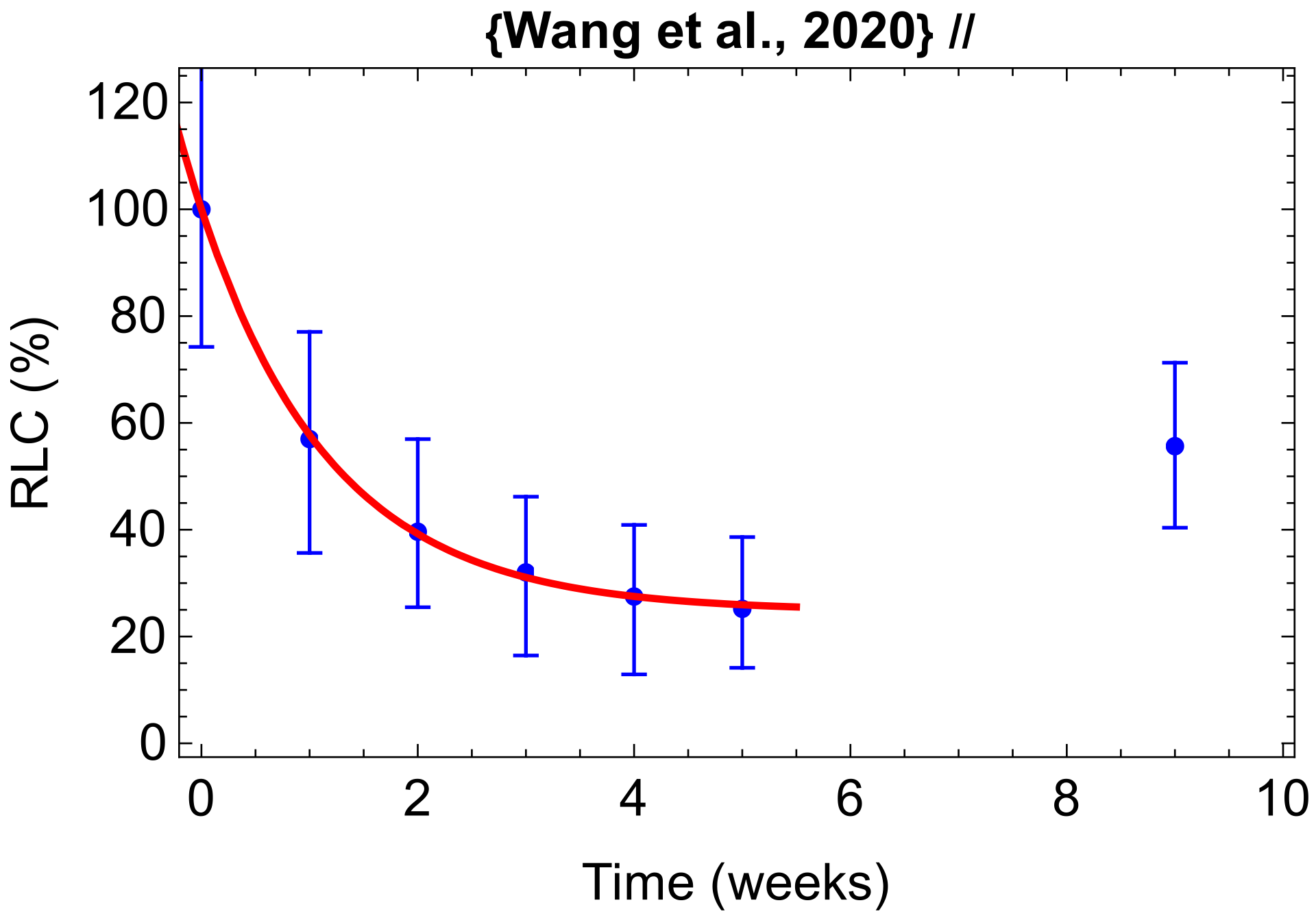

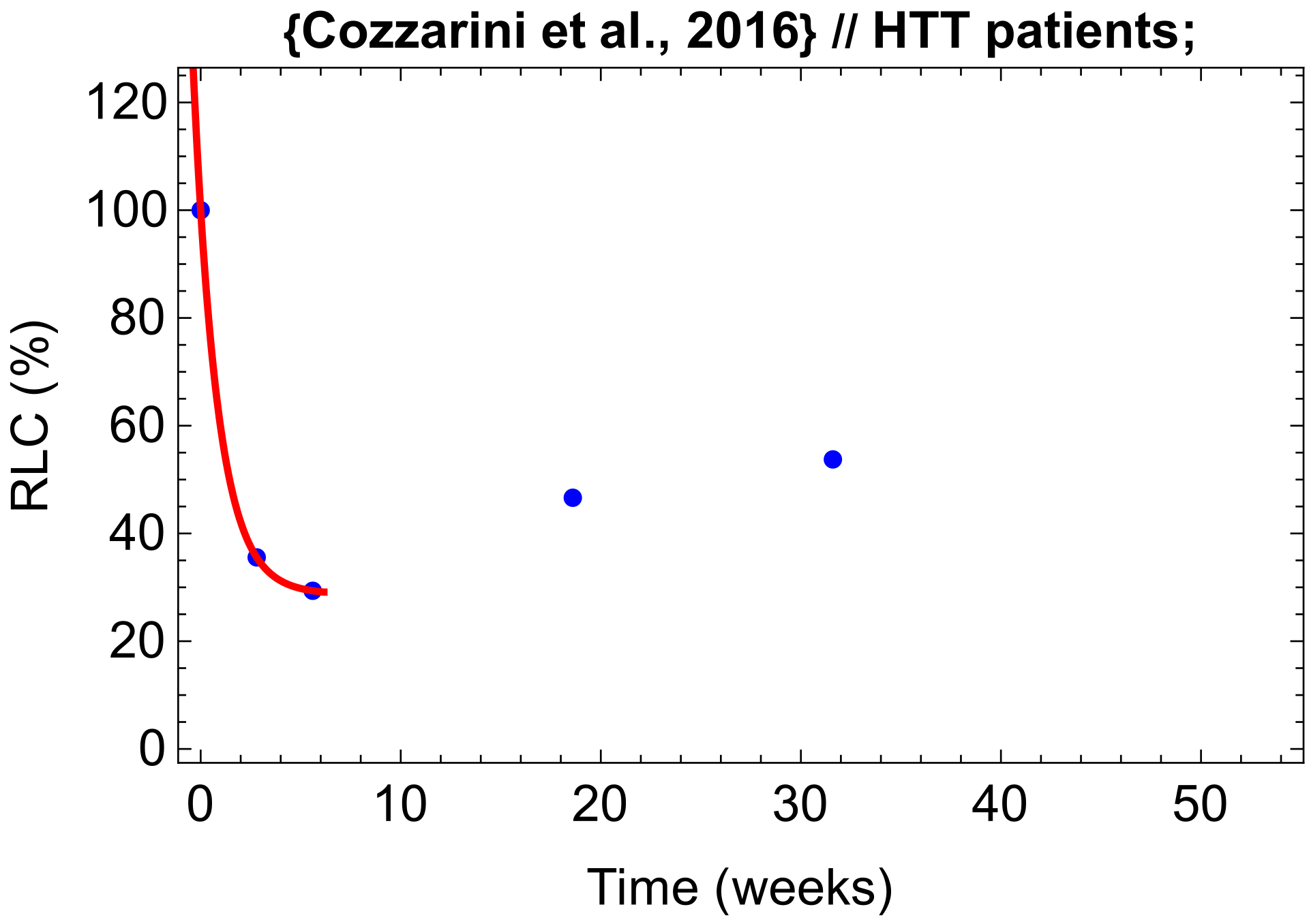

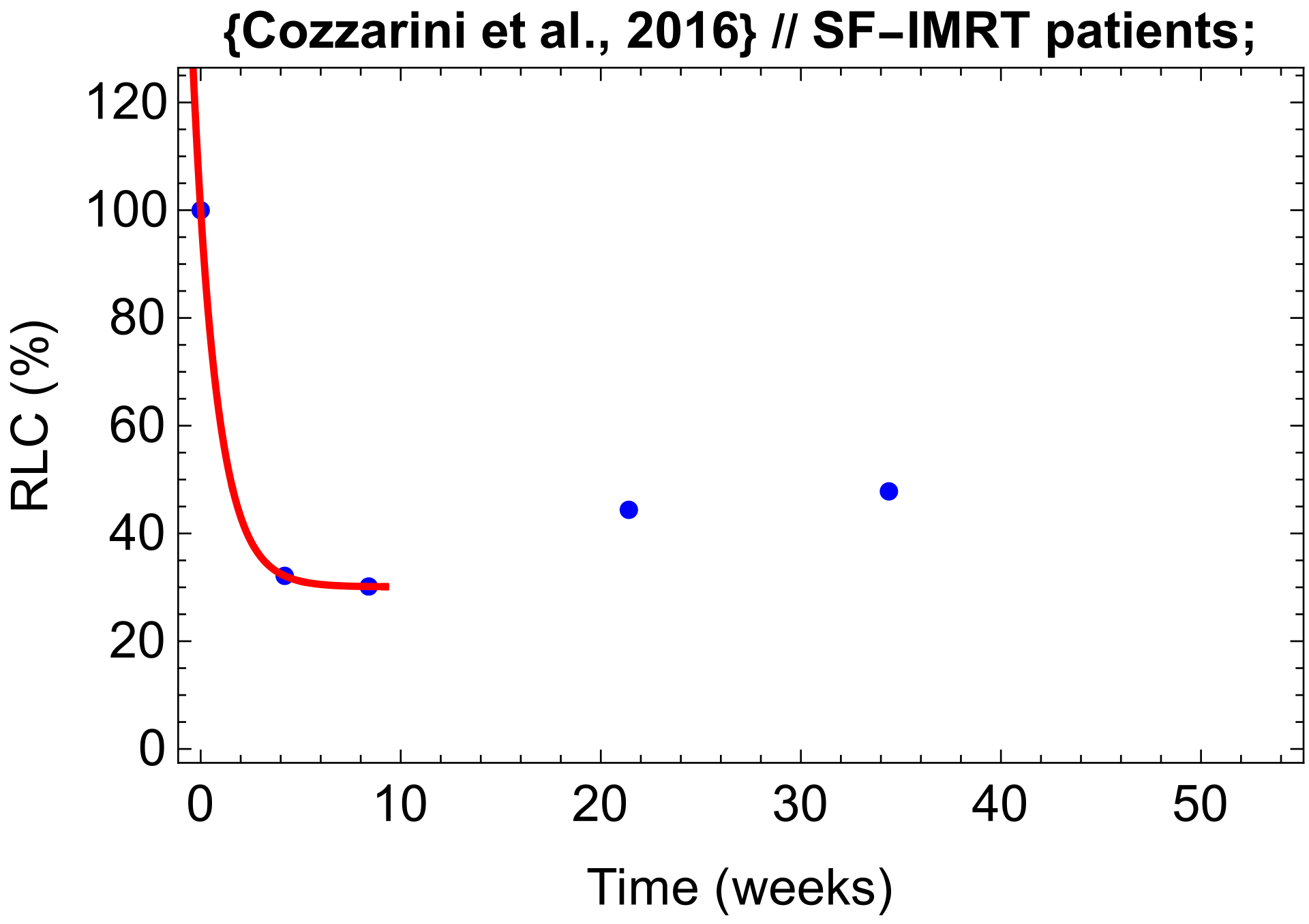

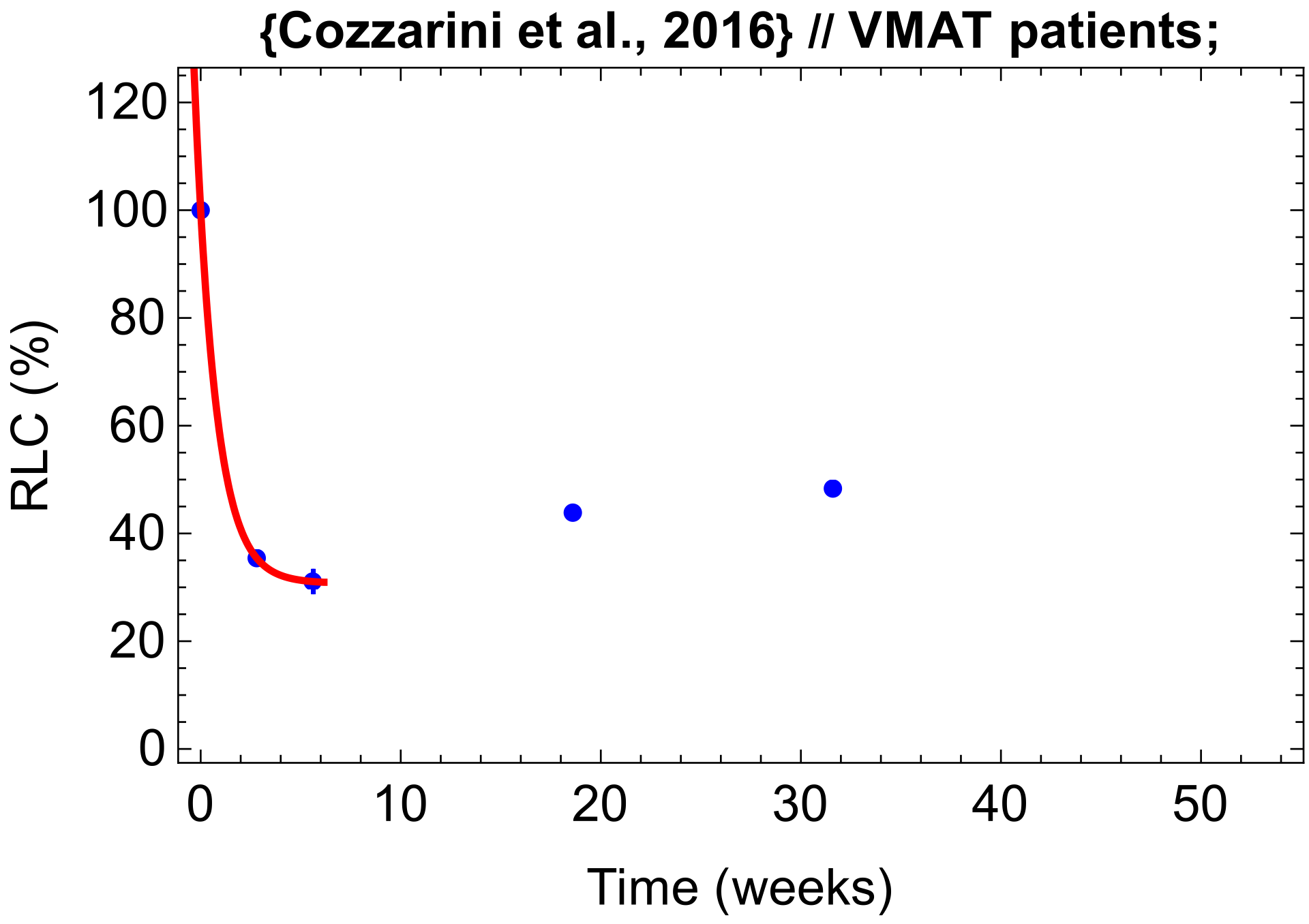

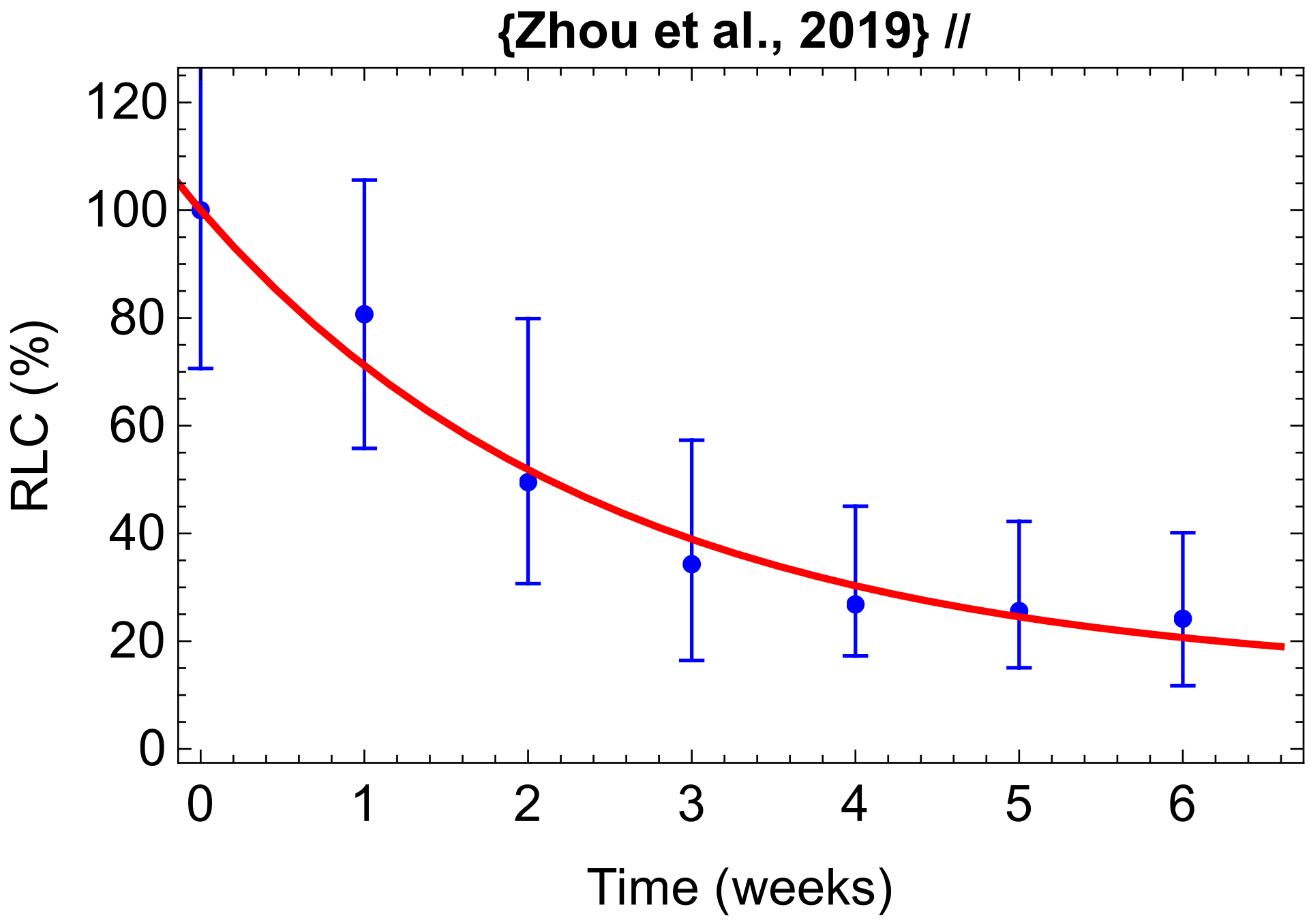

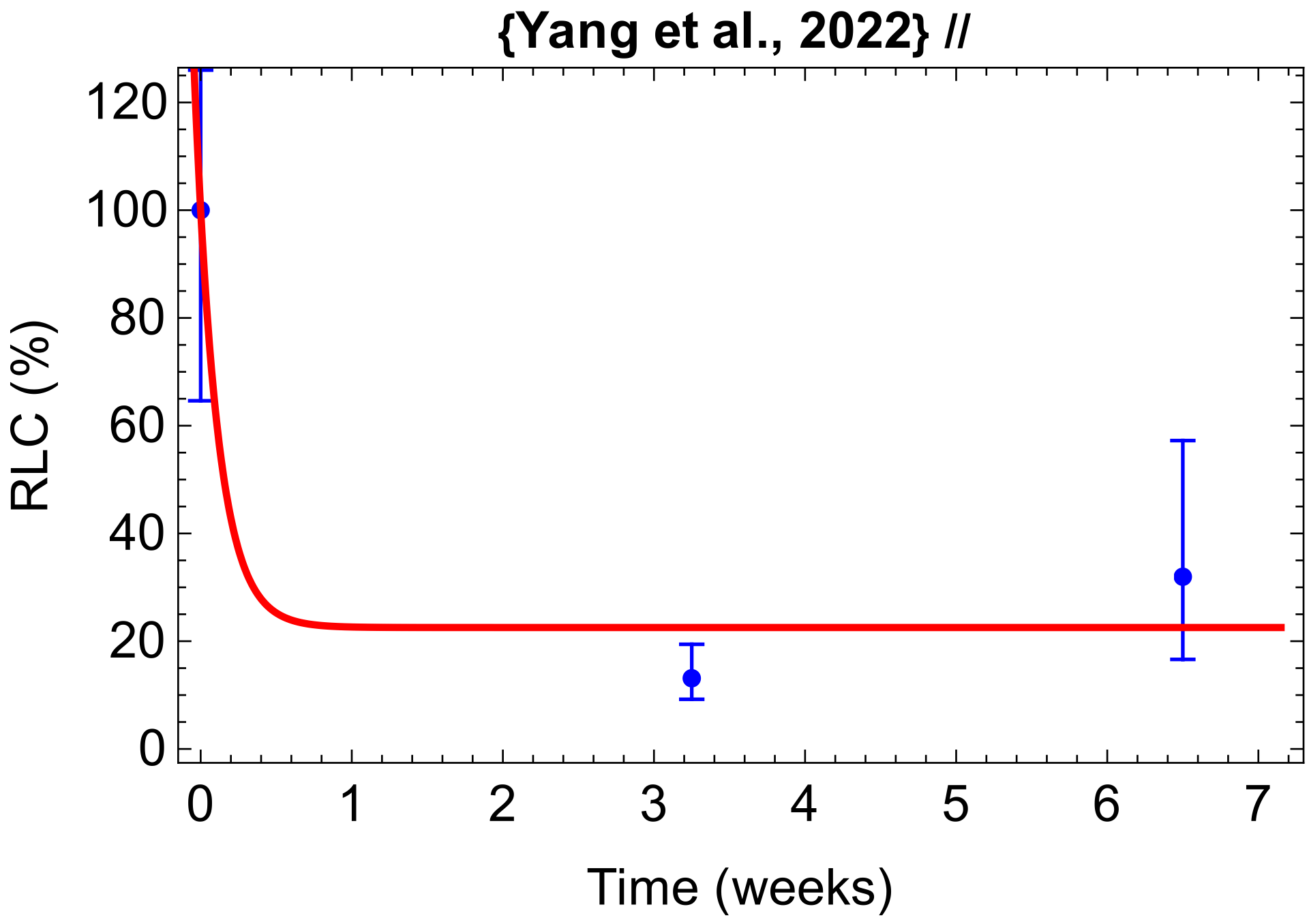

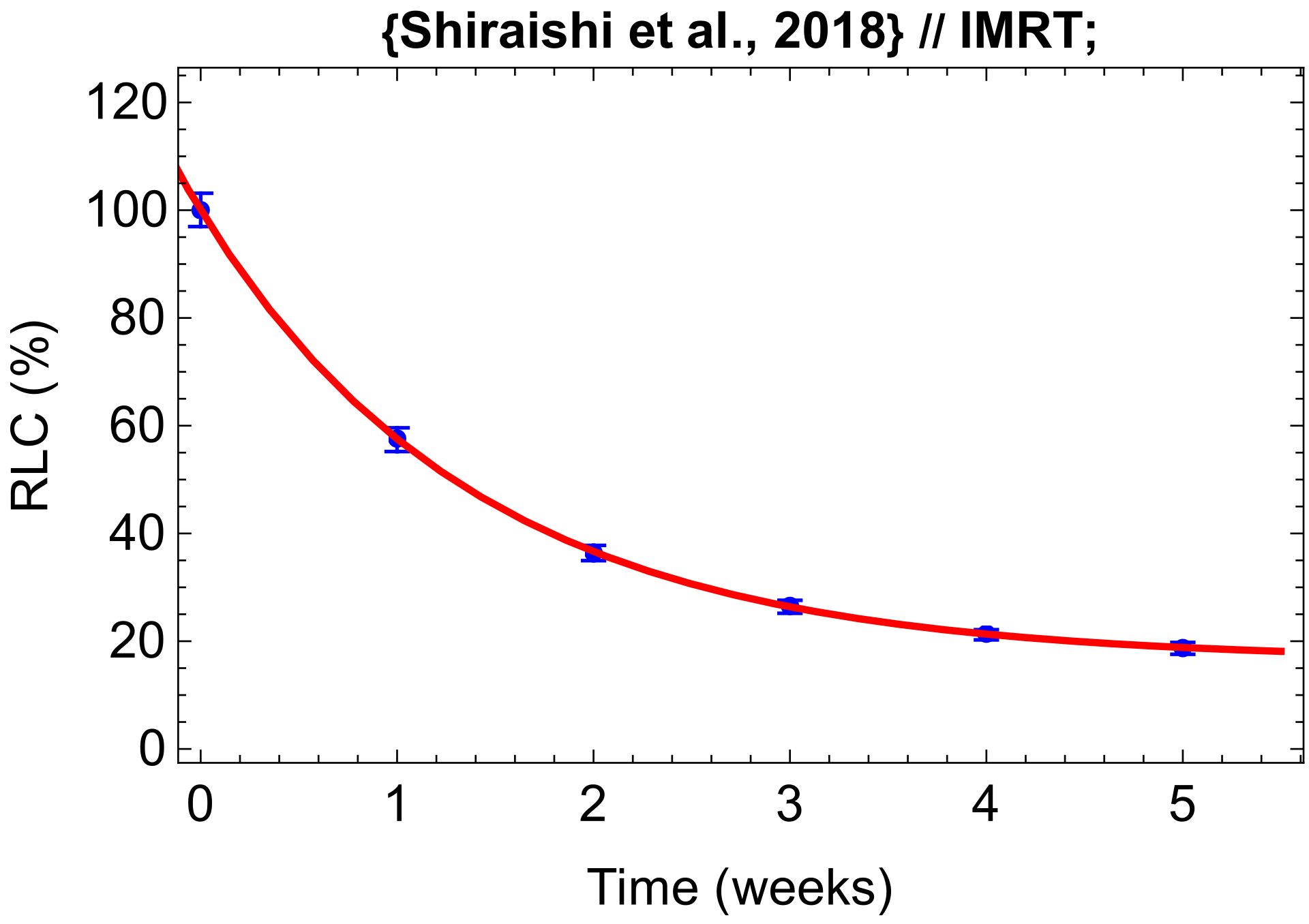

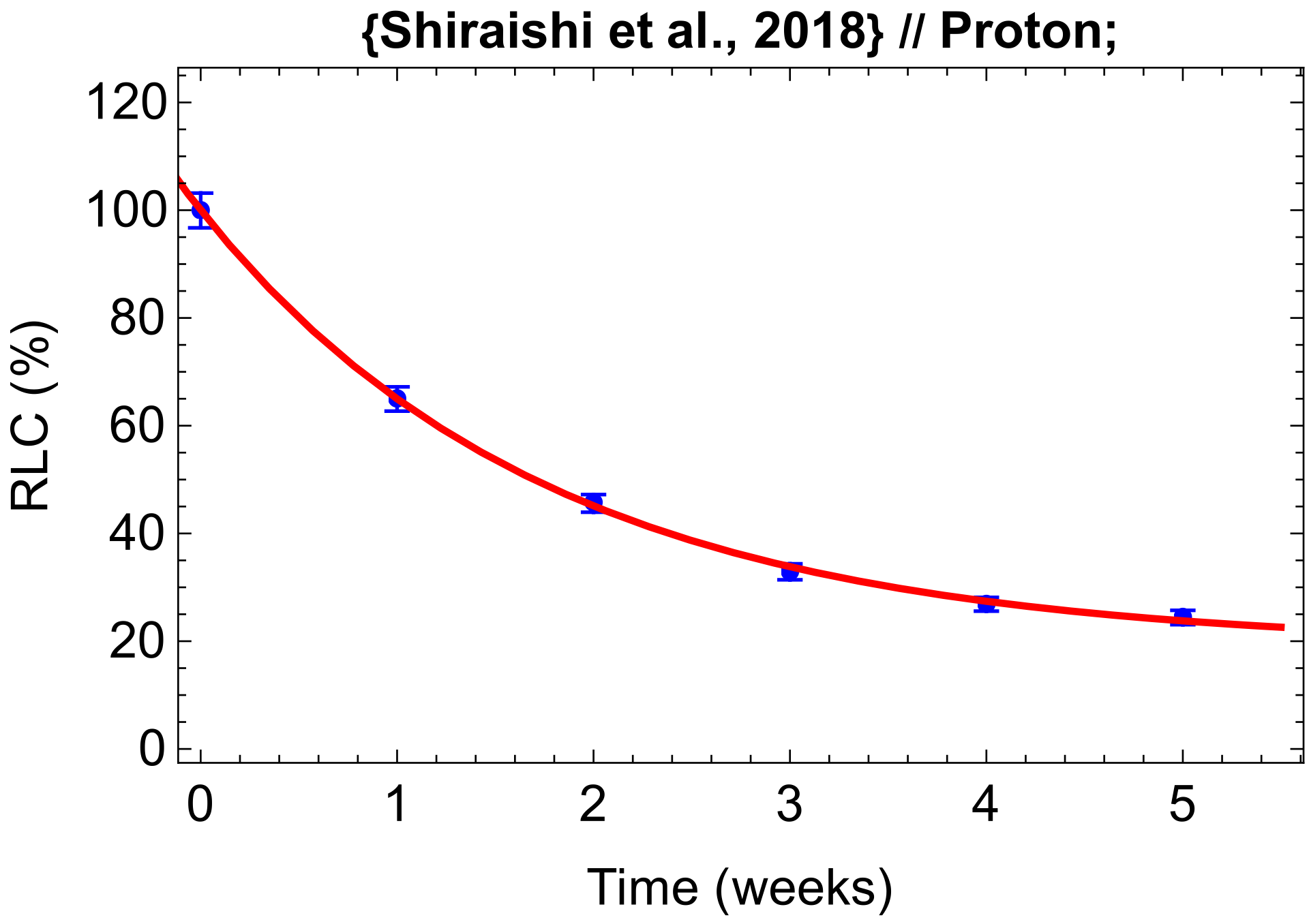

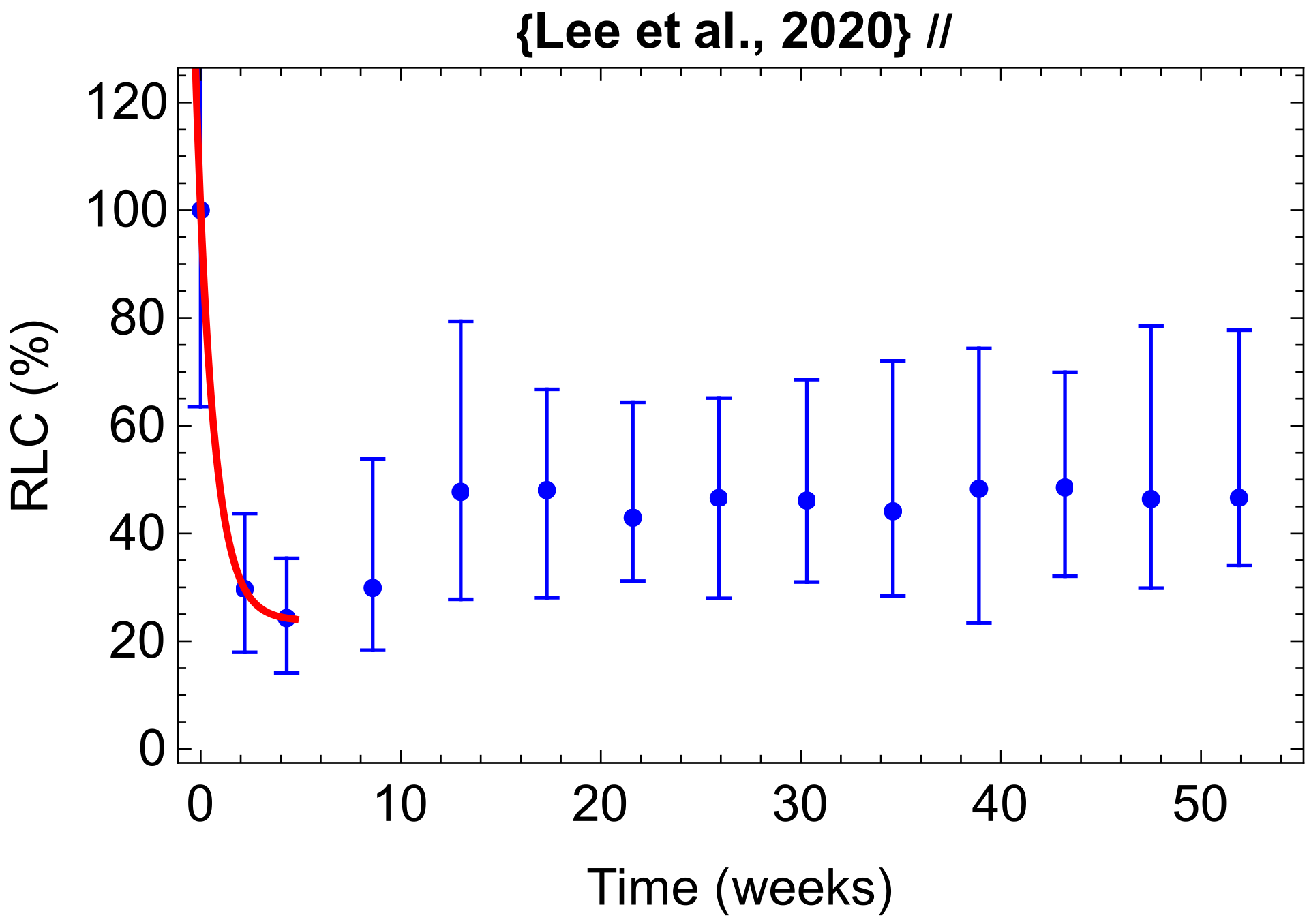

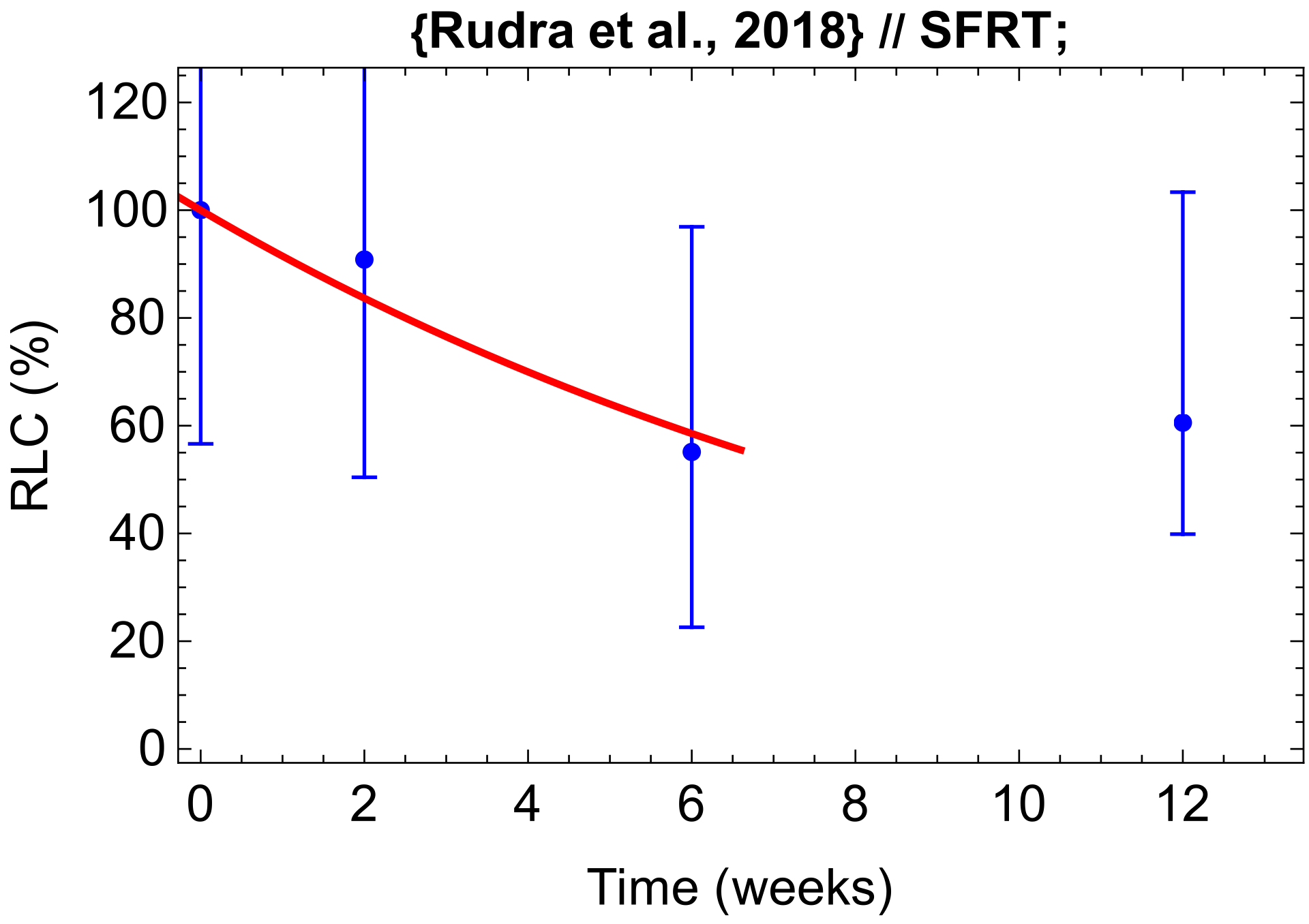

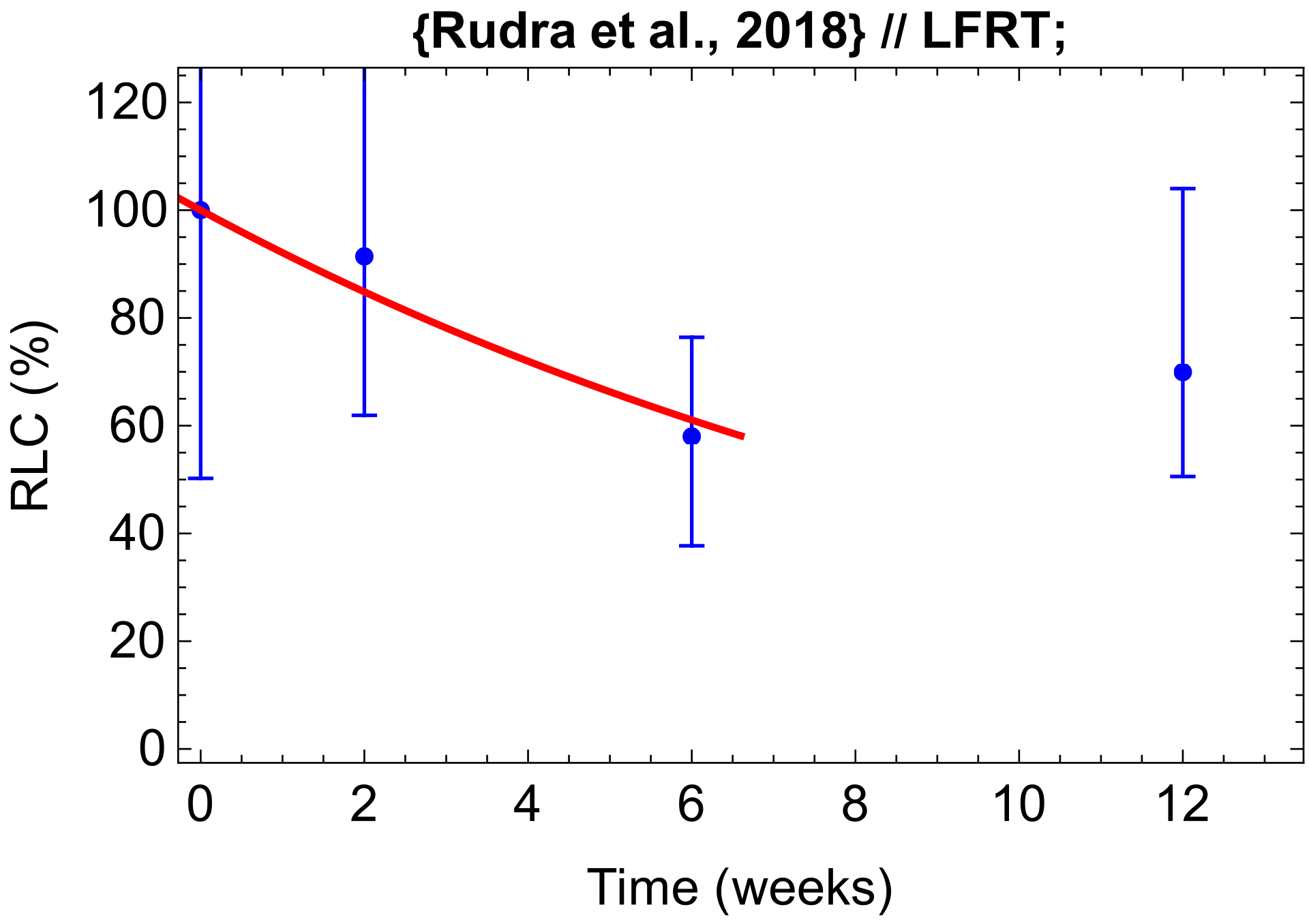

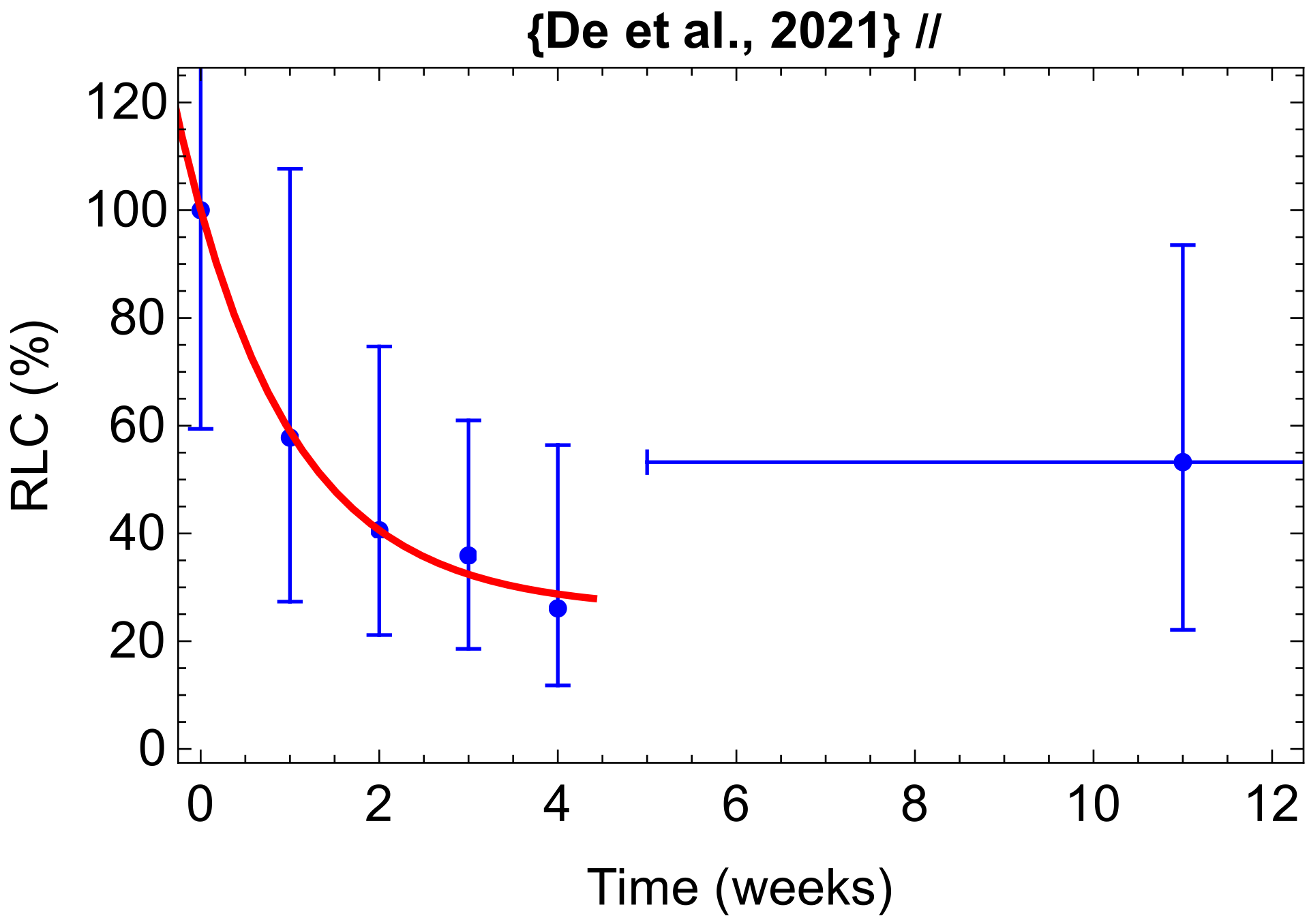

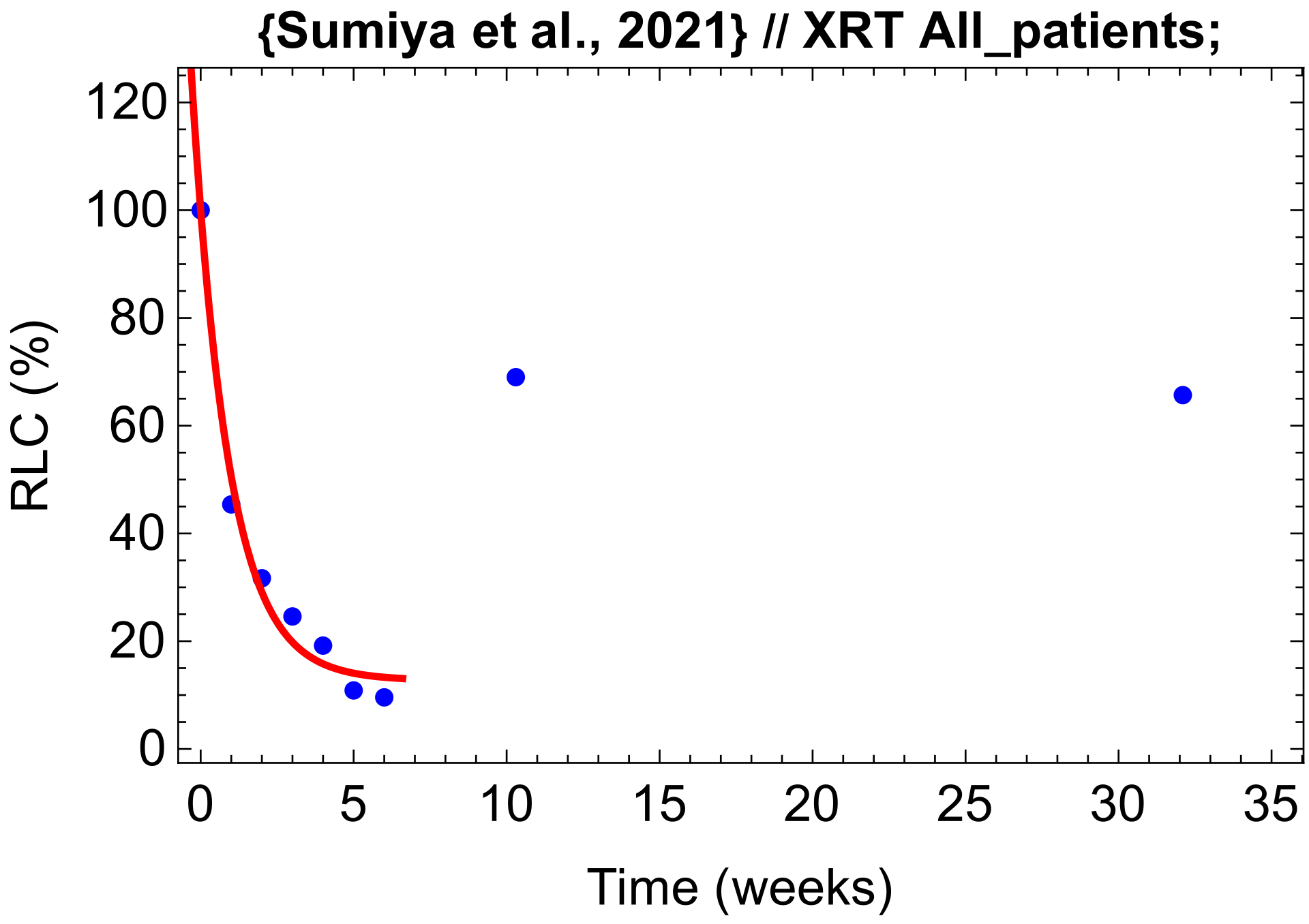

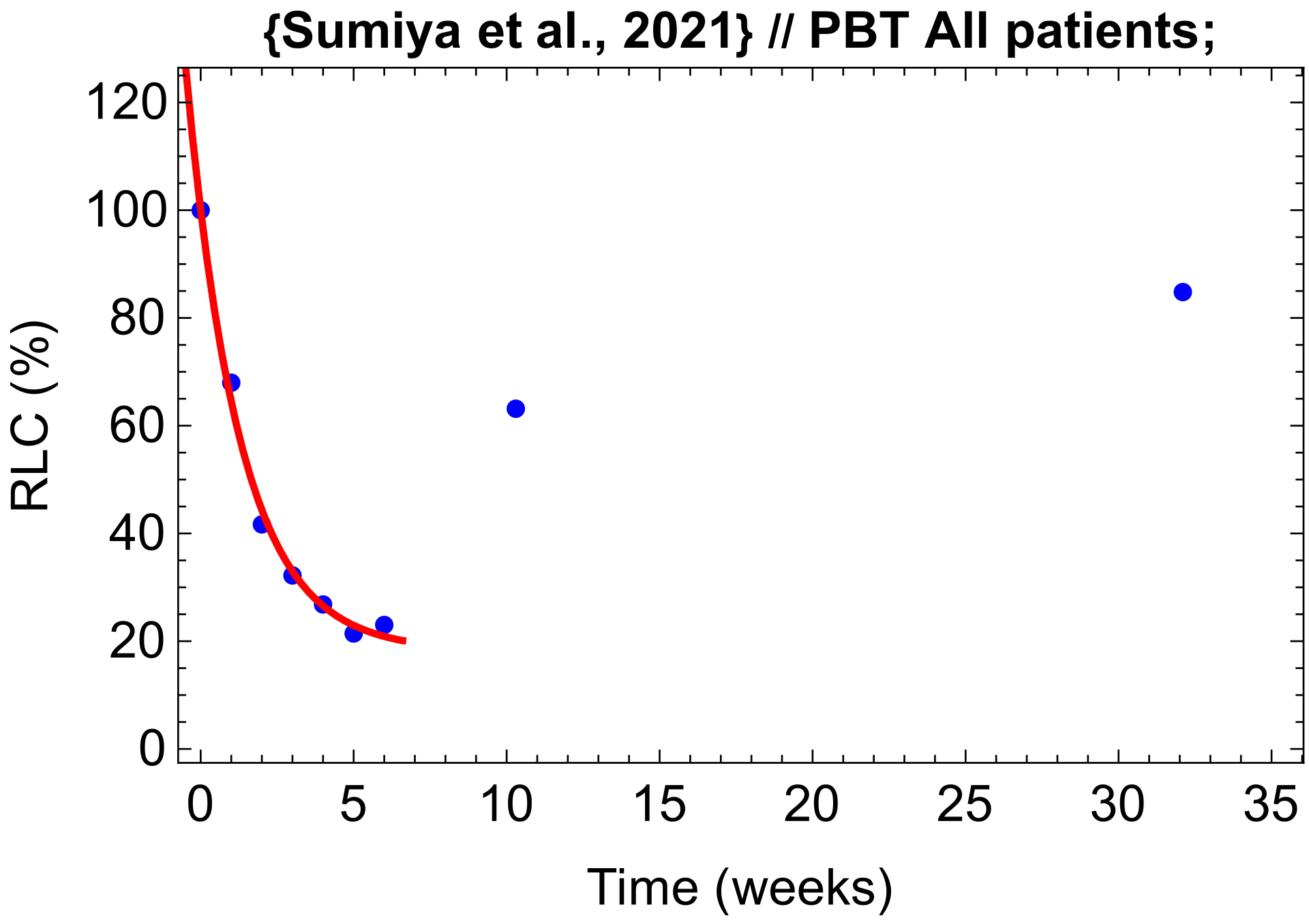


**Fig. A3.** Exponential fits of the selected RLC datasets.

**7. Baseline and EoT ALC and correlations between them across different cancer sites**

**Table A5.** Baseline and EoT-ALCs and their correlation across cancer sites. The last column shows the number of datasets. The p-value for the Pearson correlation coefficient *r* was calculated using a t-test, based on the statistical distribution of *r*, to assess its significance.

| **Irradiated site / organ** | **Median baseline ALC (range), cell/nl** | **Median EoT-ALC (range), cell/nl** | **Pearson correlation coefficient between baseline ALC and EoT-ALC (p-value)** | **N_d_** |
| --- | --- | --- | --- | --- |
| Anal | 1.8 (1.8–1.8) | 0.4 (0.4–0.4) | - | 1 |
| Breast | 1.3 (1.0–1.6) | 0.8 (0.6–1.0) | - | 2 |
| Brain | 1.4 (1.1–1.7) | 0.8 (0.7–1.3) | 0.75 (0.14) | 5 |
| Esophagus | 1.6 (1.4–2.0) | 0.4 (0.1–0.6) | 0.6 (0.01) | 17 |
| Head and Neck | 1.7 (1.7–1.8) | 0.4 (0.3–0.5) | -0.32 (0.68) | 4 |
| Liver | 1.1 (1.1–1.4) | 0.3 (0.2–0.3) | -0.96 (0.18) | 3 |
| Lung | 2.0 (1.5–2.4) | 0.4 (0.2–0.5) | 0.24 (0.57) | 8 |
| Pancreas | 1.7 (1.5–2.0) | 0.7 (0.4–0.9) | 0.45 (0.45) | 5 |
| Pelvis | 1.6 (1.5–1.7) | 0.4 (0.3–0.6) | 0.73 (0.48) | 3 |
| Soft tissue | 1.7 (1.7–1.7) | 0.6 (0.6–0.6) | - | 1 |
| Bones in various sites | 1.0 (1.0–1.0) | 0.5 (0.5–0.5) | - | 1 |

Statistical analyses revealed significant correlations between baseline and EoT-ALC levels in patient cohorts with esophageal cancer, with Pearson correlation coefficients of 0.6 (p=0.01). These results suggest that baseline ALC could serve as a predictive marker for post-treatment lymphopenia severity within this group. Other cancer types did not show statistically significant correlations, likely due to sample size limitations, higher variability in therapy schemes, or patient characteristics. Notably, analyzing aggregated patient cohorts as in Fig. 3 may obscure correlations that may exist within each cohort at the individual-patient level.
